# ﻿First report of the order Mysida (Crustacea) in Antarctic marine ice caves, with description of a new species of *Pseudomma* and investigations on the taxonomy, morphology and life habits of *Mysidetes* species

**DOI:** 10.3897/zookeys.1079.76412

**Published:** 2021-12-31

**Authors:** Karl J. Wittmann, Pierre Chevaldonné

**Affiliations:** 1 Department of Environmental Health, Medical University of Vienna, Kinderspitalgasse 15, 1090 Vienna, Austria. Corresponding author Medical University of Vienna Vienna Austria; 2 IMBE, CNRS, IRD, Aix Marseille Université, Avignon Université, Station Marine d’Endoume, Rue de la Batterie des Lions, 13007 Marseille, France Aix Marseille Université Marseille France

**Keywords:** Development, feeding, key to species, life cycle, marine caves, molecular systematics, polar biology, sensory organs

## Abstract

SCUBA diving explorations of three islands off Dumont d’Urville Station at the coast of Adélie Land, East Antarctica, enabled the observation of marine ice caves. Sampling in this unusual habitat yielded a total of three species of Mysidae, altogether previously poorly known or unknown to science. *Pseudommakryotroglodytum***sp. nov.** is described, based on the structure of the antennal scale, telson and on cornea-like lateral portions set off against the main body of eyeplates. *Mysidetesilligi* is re-established at species level after almost a century in synonymy. Re-descriptions are provided for *M.illigi* and *M.hanseni*, based on types and ice cave materials. Keys to the Southern Ocean species of *Pseudomma* and to the world-wide species of *Mysidetes* are given.

Phylogenetic trees are provided for the genera *Pseudomma* and *Mysidetes*. 18S rDNA sequences of *P.kryotroglodytum* differ from GenBank sequences of other *Pseudomma* species. First sequence data are given for species of the genus *Mysidetes*: 18S differs between the two examined species and COI is quite diverse between and within species.

We found previously unknown, probably sensorial structures in these ice cave species: in *P.kryotroglodytum*, the basal segment of the antennula shows a pit-like depression with striated pad on the bottom and a median cyst, connected with the bottom of the eyeplate cleft. *M.illigi* shows a female homologue of the appendix masculina bearing a field of modified setae. Subsequent investigations demonstrated these structures also in species from other habitats.

The feeding apparatus and stomach contents of the three ice cave species point to brushing of small particles (detritus, microalgae) from available surfaces, such as sediment, rock and the ice surface. Differences in the feeding apparatus are very subtle between the two *Mysidetes* species. The high content of fat bodies in *M.hanseni* could help it to survive periods of starvation. The large storage volume of the foregut in *P.kryotroglodytum* points to the collection of food with low nutritional quality and could help to balance strongly fluctuating food availability.

Summer specimens of *M.hanseni* showed a bimodal frequency of developmental stages in the marsupium and bimodal size-frequency distribution of free-living stages. The females with younger brood (embryos) were, on average, larger and carried more marsupial young than those with older brood (nauplioid larvae). All examined incubating and spent females showed (almost) empty foreguts and empty ovarian tubes, suggesting possible semelparity and death following the release of young. The absence of juveniles and immature females from summer samples suggests that growth and accumulation of fat and yolk occur outside ice caves, while such caves could be used by fattened adults as shelter for brooding. A provisional interpretation proposes a biannual life cycle for *M.hanseni*, superimposed with shifted breeding schedules, the latter characterised by early breeding and late breeding females, probably in response to harsh physical and trophic conditions along the continental coast of Antarctica.

## ﻿Introduction

Species of the order Mysida play an important role for the biodiversity of the Southern Ocean. This is highlighted by the census of Petryashov (2014), who listed 64 species from there, 51 of which are endemic. The Antarctic invertebrate fauna generally shows the highest proportion of endemic marine species (Peck 2018) due to millions of years of isolation of the Antarctic as the only continent without shelf connection to other land masses. Amongst Mysida, the Antarctic endemics constitute as much as 4% of the world-wide stock of 1203 acknowledged recent species (original census from 24–08–2021). Thirty-eight Antarctic species could be classified as benthopelagic or benthic, with some reservation due to sparse documentation and/or use of non-closing sampling devices for a number of species. No cave-dwelling mysids are, so far, known from the Southern Ocean and none from the here-documented ice caves.

Our current knowledge on the Antarctic marine biota stems largely from indirect observations (e.g. Remotely Operated Vehicles) and samples obtained by dredging, trawling and fishing. Although modern techniques have greatly improved species discovery rates (e.g. Griffiths 2010), SCUBA diving exploration of the Antarctic benthos remains uncommon, due to the extreme conditions and costly logistics. Yet, many marine habitats and organisms cannot be easily assessed by remote gears. This, for example, is the case for temperate and tropical marine cave faunas (see Harmelin et al. 1985; Pérez et al. 2016), but also for the fauna living below perennial sea ice (Zimmer 1914; Griffiths et al. 2021).

During recent SCUBA diving explorations between 0 and 20 m depth at the Dumont d’Urville (DDU) Station in Adélie Land, East Antarctica, a peculiar habitat – marine underwater ice caves, which meet both the extreme facets of life under ice, together with the darkness and isolation of caves – was surveyed and sampled. Strikingly similar to what prevails elsewhere in shallow-water marine caves (e.g. Ledoyer 1989; Lejeusne and Chevaldonné 2005; Wittmann and Chevaldonné 2017), one main component of the mobile fauna of such ice caves is species of the order Mysida, an order that otherwise does not appear to be conspicuously abundant in the shallow (0–20 m) benthos at DDU.

Ledoyer (1969) inspected the extensive faunistic collections dredged in Adélie Land by Patrick Arnaud in 1961–1965. He reported only two species of Mysida, namely one specimen of *Mysidetesposthon* Holt & Tattersall, 1906, plus several specimens of the more common *Antarctomysismaxima* (Hansen) [in Holt and Tattersall 1906]. All these mysids were obtained from ≥ 60 m depth. To our knowledge, there is no report available on shallow-water mysids from the DDU area. In analogy to the situation in marine caves from lower latitudes, shallow ice caves could provide shelter to escape from visually orientated predators during austral summer and could also provide suitable conditions for deep-water species (Janssen et al. 2013).

The advantages of SCUBA-based collection methods were used to sample mysids in shallow marine ice caves of Adélie Land. Our knowledge of mysid diversity from East Antarctica was deepened by direct *in situ* observations and by the study of freshly-collected material that allowed: (1) description of one new species and re-description of two other species; (2) exploration of their feeding, reproduction and life cycle; (3) description of their habitat when sheltered in shallow-water ice caves and (4) estimation of their DNA sequence affinities by a first molecular taxonomic study.

## ﻿Materials and methods

### ﻿Field materials

**Figure 1.**
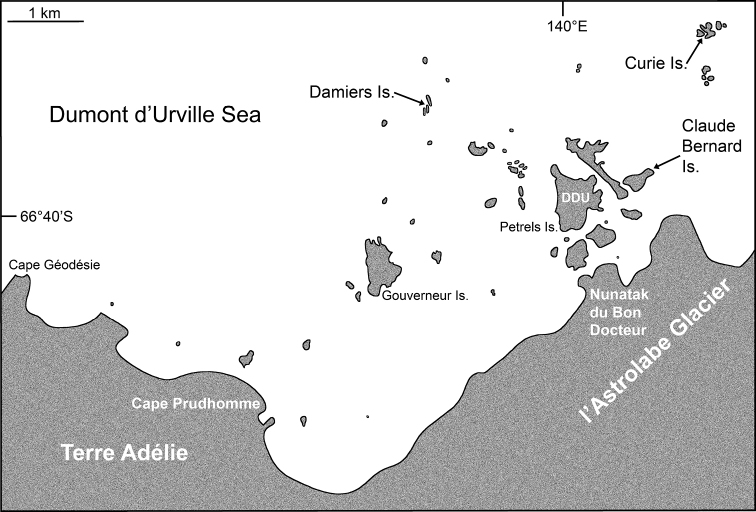
Sampling stations (arrows) in ice caves of three islands near Dumont d’Urville Station (DDU), off Adélie Land, Antarctica.

Samples were collected during the POLARIS programme (2013–2018, Stéphane Hourdez principal investigator) funded by the French Polar Institute (IPEV) in austral summers 2015–2016 and 2017–2018. In the search for ice caves, SCUBA divers (Pierre Chevaldonné [PC], S. Hourdez, S. Castanet, M. Robert, J. Fournier) sailed in small boats to partly ice-covered islands or islets with environmental conditions appearing suitable for ice caves to occur. Such conditions were found in 2016 at Claude Bernard Island (66°39.64'S, 140°01.55'E) and, in 2018, at the Curie Islands (66°38.64'S, 140°02.43'E) and Damiers Islands (66°39.21'S, 139°57.61'E). Each of these sites is located within 1–3 km (Fig. 1) of the main Adélie Land (East Antarctica) permanent station, Dumont d’Urville (DDU). SCUBA divers visually identified mysids and collected them with specially designed suction bottles (Chevaldonné et al. 2008). Mysids were maintained alive until preserved in 95% ethanol. One specimen of *A.maxima* was further obtained on 18 January 2016 from a plankton net operated by colleagues of the Ico²Taks programme (C. Davies, A. Guillou, E. Tavernier) from a hole dug in sea ice over a bottom of ca. 40 m depth, 66°39.79'S, 139°59.65'E, this just being west of the Petrels Island, at the DDU station (Fig. 1).

### ﻿Collection materials

Previously unknown features, detected in ice cave specimens, were checked for potential presence in other species of the respective subfamilies. This includes type materials of *Mysidetes* Holt & Tattersall, 1906 species obtained on loan from the Zoological Museum Berlin. Other important museum materials were already on desk for ongoing studies of expedition collections. Some non-types were obtained in the frame of statolith studies (e.g. Wittmann et al. 1993; Wittmann and Ariani 2019) in the 1980s by exchange of collection materials with Torleiv Brattegard (Bergen), Masaaki Murano (Tokyo) and the meanwhile deceased John Mauchline (1933–2013) (Oban).

### ﻿Repositories

**NHMW** Natural History Museum of Vienna;

**SMF**Senckenberg Museum, Frankfurt am Main;

**ZMB**Zoological Museum Berlin;

**ZMH**Zoological Museum Hamburg.

Types of *P.kryotroglodytum* sp. nov. are deposited at NHMW. Non-types of two *Mysidetes* species are deposited at all these institutions, with some material retained for future studies.

### ﻿Terminology

Most terminological items are as given in Wittmann and Abed-Navandi (2021). Gross morphology is according to Tattersall and Tattersall (1951). With certain modifications, as stated by Wittmann (2000), appendage terminology is according to Tattersall and Tattersall (1951) and for non-sensory cuticle structures, according to Klepal and Kastner (1980). Terminology of gross structures of the foregut follows Kobusch (1998), modiﬁed spines of the foregut Wittmann and Griffiths (2018). According to Wilson (1989), the term ‘whip seta’ is used for setae with the basal part (handle) bearing a thin flagellum (cord, sensory part) at its tip; handle and cord are separated by a suture or other kind of articulation.

Working terms are used for structures previously unknown in species of Mysidae: ‘eyeplate cyst’ for median cyst connected with the bottom of the eyeplate cleft; ‘female antennular lobe’ for female homologue of the appendix masculina; ‘antennular depression’ for pit-like depression with dorsal opening about centrally on the basal segment of the antennula, not to be confounded with the Tattersall organ in more proximal position close to eye rudiments in certain Petalophthalmidae (see Discussion).

### ﻿Definition and abbreviation of stages

We propose a consistent, strict distinction of stages and distinguish more stages and substages than the most widespread, traditional scheme by Tattersall and Tattersall (1951):

Embryonic and larval stages are distinguished essentially according to Wittmann (1981):

**E0** unfertilised eggs;

**E1** to **E6** embryos (eggs) at substage E1 freshly fertilised, up to E6 with the embryonic abdomen folded back over the germ, ready for shedding the egg membrane;

**N1** to **N4** nauplioid larvae at substage N1, freshly hatched from the egg membrane, up to N4 for those shortly before the moult leading to the postnauplioid stage;

**P1** to **P3** postnauplioid larvae at substage P1 freshly moulted, up to P3 that lasts until moult to juvenile stage upon or shortly after release from brood pouch.

Detailed definitions are here proposed for free-living stages arranged according to sex and typical succession:

J juveniles: no external sexual characteristics;

♂I immature males: short (rudimentary) penes externally visible; appendix masculina, if any, externally visible as small non-setose knob;

♂S subadult males: penes well developed, not necessarily at final size, spermatozoa occasionally visible in efferent ducts; appendix masculina not or sparsely setose; dimorphic pleopods, if any, imperfectly developed;

♂A adult males: penes fully developed, spermatozoa mostly visible in efferent ducts; appendix masculina well setose; dimorphic pleopods, if any, fully developed;

♀I immature females: oostegites rudimentary though distinct, not overlapping; ovarian tubes rudimentary, though visible through the transparent body;

♀S subadult females: oostegites overlapping, not yet forming a compact chamber; ovarian tubes fully developed, may be filled with yolk depending on ovarian and breeding cycles;

♀A adult females: marsupium represents a compact chamber by overlap of oostegites, ventral slit covered by interlocking setae; ovarian tubes filled with yolk depending on ovarian and breeding cycles;

♀B brooding (incubating) females;

♀U, ♀E adults with unfertilised or fertilised eggs in the marsupium;

♀N, ♀P adults with nauplioids or postnauplioids in the marsupium;

♀0 adult females with empty marsupium, represented by non-incubating reproductive females (♀0^+^) with yolk in ovarian tubes and by non-reproductive (spent) females (♀0–) without yolk;

+ superscript indicating yolk in ovarian tubes;

– superscript for empty ovarian tubes.

### ﻿Additional abbreviations

**BL** body length.

**S**# sample number in Suppl. material 1.

### ﻿Documentation

Colour photos of live specimens were made by one of us (PC) and Stéphane Hourdez in the field and in laboratories of the Dumont d’Urville Station. Half-tone microphotography was performed by KJW on ethanol-fixed entire specimens in vial and on dissected parts mounted on slides. Entire objects were studied and photographed using 15× to 112.5× standard episcopic microscopy, dissected objects with 40× to 1000 × phase contrast diascopic microscopy. Electronic pencil drawings were made using stacked photos as models.

Description schemes as in Wittmann and Abed-Navandi (2021). Photos and drawings of sex-specific features of *Mysidetes* species are labelled by symbols for females or males. The absence of such labels implies absent or unapparent sex-specific differences.

### ﻿Measurements, preparation and microscopy

Body length (BL) was measured according to Tattersall and Tattersall (1951) from tip of rostrum to terminal margin of telson without spines and setae. Wherever practical, length measurements of antennae, eyestalks, carapace etc. were made along the dorsal mid-line.

Depending on availability, 2–4 (sub)-adult specimens per species were dissected completely. The ethanol-fixed specimens were dissected and the parts mounted in Swann’s (= Swan) medium on slides. The medium was hardened and the objects bleached for 20 h at 60 °C. Bleaching continued for several weeks at room temperature. Slides were sealed tightly several months later.

For the study of small cuticle structures, the carapace together with, if present, the eyeplate, were detached from the body. The cuticle of the pleon was cut along the ventral mid-line and then skinned off. All these preparations were then expanded on slides with dorsal face up. Due to the strength and elasticity of the pleon cuticle, some lateral portions unintentionally whipped back underneath the flattened skin. In such cases, the pleopods became positioned below the drawing plane in Figs 6F, 17L. The statolith structure was examined as detailed in Wittmann et al. (1993).

We examined the available summer materials in detail by checking the body for oil globules and the ovarian tubes for the presence of yolk. We qualitatively and quantitatively estimated the foregut contents in addition to the usual counts and measurements of marsupial and free-living stages. The degrees of filling of foreguts and ovarian tubes were checked through the semi-transparent cuticle by properly adjusting the light source. Qualitative data were obtained by smearing foregut contents on to the slide. The presence of oil globules in the body was checked only from photos of living specimens because lipids dissolve in 95% ethanol-preserved specimens. Most eggs and larvae were removed from the marsupium for counting, size measurements and determining the state of development. A few were left *in loco* for potential future examination.

### ﻿Statistics

The programme XLSTAT 2021 version 23.2.1140, edited by Addinsoft, was used as an add-in of MS-Excel sheets for standard statistics. *Χ*^2^-tests were applied for nominal variables (frequency of stages); neighbouring items with n < 5 were pooled. Student’s t-tests were used for scale variables (body size and numbers of young) and Fishers F-tests for differences between variances (numbers of embryos versus nauplioid larvae). The Anderson-Darling-Test was used to check for normal distribution as a prerequisite for the Grubbs-Test in outlier analysis of length-frequency distributions.

### ﻿Molecular study

Small parts (appendages of the two type specimens of *P.kryotroglodytum* sp. nov.) and entire or half specimens (*Mysidetes* spp., *A.maxima*) were selected for DNA extraction, followed by PCR amplification of fragments of the 18S and COI genes as in Chevaldonné et al. (2015). PCR reactions were then sequenced by Eurofins, Germany. Consensus sequences from sequencing both strands were used and deposited in the GenBank database (Accession numbers OK351312 – OK351330; OK353676 – OK353694).

Sequences were aligned and analysed in terms of % divergence and genetic distance calculated as Kimura 2-parameters (K2P). For *Mysidetes*, a distance tree (NJ) was built for our COI data alone (*A.maxima* as a root) with CLUSTALX 2.1 (Larkin et al. 2007) with bootstrapping support assessed over 1000 replicates. Coding COI sequences were translated into amino acids to check for stop codons and to dismiss the possibility of obvious pseudogenes. There were no indels at the 18S locus within our *Mysidetes* dataset. There are no other sequences of *Mysidetes* available in databases.

For *Pseudomma* G.O. Sars, 1870, the COI barcodes used here could not be aligned with the sequences currently available in GenBank (different parts of the gene). Available 18S sequences of other species of *Pseudomma* were aligned with *P.kryotroglodytum* sp. nov. to build phylogenetic trees (NJ and ML). Maximum Likelihood (ML) trees were obtained with PHYML 3.0 (Guindon et al. 2010; Lefort et al. 2017) with bootstrapping support assessed over 1000 replicates. There were no indels in that 18S dataset.

## ﻿Habitat

### ﻿Physical aspect and fauna of ice caves

**Figure 2.**
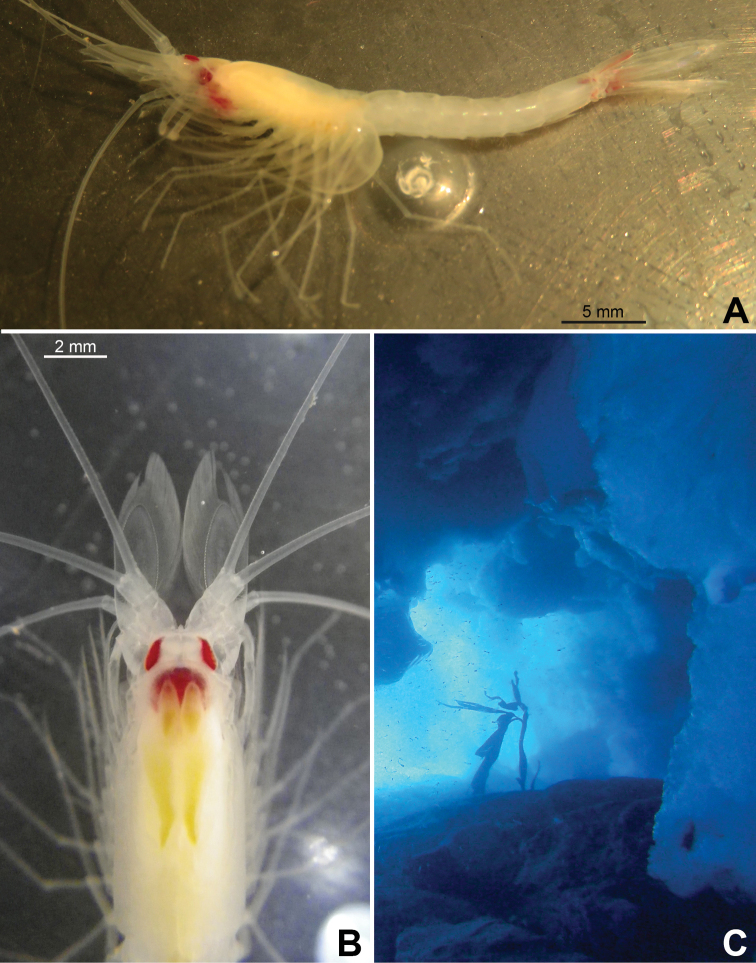
*Pseudommakryotroglodytum* sp. nov. from ice cave of Bernard Island, Antarctica **A** female holotype, lateral **B** cephalothorax of female holotype, dorsal **C** physical aspect of the ice cave environment **A, B** living specimen in laboratory.

Figures 1, 2C, 7D, 14C

The ice cave habitat, referred to in the present work, is related to the occurrence of fast ice, i.e. sea ice attached to the rocky shore, in areas where multiple islands and islets favour the persistence of such fast ice. In bridging islands, islets or even submerged rocks, sea ice therefore creates, for some time, a thick ceiling (with no or low light penetration) sustained by submerged rocky walls, themselves often covered with ice. Each habitat unit is likely to be ephemeral, some probably disappearing each summer with the ice breakup, some others persisting for years. Nonetheless, the build-up and occurrence of ice caves along rocky shores, such as the DDU area (Fig. 1) is certainly a common, recurrent process. When the width between the rocky walls is large, the ice cave will be opened at both ends, providing shelter from light, but allowing for significant water movement through the resulting tunnel shape. This was the case at Damiers and Curie Islands. In contrast, when the geomorphological context leads to a narrower passage between rocky walls, then one extremity of the ice cave might be sealed with ice. This can lead to a much more pronounced darkness and negligible water movement. Accordingly, ice caves are likely a common habitat near the Antarctic shore, but are not permanent. Recolonisation by the local fauna must, therefore, be possible, including occasionally typical deep-water species that find compatible environmental conditions there. To our knowledge, this habitat has never been described before.

We, therefore, explored, and sampled two types of ice caves. At Damiers and Curie Islands, ice caves were large and widely opened at both ends upon inspection in January–February 2018. They were typically 6–10 m wide, 15–20 m long and 4–6 m high (Fig. 14C). Strong water circulation was observed by the divers. Darkness was not absolute, except in small recesses. Mobile fauna in contact with the icy walls comprised teleost fish, polynoid worms and amphipods, either observed on the ice or within small holes in the ice. *Mysideteshanseni* Zimmer, 1914, observed in such ice caves, were very often isolated, immobile individuals (Figs 14A, B) in contact with the ice.

A second type of ice cave was observed at Bernard Island in January 2016 (it had disappeared by January 2018), in the form of two 10–15 m long icy corridors (1.5–2 m in diameter; Fig. 7D) leading to a dead end. These caves were almost entirely covered by ice (Fig. 2C) and comprised totally dark areas occupied by large numbers of young nothotheniid fish *Pagotheniaborchgrevinki* (Boulenger, 1902). Other fish, as well as polynoids including the large-sized *Eulagiscauschakovi* (Pettibone, 1997) and amphipods, were also observed on or sometimes in the ice, as if trapped. *Mysidetesilligi* Zimmer, 1914, was common and abundant, observed in small groups hovering over the ice (Fig. 7C), while *Pseudommakryotroglodytum* sp. nov. was found in contact with the icy substrate.

### ﻿Systematics

#### Order Mysida Boas, 1883

**Family Mysidae Haworth, 1825**

**Subfamily Erythropinae Hansen, 1910**

#### Tribe Pseudommini Wittmann, Ariani & Lagardère, 2014

##### ﻿Genus Pseudomma

Taxon classificationAnimaliaMysidaMysidae

G.O. Sars, 1870

BE91362E-A856-5F59-96E7-09F88866FB94

Pseudomma G.O. Sars, 1870a: 154–155 (new genus, description of type species); G.O. Sars 1870b: 48–53, Fig.-Tab. 4 (description, diagnosis); Czerniavsky 1887: 12 (key to species); Stephensen 1910: 128–133 (diagnosis, in key to genera); Tattersall and Tattersall 1951: 230–232 (diagnosis, description); Murano 1974: 288–331 (revision, key to species); Meland and Brattegard 1995: 108–143 (revision, key to North Atlantic species); Meland 2004: 1–19, Figs 1–4 (species diversity, phylogeny); Meland and Willassen 2004: 544, Fig. 4 (phylogeny, biogeography); Petryashov 2006: 1411, 1419 (Antarctic records; in key to species); Meland and Brattegard 2007: 44, Figs 3–8 (taxonomy; key to species); San Vicente 2011a: 48, Tab. 4 (list, diagnosis and key to Antarctic species); Petryashov 2014: 149 (Antarctic biogeography); Wittmann et al. 2014: 337 (taxonomy, species numbers); San Vicente 2017: Tabs. 1, 2 (geographical and bathymetric distribution); Mees and Meland 2021: AphiaID 119900 (taxon accepted).

###### Diagnosis.

Pseudommini with eyes reduced to single eyeplate without visual elements. Eyeplate with incomplete disto-median fissure. Appendix masculina large, setose. Lateral margin of antennal scale with smooth basal portion ending in a tooth. Carapace normal, with rounded anterior margin, dorsally covering at least five thoracic somites. Labrum, as far as known, with rounded rostral margin. Thoracic endopods essentially normal, non-prehensile, endopods 3–8 long and slender. Two or three pairs of oostegites contribute to marsupium wall, the two posterior pairs, as far as known, with setae on inner, as well as outer faces. Penes, as far as known, long and slender. Male pleopods with distinct sympod bearing separate, setose endopod and exopod. Female pleopods fused to small, setose, undivided plates with residual differentiation of the endopod as a pseudobranchial lobe. Endopod and exopod of uropods unsegmented, setose all around; endopod with or without spine. Telson mostly trapezoid, also linguiform or subtriangular, no cleft. Its lateral margins entirely smooth or proximally smooth with spines only along distal portions; terminal margin with spines, in several species also with medio-apical pair of setae.

###### Species inventory.

Type species is *Pseudommaroseum* G.O. Sars, 1870. World-wide, 46 species, including the new one, are here acknowledged, whereby *P.oculospinum* W.M. Tattersall, 1951 is included according to Wittmann et al. (2014). San Vicente (2011b) listed eleven species in his key to Antarctic and sub-Antarctic species. Twelve species, including the new one, are here acknowledged for waters of the Southern Ocean as in the following key:

### ﻿Key to Southern Ocean species of *Pseudomma*

**Table:** 

1	Lateral margins of the telson with one or more spines at more than 10% distance from disto-lateral edge	**5**
–	Lateral margins of telson without spines, not considering potential 1–3 spines on disto-lateral edge	**2**
2	Telson with well-rounded, convex terminal margin	**4**
–	Telson with transversely truncate terminal margin	**3**
3	Apical lobe less than 1/10 length of antennal scale	***P.antarcticum* Zimmer, 1914** (South Shetlands Islands, Antarctic Peninsula, Weddell Sea and East Antarctica, 63°N–80°S, depth 278–3425 m; San Vicente 2011a; S#13); in the Iceland Basin (N-Atlantic) in 1800–2300 m depth; Meland and Brattegard 2007).
–	Apical lobe 1/4 length of antennal scale	***P.kryotroglodytum* sp. nov.** (marine ice cave at Bernard Island (Adélie Land, East Antarctica), 67°S, 140°E, depth 10 m; S#1–2).
4	Terminal margin of telson armed with five pairs of smooth spines increasing in length distally	***P.melandi* San Vicente, 2011** (Bellingshausen Sea, 70°S, 86°W, depth 1395 m; San Vicente 2011b).
–	Terminal margin of telson armed with eight pairs of spines increasing in length distally, whereby the large, apical-most spines appear hispid due to minute scales	***P.bellingshausensis* San Vicente, 2011** (Bellingshausen Sea, 70°S, 85°W, depth 612 m (San Vicente 2011b)).
5	Lateral margins of telson with spines arranged in series of long spines with smaller spines in between; telson length thrice maximum width	***P.longicaudum* O.S. Tattersall, 1955** (Schollaert Channel (Antarctic Peninsula), 63°S, depth 160–336 m; Meland 2004, San Vicente 2011a).
–	Lateral margins of telson with small spines only, or with spines continuously increasing in length distally; telson length 1–3 times maximum width	**6**
6	Apical lobe exceeds half-length of antennal scale; telson triangular, short, about as long as its maximum width near basis	***P.minutum* O.S. Tattersall, 1955** (Puerto Montt Bay, Beagle Channel, Falkland Islands (Malvinas), 43°S–56°S, depth 30–278 m; Brandt et al. 1999, Meland 2004, Petryashov 2006).
–	Apical lobe shorter than 1/3 length of antennal scale; telson linguiform to trapezoid, longer than maximum width near basis	**7**
7	Apical lobe not exceeding 1/7 length of antennal scale	**9**
–	Apical lobe about 1/5 length of antennal scale	**8**
8	Telson length 1.4–1.5 times width near basis, lateral margins with 5–6 small spines; terminal margin convex, continuously rounded, with three pairs of large spines	***P.calmani* O.S. Tattersall, 1955** (Puerto Montt Bay, Falkland Islands (Malvinas), South Georgia, Weddell Sea, 43°S–73°S, depth 94–390 m; Meland 2004; Petryashov 2006, 2014; S#14).
–	Telson length 1.7–2.0 times width near basis, lateral margins with 7–8 small spines; telson obtuse-angled triangular at apex, almost transversely truncate; terminal margin with 3–5 pairs of large spines	***P.sarsii* Willemoës-Suhm [in G.O. Sars, 1884**] (Patagonian Shelf, Beagle Channel, Falkland Islands (Malvinas), Kerguelen Islands, South Georgia, South Orkneys, South Shetlands, Bransfield Strait, Weddell Sea, 35°N–65°S, depth 75–3962 m; Brandt et al. 1999, Meland 2004, San Vicente 2011a; S#17). **Nomenclatorial note**: In the recent past, the species name was used with ending ‘*ii*’ (Petryashov 2007: Tab. 2) and, alternatively, with ‘*i*’ (Mees and Meland 2021: AphiaID 226910). The original taxon name established by Willemoës-Suhm in G.O. Sars (1884: 37) is *PseudommaSarsii*. Therefore, the ending ‘*ii*’ is to be maintained according to the Code, Art. 33.4 (ICZN, 1999).
9	Antennal scale slender, five times longer than maximum width; telson length exceeds twice its maximum width near basis; telson with five pairs of long spines on terminal margin	***P.schollaertensis* O.S. Tattersall, 1955** (Schollaert Channel (Antarctic Peninsula), 64°S, depth 160–355 m; Meland 2004, San Vicente 2011a).
–	Antennal scale 3–4 times longer than maximum width; telson length less than twice maximum width near basis; telson with 2–3 pairs of long spines on terminal margin	**10**
10	Telson with 8–10 small spines along distal 60–70% of each lateral margin and three pairs of long spines on terminal margin	***P.magellanensi*s O.S. Tattersall, 1955** (Magellan Strait, Beagle Channel, 54–55°S, depth 50–580 m; Brandt et al. 1999, Meland 2004, Petryashov 2006).
–	Telson with 6–7 small spines along distal 40–60% of each lateral margin and 2–3 pairs of long spines on terminal margin	**11**
11	Telson with 6–7 small spines along distal 40–50% of each lateral margin and two pairs of long spines on terminal margin	***P.armatum* Hansen, 1913** (South Georgia, South Orkneys, South Shetland Islands, Weddell Sea, East Antarctica, Ross Sea, 53°S–75°S, depth 60–350 m; Meland 2004, San Vicente 2011a).
–	Telson with seven small spines along distal 50–60% of each lateral margin and three pairs of long spines on terminal margin	***P.belgicae* Hansen [in Holt & Tattersall, 1906**] (circum-Antarctic in 60°S–80°S, depth 150–1000 m; San Vicente 2011a).

#### ﻿ Pseudomma kryotroglodytum sp. nov.

Taxon classificationAnimaliaMysidaMysidae

CD9C61C1-BD69-5761-9317-85C0ACCAE211

http://zoobank.org/5B212BFB-7ADE-41CE-A671-CFC363B95A1C

**Figure 3.**
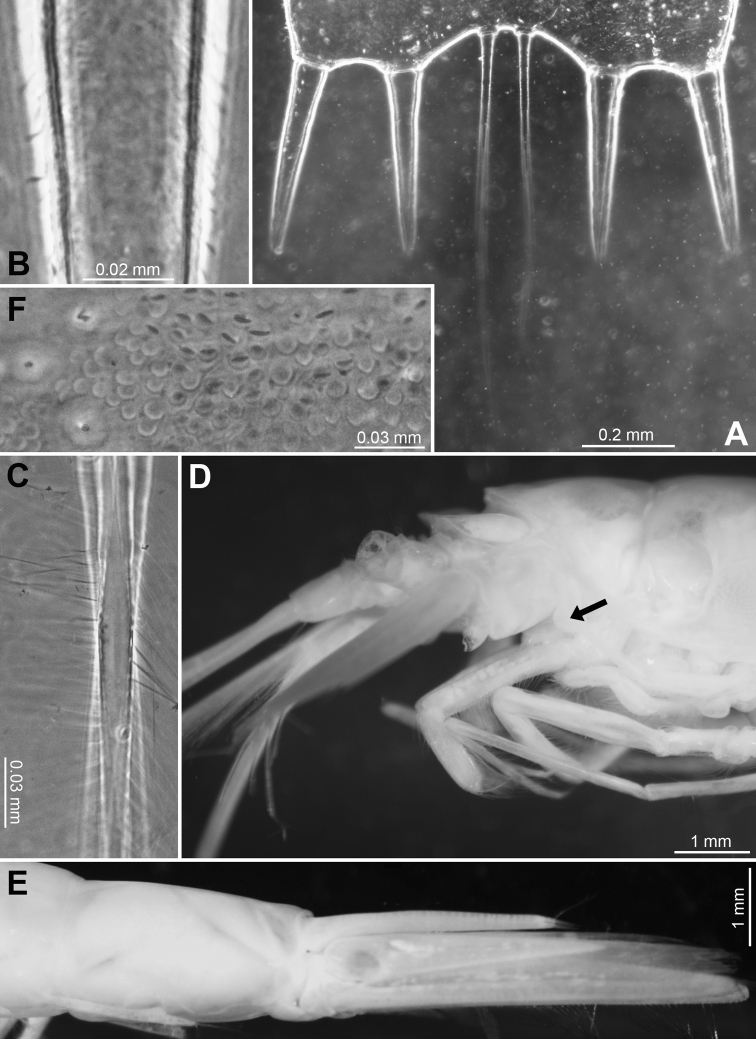
*Pseudommakryotroglodytum* sp. nov., holotype adult female BL 26.8 mm (**A–E**) and paratype subadult female 21.5 mm (**F**) **A** terminal portion of telson, dorsal, details show scales on left disto-mesial spine (**B**) and barbs on left terminal seta (**C**) **D** anterior half of cephalothorax, lateral, arrow points to distolateral edge of carapace **E** sixth pleomere with tail fan, lateral **F** example for pores (three to the left) and coat of scales on tergite of first pleomere.

**Figure 4.**
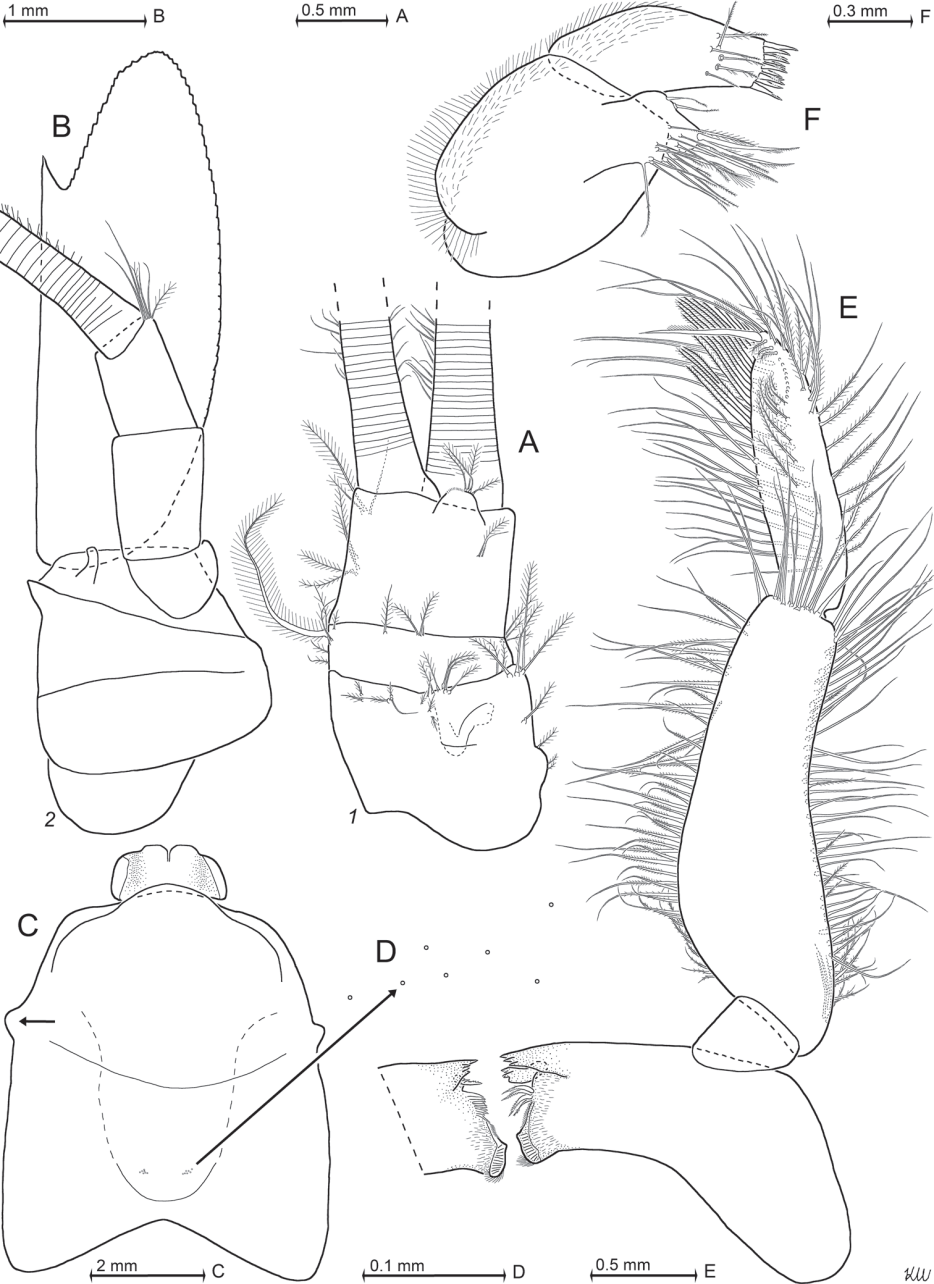
*Pseudommakryotroglodytum* sp. nov., holotype adult female BL 26.8 mm (**A, B, E, F**) and paratype subadult female 21.5 mm (**C, D**). **A** right antennula, dorsal **B** antenna with antennal gland, ventral, setae omitted from antennal scale **C** eyeplate and carapace expanded on slide, short arrow points to distolateral edge of carapace, detail (**D**) pore group in pre-cardial position **E** mandibles with left palpus, caudal aspect **F** maxillula, caudal. Scales omitted from objects **A–C, E, F** but not from eyeplate in panel **C**; pore diameters not to scale in **C, D**.

**Figure 5.**
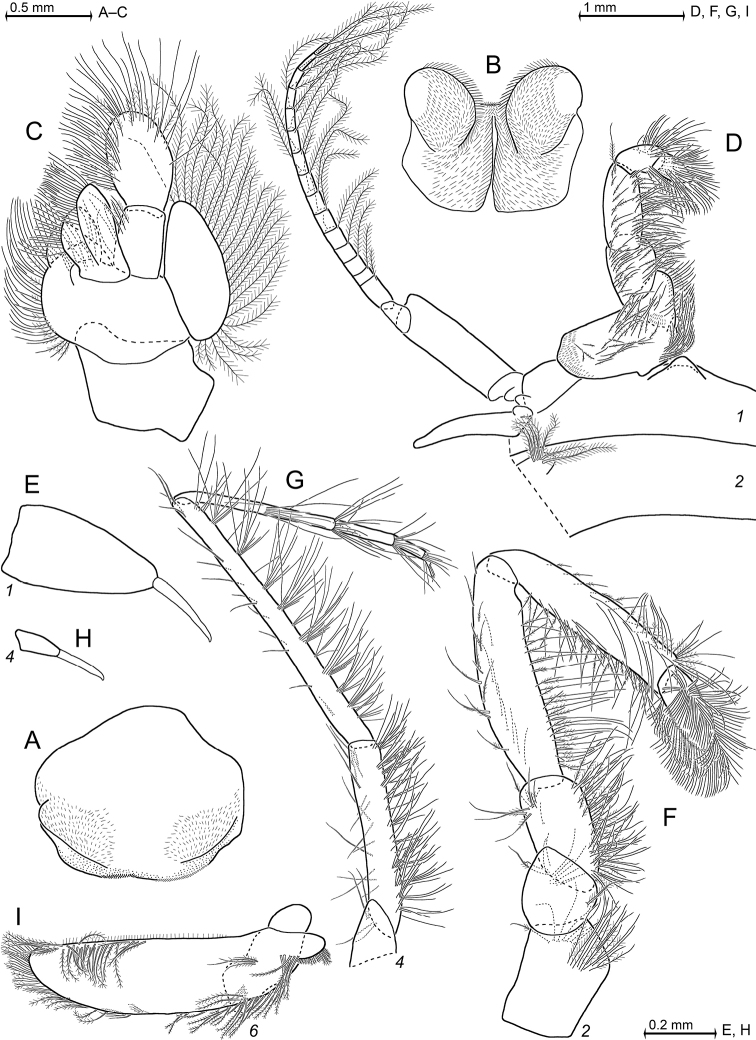
*Pseudommakryotroglodytum* sp. nov., holotype adult female BL 26.8 mm **A** labrum **B** labium **C** maxilla, caudal aspect **D** thoracopod 1 (caudal) with thoracic sternites 1, 2 (ventral) **E** detail of panel (**D**) showing dactylus 1 with nail **F** thoracic endopod 2, rostral **G** thoracic endopod 4, rostral **H** detail of panel (**G**) showing dactylus 4 with nail **I** inner face of oostegite 1, drawn above part of sympod 6. Scales omitted from objects **A–D, F, G**; setae from **E, H**.

**Figure 6.**
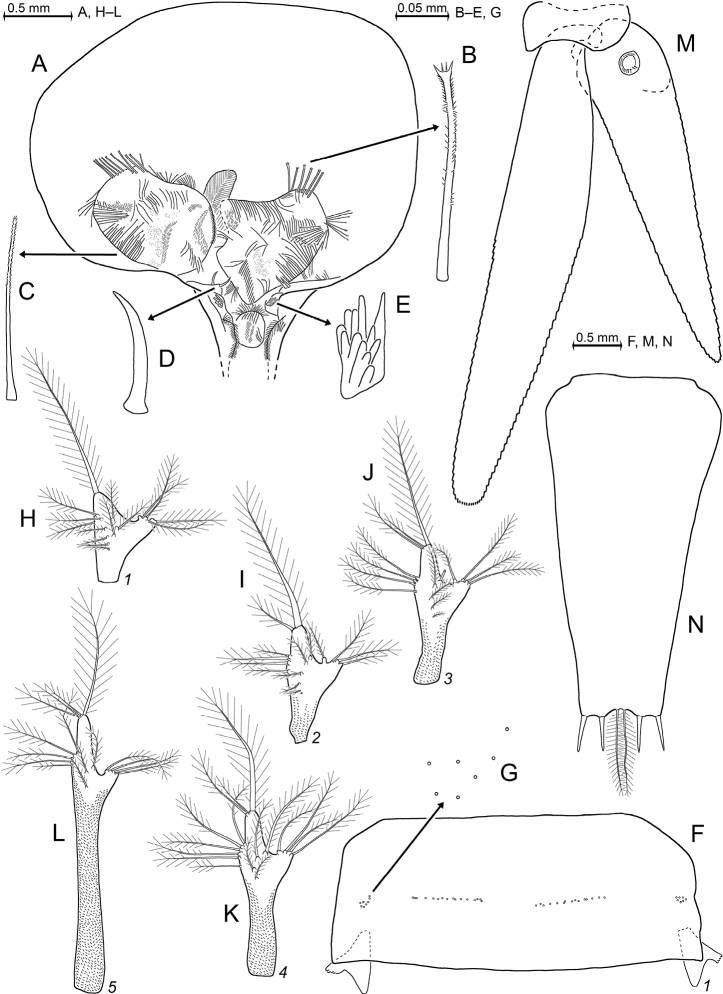
*Pseudommakryotroglodytum* sp. nov., holotype adult female BL 26.8 mm (**A–E, H–N**) and paratype subadult female 21.5 mm (**F, G**). **A** cardiac portion of foregut, dorsal view, dorsal wall omitted **B–D** details of panel (**A**) showing modified spines **E** details of panel (**A**) showing teeth emerging from a common basis **F** pleonite 1, cuticle detached and expanded on slide, dorsal and lateral faces on top, ventral face folded in, setae omitted from pleopods **G** detail of panel (**F**) showing pore group on left lateral face **H–L** series of pleopods 1–5, rostral = lateral face **M** uropods, ventral **N** telson. Scales omitted from objects **F, M, N** pore diameters not to scale in **F, G**.

Figures 2 , 3 , 4 , 5 , 6 , 23A, B, D , Table 1 , Suppl. material 1

##### Type series.

***Holotype*** spent female (♀0–) BL 26.8 mm (on slides at NHMW 27296, GenBank nos. OK351330 and OK353694), East Antarctica, Adélie Land, near Dumont d’Urville Station, NE of **Claude Bernard Island**, 66°39.64'S, 140°01.55'E, ice cave, dive #612, depth 10 m, diver-operated suction bottle, 15 Jan 2016, leg. P. Chevaldonné & S. Hourdez. ***Paratype*** subadult female (♀S–) BL 21.5 mm (on slides at NHMW 27297, GenBank nos. OK351329 and OK353693), dive #611, 13 Jan 2016, remaining sampling data as for holotype.

##### Diagnosis.

Covers females only. Species of the genus *Pseudomma* G.O. Sars, 1870, with cornea-like lateral portions separated by sulci from main part of eyeplate (Figs 2B, 4C, 23A), no visual elements. Disto-median fissure penetrates one third of eyeplate. Distal margin of eyeplates with series of minute teeth along sublateral sector (‘shoulders’, Fig. 23A, D). Basal segment of antennular trunk without medio-ventral carina. Antennal scale (Fig. 4B) with setose apical lobe contributing about 1/4 scale length. Mandibular palp (Fig. 4E) 3-segmented, very large, about as long as antennal scale. Three pairs of oostegites (Fig. 5I) contributing to wall of brood pouch. Pleopods (Fig. 6H–L) reduced to setose rods with residual differentiation of endopod (pseudobranchial lobes). Telson (Figs 3A, 6N) trapezoid, as long as ultimate pleonite. Its length twice maximum width at basis and four times width at apex. Lateral margins of telson without setae and spines, only minute scales present. Transversely truncate terminal margin with only two pairs of spines, both hispid due to minute scales (Fig. 3B) along more than proximal 2/3 spine length. Large latero-apical and same-sized submedio-apical spines flank median pair of closely set setae (Fig. 3C) with twice spine length. Margin with short, well-rounded indentation between each spine, median indentation largest. Disto-lateral edge without tooth, with spine only.

##### Description of the holotype.

All features of the diagnosis. Female with body length 26.8 mm. Cephalothorax measures 39% body length, pleon without telson 48%, telson 13% and carapace 32%. Large parts of the body, particularly carapace, pleon, telson, and uropods scaly-hispid; most appendages and eyeplates only to a minor degree. However, with 30× episcopic microscopy, the entire body appears smooth (Fig. 3D–E) due to small size of scales. With 600× transmitted phase contrast microscopy, large areas of the (artificially shed) dorsal cuticle of the animal resembles fish skin (Fig. 3F) due to dense scale cover. Ventral portions of pleomeres less densely covered, thoracic sternites smooth.

***Antennula*** (Figs 2B, 4A). Epi-antennular process triangular, projecting in median position beyond eyeplate like a small arrowhead (Fig. 2B). Antennular trunk with three sparsely setose segments, separated by transverse articulations. Basal segment 45%, median segment 16% and terminal segment 39% trunk length. Length of basal segment is only 2/3 width; mid-dorsally with deep antennular depression leading down to a striated pad at the bottom (Figs 4A, 24A, B) as described below. Basal segment not produced at outer distal corner. Terminal segment with the usual dorsal lobe on distal margin. This lobe without spiniform extension, disto-laterally with four barbed setae, mid-terminally and disto-medially with thickened, rugged margin. Flagella large, width of outer flagellum measured near basis with 1.1–1.2 times width of inner flagellum. Trunk with scales over major portions of its surface, not so the flagella.

***Antenna*** (Fig. 4B). Antennal scale large, 1.8 times length of antennular trunk and 1.8 antennal peduncle. Scale extends 0.4 times its length beyond antennular trunk and 0.7 beyond eyeplate (taking into account that antennulae insert more rostrally). Scale unsegmented, 2.9 times longer than wide. Scale dorsally and ventrally scaly-hispid all over. The smooth portion (not considering minute cuticle scales) of its outer margin ends in a strong tooth; setose apical lobe extends 26–27% scale length beyond this tooth. Antennal peduncle three-segmented. Basal segment contributes 21%, median segment 42% and terminal segment 36% peduncle length. Sympod angular on disto-lateral edge, not forming a tooth-like projection. Sympod with hispid lateral face.

***Eyes*** (Figs 2B, 3D, 4C, 23A, B, D). Eyeplate extending 0.9 times the length of terminal segment of antennular trunk along mid-line beyond anterior margin of carapace. Length of eyeplate, including its dorsally covered portion, 1.3 times the length of terminal segment. In dorsal view, superimposed dorsal and ventral sulci separate cornea-like lateral portions from main part of eyeplate (Figs 2B, 4C, 23A). Eyeplate containing tear-shaped cyst narrowing distally up to conjunction with eyeplate cleft (Fig. 23B). Sub-lateral portions of dorsal face with cover of minute scales (as in Fig. 3F; visualised with 400× microscopy), series of 15 minute teeth (not all in focus in Fig. 23D), closely set along anterior margin of this portion. Brilliant red cornea-like portions of eyeplate feign functional eyes in living specimens (Fig. 2B). Eyeplates become transparent (Figs 4C, 23A) after expansion on slide, embedding in Swan-medium and resultant bleaching; neither functional nor both vestigial ommatidia and neuronal structures visible.

***Carapace*** (Figs 3D, 4C) with broadly rounded anterior margin, disto-lateral edges well rounded. No typical rostral plate present, but a frontal bulge dorsally covered by the carapace; bulge best seen in lateral view (Fig. 3D). Antero-lateral edge of carapace with rounded protrusion (marked by arrows in Figs 3D, 4C). By forcing the detached carapace in a plane (Fig. 4C), this protrusion becoming shifted caudally compared with its position *in situ* (Fig. 3D). Carapace with cervical sulcus and cardial sulcus distinct; posterior margin concave, terminal indentation widely triangular. Two submedian groups of 8–10 pores symmetrically arranged directly in front of cardial sulcus (Fig. 4D shows only seven pores in the smaller paratype). Carapace leaving posterior 1.5 thoracomeres dorsally exposed.

***Mouthparts*** (Figs 4E, F, 5A–C). Labrum normal (Fig. 5A), rostrally forming a broad, rounded bulge; most caudal portions with strong lamellae and cover of scale-like fringes. Basal segment of mandibular palp (Fig. 4E) contributing 9–10%, median segment 55–56% and apical segment 35–37% to total palp length. Length of median segment 3.1–3.2 times maximum width; its mesial margin convex, lateral margin sigmoid. Length of apical segment 3.7–3.8 times maximum width. Palp not hispid, its basal segment without setae, remaining segments densely setose along mesial and lateral margins. Caudal face of median segment with dense field of fine hairs near basis. Masticatory part of mandibles strong, asymmetrical. Left mandible as normal in Mysidae. Pars incisiva of the new species with three large teeth and digitus mobilis with four strong teeth. Spine row with four spines ‘serrated’ by numerous stiff bristles; processus molaris with grinding lamellae not ending in teeth and with dense cover of stiff bristles. Right mandible as normal in the genus *Pseudomma*; in the new species with four large teeth on pars incisiva; digitus mobilis with only one large apical tooth serrated by secondary teeth. Right spine row present as series of nine medium-sized smooth teeth plus a few small ones, rather than a smaller number of ‘serrated’ spines present on the left mandible as otherwise usual for both mandibles in Mysidae. Right processus molaris with strong masticatory lamellae, each with small, tooth-like, apical projection; processus with cover of stiff bristles less dense than that of left mandible.

Paired labia (Fig. 5B) with stiff setae, lacking spines or teeth. Distal segment of maxillula (Fig. 4F) with 11–12 weakly serrated, strong spines on terminally truncate margin; subterminally with 5–6 barbed setae. Holotype with 5–9 pores on the surface between setae bases and spines; potential additional pores may be covered by these setae (no pores identified in the paratype). Endite of maxillula with numerous normal setae; distally with three large, modified setae, armed with stiff bristles near apex¸ more proximally with a shorter seta bearing an apical brush of long bristles.

***Maxilla*** (Fig. 5C) with well-developed exopod, two-segmented endopod (palp) and four setose endites. Exopod normal-sized, shortly extending beyond basal segment of endopod. Outer margin and apex of exopod with dense series of plumose setae distally increasing in size. Length of apical segment of endopod 1.5–1.6 times its maximum width and 1.2–1.3 times length of the basal segment. Basal segment with total of eight barbed setae, namely 3–4 on caudal and 4–5 on rostral face. Apical segment with setae all around, except for proximal third of lateral margin.

***Foregut*** (Fig. 6A–E). Lateralia, infoldings and superomedianum of the cardiac chamber densely covered by smooth, slender setae and spines. Lateralia anteriorly with dense series of slender, apically coronate spines (Fig. 6B) of different length, more caudally with slender acute spines (Fig. 6C). The latter spines with minute teeth on and close to apex. Both coronate and acute spines hispid due to minute scales along distal 50–70% of shaft. Posterior part of lateralia with powerful complex of many blunt teeth arising from common base. Twelve teeth differentiated (Fig. 6E) with translucent microscopy, additional teeth not excluded. Dorsolateral infoldings with two smooth, bent spines (Fig. 6D, subapically slightly serrate only in paratype). About 2/3 of comparatively large storage volume of foregut contained masticated, unidentifiable organic materials and mineral particles, also observed in paratype.

***Thorax*** (Figs 2A, B, 5D–I). At least tergites 6–8 covered by minute scales, no pores. Sternites 1–8 without pores, scales and also without ventrally projecting median processes (Fig. 5D). Sternite 1 with short anterior lobe projecting between left and right, first thoracic endopods. Basal plates of thoracic exopods (3–4) times longer than wide (Fig. 5D), not widening distally or only minimally so; lateral portions scaly-hispid, mesially smooth; disto-lateral edge unevenly rounded. Exopods 1, 8 with 15-segmented (Fig. 5D), remaining exopods with (17–20)-segmented, setose flagellum. Basis of endopod 1 with setose endite (below drawing plane in Fig. 5D), remaining segments without clear endite. Endopods 1, 2 with six segments (Fig. 5D, F), remaining endopods with eight segments counting from basis to dactylus (basis omitted in Fig. 5G). Endopods 3–8 long and slender; length and slenderness increase caudally; ischium shows the strongest relative increase (Fig. 2A). All endopods with hispid carpopropodus and dactylus, endopods 2–8 in addition with hispid merus, to a minor degree, if any, also ischium. Carpopropodus 3–8 three-segmented; more than half its length contributed by basal segment. Setae patterns might feign additional subdivision of carpopropodus (Fig. 5G). Suture between median and terminal segment transverse in carpopropodus 3, weakly (Fig. 5G), but not always distinctly oblique in carpopropodus 4, weakly and distinctly oblique in carpopropodites 5–8. Dactylus 3–8 small. Dactylus 1, 3–8 with short, smooth claw (Fig. 5E, H). No claw detected in dense jungle of setae on dactylus 2.

***Marsupium*** (Fig. 5I) empty in this specimen. Basal to median portions of dorsal margin without setae in oostegite 1, without setae from basal to subapical portions in oostegites 2 and 3. A narrow ‘fur’ of densely set tiny hairs forming a ribbon along dorsal margin of oostegite 1 (Fig. 5I), no such hairs in oostegites 2 and 3. Scales on large portions of outer face in oostegites 2 and 3; no scales seen on oostegite 1. Ventral and anterior margins plus part of posterior margin with dense series of barbed setae, together with bilaterally opposite oostegite forming gate contributing to the ventral and caudal closure of marsupium (this configuration not impeding respiratory water flow through marsupium). Numbers of barbs per seta increase distally, albeit not reaching those of typical plumose seta. Oostegite 1 with barbed setae also on distal half of dorsal margin, suggesting pervious anterior closure of marsupium. Posterior parts of oostegites 1–3 on inner face with comparatively long setae microserrated on their distal half. Outer face of oostegites 2 and 3 with slender whip setae characterised by barbed shaft bearing longer thin flagellum. These setae implanted on distal portions of oostegites 2 and 3; additional whip setae along ventral margin only in oostegite 3. No whip setae in oostegite 1.

***Pleon*** (Figs 3E, F, 6F–J). Pleonites 1–5 are each 0.6 times length of pleonite 6 measured along dorsal mid-line. Pleopod size increases distally. Pleopods 4–5 reaching only up to 2/3 length of pleonites 5 or 6, respectively. Not considering setae, pleopod 1 smooth (Fig. 6H) all around, remaining pleopods completely smooth only on caudal face, though scaly-hispid at least near basis of rostral (outer) face. Relative coverage with scales increases in series of pleopods 2–5 (Fig. 6I–L). All six pleonites dorsally densely covered by minute scales. Only pleonite 1 showing pores, namely two symmetrical transverse linear series each with 17–18 discontinuously spaced pores, in submedian position on dorsal face; and one additional, non-linear cluster of 8–9 pores on each lateral face (Fig. 6F–G shows fewer pores for the smaller paratype). Scutellum paracaudale sinusoid, well rounded.

***Tail fan*** (Figs 3A–C, E, 6M, N). Telson, endopod and exopod of uropods 1.0, 1.1–1.3 or 1.5–1.8 times length of sixth pleonite, respectively. Uropods (Figs 3E, 6M) long, margins setose all around, dorsal and ventral faces scaly-hispid. Exopod with slightly convex, almost straight lateral margin and with more strongly convex mesial margin. Both margins diverge up to maximum width of the exopod at 1/3 length from basis and then converge up to the U-shaped, well-rounded terminus. Distal 4/5 of endopod with straight margins converging in V-shaped manner up to narrowly blunt apex. Endopod 0.7 times length of exopod, extending 1/5 of its length beyond telson, exopod 2/5 its length. Both statoliths unevenly discoid, mean diameter 0.22–0.23 mm, thickness 0.18 mm; core unevenly discoid as well, diameter 0.14 mm. Statolith formula 2 + 3 + (5–7) + 10 + (12–14) = 34. Statoliths composed of fluorite. Lateral margins and dorsal face of telson (Fig. 6N) completely covered by scales as in Fig. 3F, whereas ventral face only on its distal third.

**Colour** (Fig. 2A, B). Live colour was documented only in the laboratory; no difference visible between the two type specimens. Body and appendages generally whitish transparent. Lateral portions of eyeplate, foregut and part of mouthparts brilliant red, hepatic caeca yellow-green. The anterior pair of caeca covers part of the foregut dorsally, leaving a red M-like sign on the foregut in dorsal view. Posterior part of ultimate pleomere and adjoining basal portions of tail fan tinged light-red.

##### Etymology.

The species name is an adjective with Latinised neutral ending formed by adjectivation of the amalgamated Ancient Greek adjective κρύος (cold) with the noun τρωγλοδύτης (cave dweller). The adjectivation has precedence in the name of the butterfly *Macroglossumtroglodytus* Boisduval, l875, listed by Kemal et al. (2019) as *M.troglodytum*.

##### Type locality.

Marine ice cave NE of Claude Bernard Island, Adélie Land, East Antarctica, 66°39.64'S, 140°01.55'E, depth 10 m.

###### Subfamily Heteromysinae Norman, 1892

**Tribe Mysidetini Holt & Tattersall, 1906**

#### ﻿Genus Mysidetes

Taxon classificationAnimaliaMysidaMysidae

Holt & Tattersall, 1906

9A5C072A-F828-512D-AFE5-45592B6DF762

Mysidetes Holt & Tattersall, 1906a: 39–40 (new genus, diagnosis); Holt and Tattersall 1906b: 10 (preliminary diagnosis); Hansen 1910: 9 (taxonomy, assigned to Leptomysini); Zimmer 1914: 401–402 (taxonomy); Tattersall and Tattersall 1951: 305 (diagnosis); Bowman and Orsi 1992: 738–739 (transfer to Heteromysini); Wittmann et al. 2014: 341 (type genus of Mysidetini); Mees and Meland 2021: AphiaID 119884 (taxon accepted).
Metamysidella Illig, 1906: 210–211, fig. 17 (junior synonym); Mees and Meland 2021: AphiaID 226152 (not accepted).

##### Diagnosis.

Mysidetini with eyes well developed; cornea large, globular, with functional ommatidia; eyestalks well developed. Antennula usually without modified setae (exception: females of *M.illigi* Zimmer, 1914, as described below). Appendix masculina well-developed, setose. Antennal scale setose all around, no spines and no teeth. Mouthparts normal, maxilla without spines. Male thoracic endopod 2 without notches on outer margin. Thoracic endopods 3–8 normal, slender, not prehensile; with multi-segmented carpopropodus; small dactylus with weak claw. Penes long, slender, stiff, and not erectile. Pleopods non-dimorphic, reduced to bifid setose plates, no modified setae and no spines. Endopod of uropods usually with row of spines on inner margin (exception: *M.hanseni* Zimmer, 1914, as described below). Telson with apical cleft; cleft lined with laminae. Lateral margins of telson at least distally with spines.

##### Species inventory.

Type species is *Mysideis Farrani* Holt & Tattersall, 1905, current name *Mysidetesfarrani* (Holt & Tattersall, 1905). Total of 17 species including the here re-installed *M.illigi* Zimmer, 1914, are acknowledged as in the following key:

### ﻿World-wide key to species of *Mysidetes*

**Table:** 

1	Terminal lobes of telson narrowly truncate	**6**
–	Terminal lobes of telson rounded (convex)	**2**
2	Apical cleft penetrates 1/20 telson length, margin of cleft lined all along with 7–11 short laminae; apical lobes of telson narrowly rounded; proximal half of telson unarmed, distal half with three spines on each lateral margin; endopod of uropods with 12–13 spines	***Mysideteshalope* O’Brien, 1986** (in shallow water of a marine cave in Tasmania, 43°S, 148°E; O’Brien 1986).
–	Apical cleft penetrates 10–15% telson length, armed with 14–18 laminae; apical lobes of telson broadly rounded	**3**
3	Antennal scale more than six times as long as broad; endopod of uropod with more than thirty densely set spines	**5**
–	Antennal scale less than six times as long as broad; endopod of uropod with, if any, fewer than nine loosely-arranged spines	**4**
4	Lateral margins of telson all along armed with 45–57 spines; apical cleft penetrates 1/10 telson length; margin of cleft lined all along with 14–17 laminae; endopod of uropods without spine	***Mysideteshanseni* Zimmer, 1914** (coast of East Antarctica: in ≤ 250 m depth below sea ice at Gauss Station, 66°S, 90°E; and in 6–10 m depth in ice caves of Curie and Damiers Islands, Adélie Land, 67°S, 140°E; Zimmer 1914; S#5–10).
–	Proximal 2/5 of telson without spines; distal portion with 11–13 spines on each lateral margin; apical cleft penetrates 1/7 telson length; margin of cleft lined all along with 16–18 laminae; endopod of uropod with 7–8 spines	***Mysidetesperuana* Băcescu, 1967** (Peru Trench, 8°S, 80°W, depth 520 m; Băcescu 1967).
5	Apical cleft penetrates 1/3 telson length; proximal third of telson unarmed	***Mysidetespatagonica* O.S. Tattersall, 1955** (Beagle Channel, Magellan Strait, Falklands (Malvinas), 48°S–55°S, depth 14–300 m; Brandt et al. 1999, Price 2001).
–	Apical cleft penetrates less than 1/4 telson length; proximal half of telson unarmed	***Mysidetesanomala* O.S. Tattersall, 1955** (Magellan Strait, 53°S, depth 0–300 m; Price 2001).
6	Lateral margins of telson armed all along with spines or all along, except for an unarmed sub-basal portion; most proximal (basal) portion always armed	**12**
–	Lateral margins of telson proximally unarmed (distal portions with spines)	**7**
7	Antennal scale four times as long as broad	**10**
–	Antennal scale 2–3 times as long as broad	**8**
8	Rostrum right-angled or acute, produced beyond eyestalks (in normal orientation); antennal scale short, reaching to about half-length of terminal segment of antennular trunk	***Mysidetesbrachylepis* W.M. Tattersall, 1923** (South Georgia, Falkland Islands, South Shetland Islands, Bransfield Strait and Ross Sea, 50°S–80°S; suprabenthic in 130–525 m depth; San Vicente 2011a).
–	Rostrum not covering eyestalks; antennal scale reaches to terminal margin of antennular trunk or slightly beyond	**9**
9	Rostrum weakly acute to right-angled, about half as long as terminal segment of antennular trunk; telson cleft armed all along with laminae	***Mysideteskerguelensis* (Illig, 1906)** (South Georgia, Kerguelen Islands, Weddell Sea, 49°S–60°S; suprabenthic in 60–275 m depth; San Vicente 2011a; S#27).
–	Rostrum obtuse-angled, less than half as long as terminal segment of antennular trunk; only proximal 2/3 of telson cleft armed with laminae, distal third unarmed	***Mysidetescrassa* Hansen, 1913** (Patagonia, South Georgia, Antarctic Peninsula, Weddell Sea, 45°S–71°S; suprabenthic in 18–412 m depth; Brandt et al. 1998, Petryashov 2007, San Vicente 2011a).
10	Apical cleft penetrates up to 1/5 telson length; cleft armed with 3–6 small laminae	***Mysidetesfarrani* (Holt & Tattersall, 1905)** (North Atlantic: Ireland to Morocco, U.S. east coast; Mediterranean, 33°N–52°N; bottom-living in 235–1105 m depth; Tattersall and Tattersall 1951, Wright 1973, Price 2001).
–	Apical cleft penetrates at least 3/10 telson length; cleft armed with more than 30 small laminae	**11**
11	Cornea diameter exceeds length of combined median and terminal segment of antennular trunk; rostrum about half-length of terminal segment of antennular trunk; endopod of uropods with spines from statocyst to near apex	***Mysidetesmacrops* O.S. Tattersall, 1955** (Falklands (Malvinas), South Georgia, 50°S–53°S, depth 88–503 m; Brandt et al. 1998, Price 2001, Petryashov 2007).
–	Cornea diameter not exceeding length of combined median and terminal segment of antennular trunk; rostrum about 4/5 length of terminal segment of antennular trunk; endopod of uropods with spines from statocyst to 1/5 endopod length from apex	***Mysidetesintermedia* O.S. Tattersall, 1955** (Magellan Strait, Falklands (Malvinas), 50°S–53°S, depth 94–170 m; Brandt et al. 1998, Price 2001).
12	Antennal scale projects at least 1/5 of its length beyond antennular trunk; telson cleft mostly narrow, 1.1–2.5 times deeper than its distal width	**14**
–	Antennal scale projects less than 1/5 of its length beyond antennular trunk; telson cleft widely open, depth 0.7–1.2 times distal width	**13**
13	Rostrum obtuse, shorter than half length of terminal segment of antennular trunk; endopod of uropods with about 20 spines densely arranged in continuous series between statocyst and 1/3 endopod length from apex; each lateral margin of telson with about 29 spines	***Mysidetesdimorpha* O.S. Tattersall, 1955** (South Georgia and Antarctic Peninsula, 53°S–65°S; suprabenthic in 18–295 m depth; San Vicente 2011a).
–	Rostrum acute, exceeding 2/3 length of terminal segment of antennular trunk; endopod of uropods with about 16–17 spines loosely arranged in discontinuous series between statocyst and 1/4 endopod length from apex; each lateral margin of telson with about 44–47 spines	***Mysidetesmicrops* O.S. Tattersall, 1955** (South Georgia, Falkland Islands (Malvinas) and Antarctic Peninsula, 50°S–63°S; suprabenthic in 60–250 m depth; San Vicente 2011a).
14	Each lateral margin of telson armed all along with 30–40 spines, no unarmed stretch; telson cleft with more than 35 laminae	**16**
–	Each lateral margin of telson with total of 33–47 spines, arranged as 6–9 spines at the base, followed by an unarmed stretch, median portions with subequal spines and distal portions with discontinuous series of large spines with small spines in between; telson cleft with 23–29 laminae	**15**
15	Rostrum short, leaving the eyes completely exposed; antennal scale length eight times maximum width; each lateral margin of telson with total of 33–36 spines	***Mysidetesantarctica* O.S. Tattersall, 1965** (Ross Sea, Antarctic Peninsula (Bransfield Strait), 64°S–78°S; depth 100–123 m, below ice; Petryashov 2007).
–	Rostrum covers at least basal portions of eyestalks; antennal scale length 4–7 times maximum width; each lateral margin of telson with total of 35–47 spines	***Mysidetesilligi* Zimmer, 1914** (coast of East Antarctica: in ≤ 200 m depth below sea ice at Gauss Station, 66°S 90°E; and in 6–10 m depth in ice cave at Bernard Island, Adélie Land, 67°S 140°E; Zimmer 1914; S#2–4, 9).
16	Endopod of uropod with 12–13 spines; telson cleft with 54–60 laminae	***Mysidetesmorbihanensis* Ledoyer, 1995** (Kerguelen Islands, 47°S–49°S, depth 22–128 m; Ledoyer 1995).
–	Endopod of uropod with 26–28 spines; telson cleft with about 36 laminae	***Mysidetesposthon* Holt &Tattersall, 1906** (circum-Antarctic up to the Antarctic Divergence, also sub-Antarctic: Falkland Islands (Malvinas), South Georgia Islands, South Sandwich Islands, Scotia Sea, 49°S–78°S; suprabenthic in 15–800 m depth; Petryashov 2007, San Vicente 2011a; S#28–29).

#### ﻿ Mysidetes illigi

Taxon classificationAnimaliaMysidaMysidae

Zimmer, 1914, bona species

FBB444BD-F1A4-5E06-99F6-B834D61E895B

Figures 7 , 8 , 9 , 10 , 11 , 12 , 13 , 25A–C , Table 1 , Suppl. material 1

Mysidetes illigi Zimmer, 1914: 404–405, Figs 47–49 in Fig.-Tab. XXVI (first description).
Mysidetes Illigi , Hansen 1921: 5 (proposed validity check).
Mysidetes illigi referred to as synonym of Mysidetesposthon: W.M. Tattersall 1923: 275, 288; Illig 1930: 470, 581; Müller 1993: 164; Mees and Meland 2021: AphiaID = 451694 (unaccepted).

##### Type series.

Holotype (by monotypy) subadult female (ZMB 18284) BL 12.7 mm, in vial with ethanol, labelled “D.-Südpol.-Exp. 31.12.1902, 200 m. *Mysidetesilligi* sp. nov. Typus”. Type not explicitly defined by Zimmer (1914). In accordance with the text by Zimmer (1914), the label of the jar containing this vial indicates 21.12.1902 as the date of sampling. According to Zimmer (1914), this specimen was taken on this day together with one specimen (now lectotype) of *M.hanseni* with a vertical haul 200–0 m at the ‘Winterstation’ (= **Gauss Station**), 66°02'S, 89°38'E, coast of East Antarctica, S#9.

##### Non-types from ice caves.

Three samples (S#2–4) taken in austral summer 2015–2016 by P. Chevaldonné and S. Hourdez upon diving in an ice cave of **Bernard Island**, near Dumont d’Urville Station, Adélie Land, Antarctica, 66°39.64'S, 140°01.55'E:

One spent female (♀0–) BL 17.9 mm, 5 ♂♂S 13.2–15.0 mm, 2 ♂♂I 12.1–12.9 mm, 1 ♀S^+^ 13.9 mm, 5 ♀♀I 12.3–14.1 mm (in vials, NHMW 27298, SMF-57647, ZMB 34882, ZMH-K-60866), S#3; 1 ♀0– 18.1 mm (on slides; NHMW 27300), 5 ♀♀0– 14.2–15.9 mm, 3 ♂♂S 11.3–12.6 mm, 2 ♀♀S– 14.3–15.7 mm, 1 ♀S^+^ 15.5 mm, 7 ♀♀I (in vials, NHMW 27299, SMF-57648, ZMB 34883, ZMH-K-60867), S#4; 1 ♀0– 18.4 mm (on slides, NHMW 27301) and 1 ♀I 15.6 mm (in vial), S#2.

##### Diagnosis.

**Figure 7.**
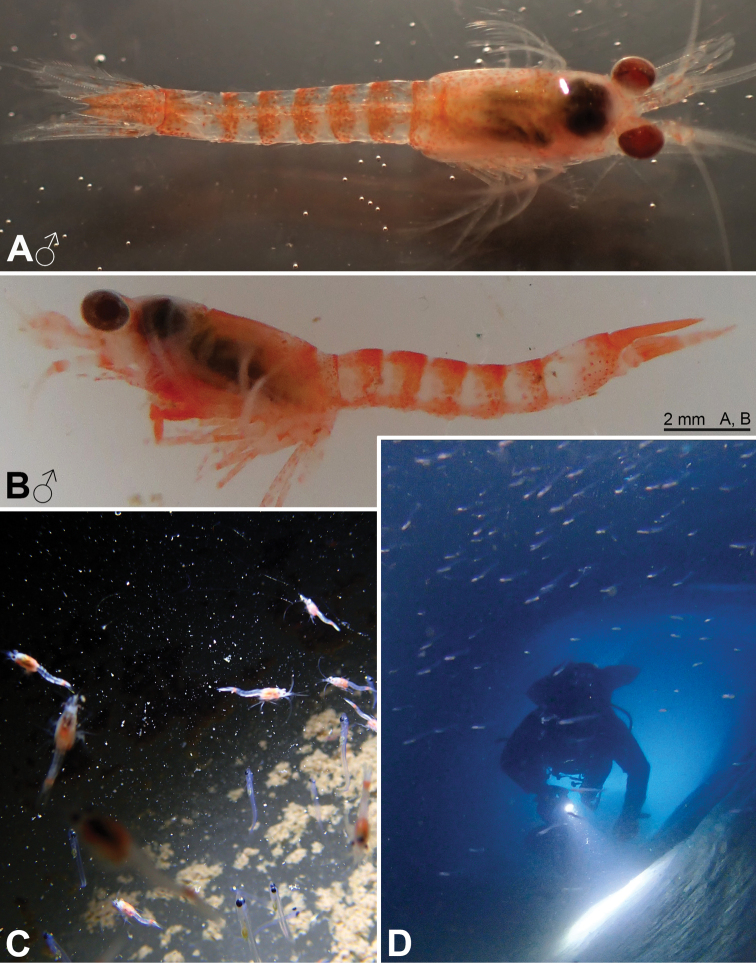
*Mysidetesilligi* from ice cave of Bernard Island, Antarctica **A** subadult male, dorsal **B** subadult male, lateral **C** hyperbenthic association formed by mysids and early stages of nothotheniid fish **D** fish swarm mixed with small number of mysids several metres inside cave **A, B**, living specimens in laboratory.

Covers adult females and subadults of both sexes:

Species of *Mysidetes* with eyes (Figs 7A, B, 9A, B) well-developed, thick. Cornea calotte-shaped, its length 0.8 times length of eyestalk, diameter 1.6–1.8 times length of terminal segment of antennular trunk. Eyestalk without ocular papilla; length 0.7–0.8 times its width at conjunction with cornea. Rostrum triangular with acute to narrowly-rounded apex and with concave, up-tilted lateral margins; rostrum 0.8–1.0 times as long as terminal segment of antennular trunk.

**Figure 8.**
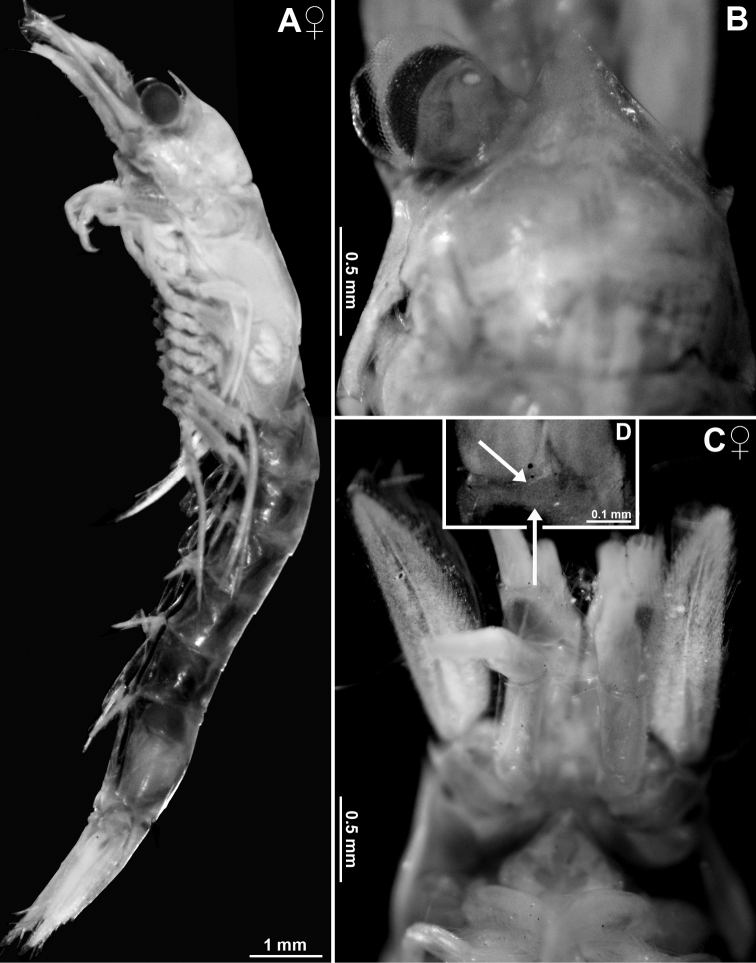
Holotype of *Mysidetesilligi*, subadult female BL 12.7 mm **A** body, lateral, most thoracic endopods broken (specimen artificially separated from background) **B** anterior body region, dorsal, cuticle lifted from cornea as fixation artefact **C** cephalic region, ventral **D** detail of (**C**) showing distal margin of right antennular trunk, arrows point to mid-dorsal lobe (female antennular lobe = derivate of appendix masculina).

***Antennae*** s.l. (Figs 8C, D, 10A–C). Terminal segment of antennular trunk with mid-ventral lobe (Figs 8D, 10B) bearing modified setae in females (Fig. 25C). Antennal sympod (Fig. 10C) with one large, acute tooth on disto-lateral edge and more caudally an additional shorter tooth. Dorsal face of sympod with triangular, apically rounded lobe. Antennal scale two-segmented, apically rounded, setose all around, with apical segment only 2% total scale length; scale 4–7 times as long as its maximum width; scale projects 0.3–0.6 times its length beyond antennular trunk.

***Mouthparts*** (Fig. 11). Median segment of mandibular palp 2.5–3.3 times as long as maximum width (Fig. 11B), densely setose all around. Right mandible with digitus mobilis and pars centralis modified as in Fig. 11B; remaining mouthparts normal, labrum not produced rostrally, maxilla without spines.

***Thorax*** (Figs 10D–K, 13A, B) without mid-sternal processes in females and non-adult males. Flagellum of thoracic exopods 1, 8 with eight segments, flagella 2–7 with nine segments (Fig. 13A). Carpopropodus of thoracic endopods 1–8 with 2, 2, 5–6, 6–8, 7–9, 6–9, 6–8 and 6–8 segments, respectively. Claw of endopod 1 (Fig. 10F) strong, subapically, unilaterally, weakly serrated; claws 3–8 (Fig. 10H–K) weak, slender, smooth. Marsupium formed by two pairs of large oostegites; additional rudimentary oostegite on thoracopod 6 (Fig. 13A). Subadult males with penes (Fig. 13B) stiff, slender, 1–2 times length of ischium of endopod 8.

***Pleon*** (Figs 8A, 13B–G). Pleopods (Fig. 13C–G) reduced to unsegmented, setose plates with comparatively large endopodal portion (pseudobranchial lobe) integrated. All pleopods without spines, no modified setae. Total length increases in series of pleopods 1 to 5.

**Figure 9.**
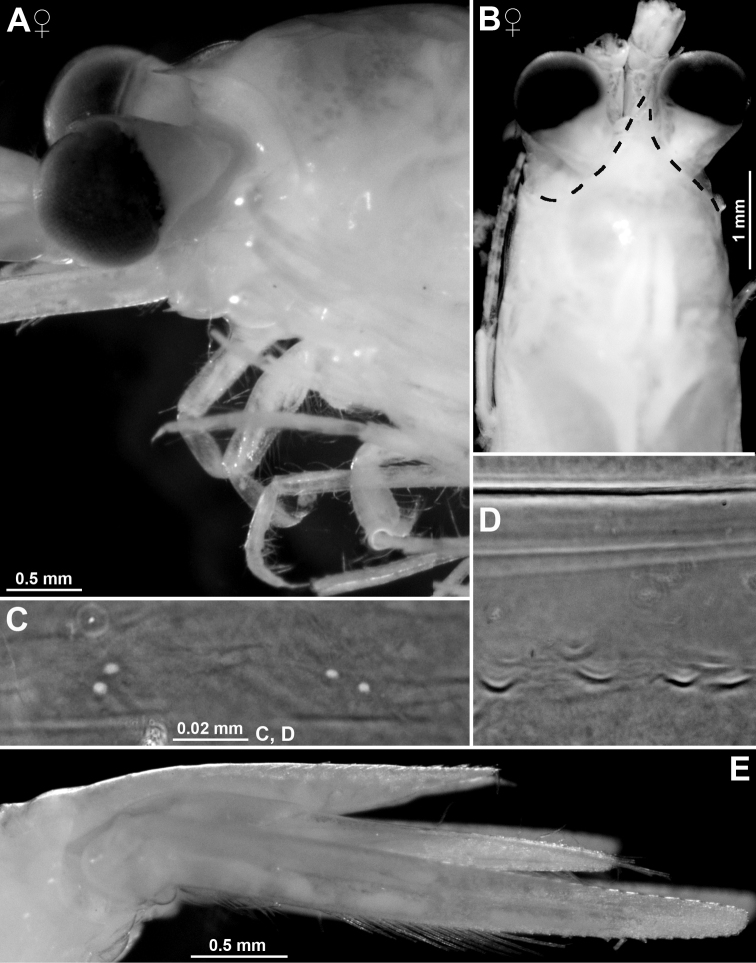
*Mysidetesilligi* from ice cave of Bernard Island, Antarctica. Adult females BL 18.1 mm (**A**, **C–E**), 18.4 mm (**B**) **A** head, lateral **B** anterior body region, dorsal, dashed line enhances the anterior contour of carapace **C** paired circular structures symmetrically arranged in front of posterior margin of carapace **D** series of cuticle structures parallel to lateral margin of carapace **E** tail fan, lateral.

***Tail fan*** (Figs 9E, 13H, I). Endopod of uropods (Fig. 13H) with 8–13 slender spines in series from statocyst to 25–35% endopod length from apex; proximal 2–4 spines short, in part crowded; remaining 6–9 spines longer, subequal amongst each other, about equally spaced in linear series. Telson (Fig. 13I) trapezoid, length 1.9–2.5 times maximum width near basis and 6–7 times minimum width on bifid terminus. Length 0.7–0.8 times exopod of uropod. Lateral margins of telson each with total of 35–47 spines; basal portions with 7–9 spines in continuous series followed by an unarmed stretch, median portions start with 2–6 spines increasing in length distally, followed up to tip by discontinuous series of large spines with small spines in between. Cleft U-shaped, penetrating 15–18% telson length, margin of cleft lined all along with 23–29 laminae of which proximal 3–4 laminae larger than remaining 20–25 subequal laminae. Cleft 2.0–2.5 times as deep as its width at apex. Disto-lateral lobes of telson triangular with narrowly truncate apex; each lobe armed with two spines at apex, mesial spine 0.5–0.7 times length of lateral spine.

##### Description of holotype.

Subadult female (Fig. 8) with 12.7 mm body length, not dissected. Status of ovarian tubes not well established. Body moderately slender, pleon (without telson) contributes 59% to total trunk length. Carapace including rostrum 32% of body length (including telson) when measured along dorsal mid-line.

**Figure 10.**
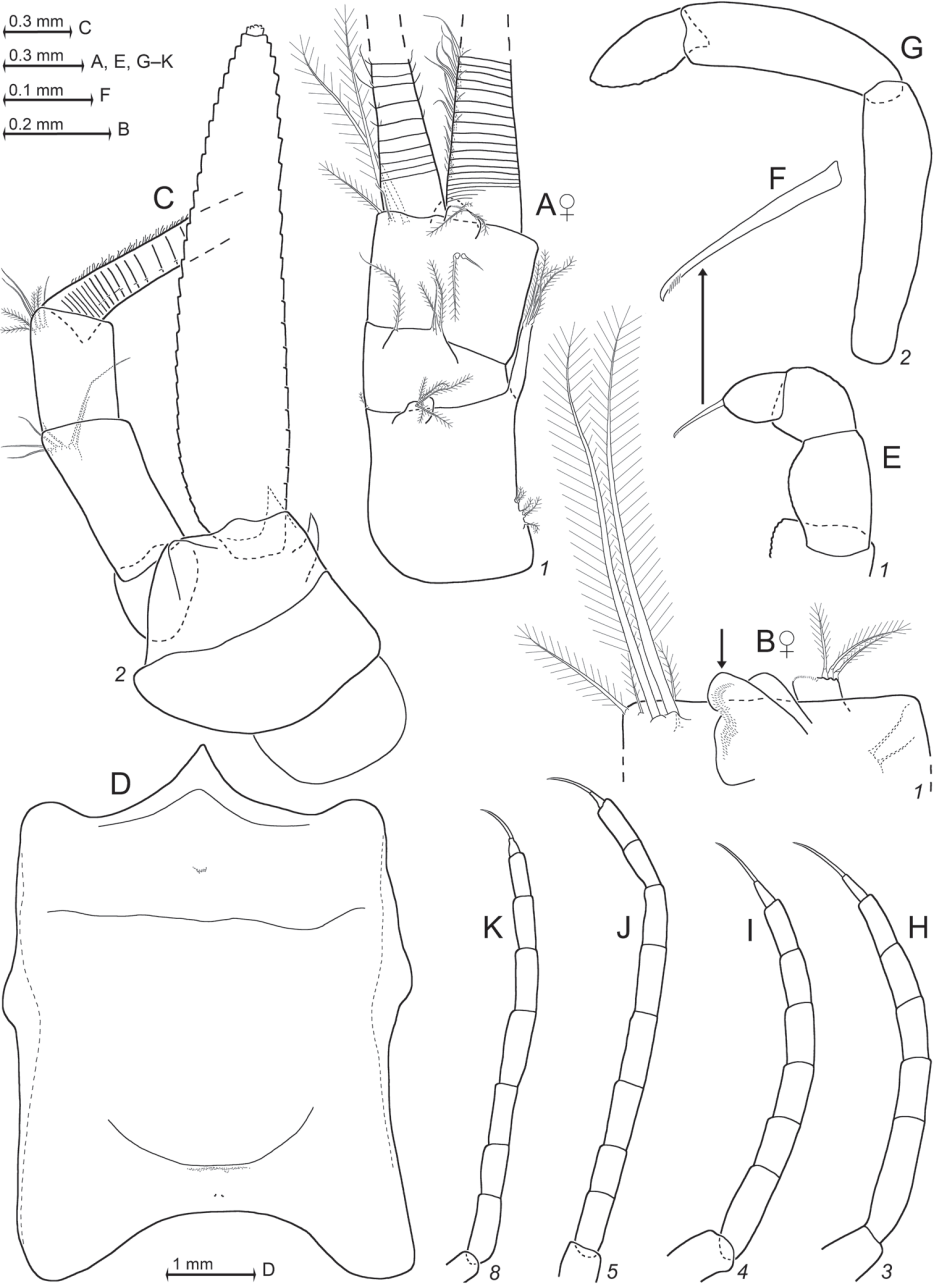
*Mysidetesilligi* from ice cave of Bernard Island, Antarctica. Adult females BL 17.9 mm (**A**) 18.1 mm (**B**, **D**, **K**) 17.3 mm (**C**), 18.4 mm (**E–J**). **A** right antennula, dorsal **B** distal margin of left antennular trunk, ventral, arrow points to mid-dorsal lobe (derivate of appendix masculina), flagellae omitted **C** antenna with antennal gland, dorsal, setae omitted from antennal scale **D** carapace expanded on slide **E** ‘tarsus’ of thoracic endopod 1, caudal, setae omitted, detail (**F**) shows claw **G–K** series of tarsus 2–5, 8, setae omitted.

***Cephalothorax*** (Fig. 8B–D). Outer lobe from basal segment of antennular trunk extending beyond median segment. Basally wide, low lobe located mid-ventrally closely behind anterior margin of terminal segment (arrows in Fig. 8D). Antennal sympod as in diagnosis (as in Fig. 10C). Scale is 3.8–4.3 times as long as its maximum width (Fig. 8C), extending 46–59% its length beyond antennular trunk and 35–47% beyond antennal peduncle. Basal segment contributing 20–24% to length of antennal peduncle, median segment 45–46%, and distal segment 30–34%. Cornea large, bulbous (damaged in this specimen). Eyestalk smooth, no ocular papilla. Frons with five horizontal bulges vertically stacked between rostrum and antennular symphysis, these ranging from subrostral process (bulge) ventrally down to that from the antennular symphysis. Rostrum (Fig. 8A, B) large, triangular, basally broad, extending to terminal margin of eyestalks or beyond, depending on orientation of eyestalks. Carapace normal, its disto-lateral edges and its caudo-lateral lobes well-rounded. Carapace leaves ultimate 1.5 thoracomeres dorsally exposed. Median segment of mandibular palp 2.5–2.7 times as long as its maximum width. Flagellum of thoracic exopods 6–7 with nine segments, flagellum 8 with eight segments; all remaining exopods and endopods 3–8 broken.

***Pleon*** (Fig. 8A). Pleonites 1–5 are 0.8, 0.7, 0.6, 0.6 and 0.5 times length of pleonite 6, respectively. Pleopods as in diagnosis (Fig. 13C–E). Exopod of uropods extends 31% its length beyond telson. Both endopods with broken tip. Slender, about equally-spaced spines along remainder of endopods; potential spines near statocyst not visible without dissection. Statolith diameter 0.27 mm.

***Telson*** trapezoid, 1.2 times length of ultimate pleonite, 1.9 times as long as maximum width near basis. Right margin of cleft lined by eleven laminae, amongst which ten distal laminae short, subequal. Bottom of cleft with three larger laminae, i.e. median lamina flanked by two submedian laminae (including the proximal one on right margin). Left disto-lateral lobe of telson distally broken. Corresponding right lobe triangular with narrowly truncate apex armed with two spines, the mesial spine 2/3 length of the lateral spine. Right lateral margin of telson almost straight, with total of 35 spines. Basal portion of both margins with 7–8 spines in continuous series, followed by unarmed stretch, median portion with 3–4 spines increasing in length distally; this series distally continued by discontinuous series of large spines with small spines in between, in the right margin up to the tip, left margin distally broken.

##### Description of adult females from ice caves.

First description of adult females; all features as given in diagnosis. General appearance moderately slender, body length 14.2–18.4 mm (n = 8). Rostrum measures 3–4% body length, thorax 33–34%, pleon 48–49%, telson 14–16% and carapace, including rostrum, 29–32%. Pleon (without telson) contributes 54–59% to trunk length. Frons with 4–5 vertically stacked, horizontal bulges, these ranging from subrostral process (bulge) ventrally down to that from antennular symphysis.

***Carapace*** (Figs 9B–D, 10D) with normal gross structure, no apparent sexual dimorphism. Rostrum covering basal portions of normally orientated eyestalks, reaching at most to distal margin of artificially straight forward-orientated eyestalks (without cornea). Carapace with disto-lateral edges and caudo-lateral lobes well-rounded. Terminal margin leaving ultimate 1.5–2.5 thoracomeres mid-dorsally exposed. Cervical sulcus strong, cardial sulcus feebly developed. Group of about 30 pores (as in Fig. 17E) about 1/9 carapace length in front of cervical sulcus and transverse series of about 40 pores (as in Fig. 17F) along cardial sulcus (Fig. 10D shows fewer pores due to limited graphical resolution). An unusual set of cuticle structures is present: two pairs of circular structures (Fig. 9C) symmetrically arranged in front of the posterior margin (Fig. 10D). Cuticle sculptured by minute depressions with crescent-shaped margins (Fig. 9D), loosely and irregularly arranged in transverse series behind fold delimiting up-tilted anterior portion of carapace, behind cardial sulcus; series also extending short distance along posterior 2/3 of lateral margins (sculptures omitted in Fig. 10D). Outer surface of carapace smooth, except for the here-described structures.

***Eyes*** (Fig. 9A, B). Eyestalks and cornea dorsoventrally not or only slightly compressed. In dorsal view, cornea appears calotte-shaped, in lateral view oviform to spherical. Stalk mesially near basis with hispid bulge, remaining (= major) portions with smooth cuticle.

***Antennulae*** (Fig. 10A, B). Trunk measures 8–9% body length extending 0.3–0.5 times its length beyond eyes and is 2.2–3.2 times longer than its maximum width. Segments 1–3 measure 46–48%, 18–19% and 34–37% length of antennular trunk, respectively. Lateral lobe from basal segment extends beyond median segment. Median segment with its mesial face not inflated. Terminal segment 0.7–0.9 times as long as wide. Its mid-dorsal apophysis with 3–4 barbed setae, with small cilia lining the disto-mesial margin; no spiniform anterior projection. Lateral antennular flagellum in adult females 1.2–1.4 times width of the mesial flagellum when measured near basis of flagella.

***Antennae*** (Fig. 10C). Sympod caudally with bulbous lobe containing end sac of antennal gland. The three-segmented peduncle with basal segment 20–23% peduncle length, second 43–46% and third 33–36%, respectively. Third segment 1.1–1.6 times as long as wide. Antennal scale with convex mesial margin; lateral margin slightly sigmoid, almost straight. Small apical segment with five plumose setae.

**Figure 11.**
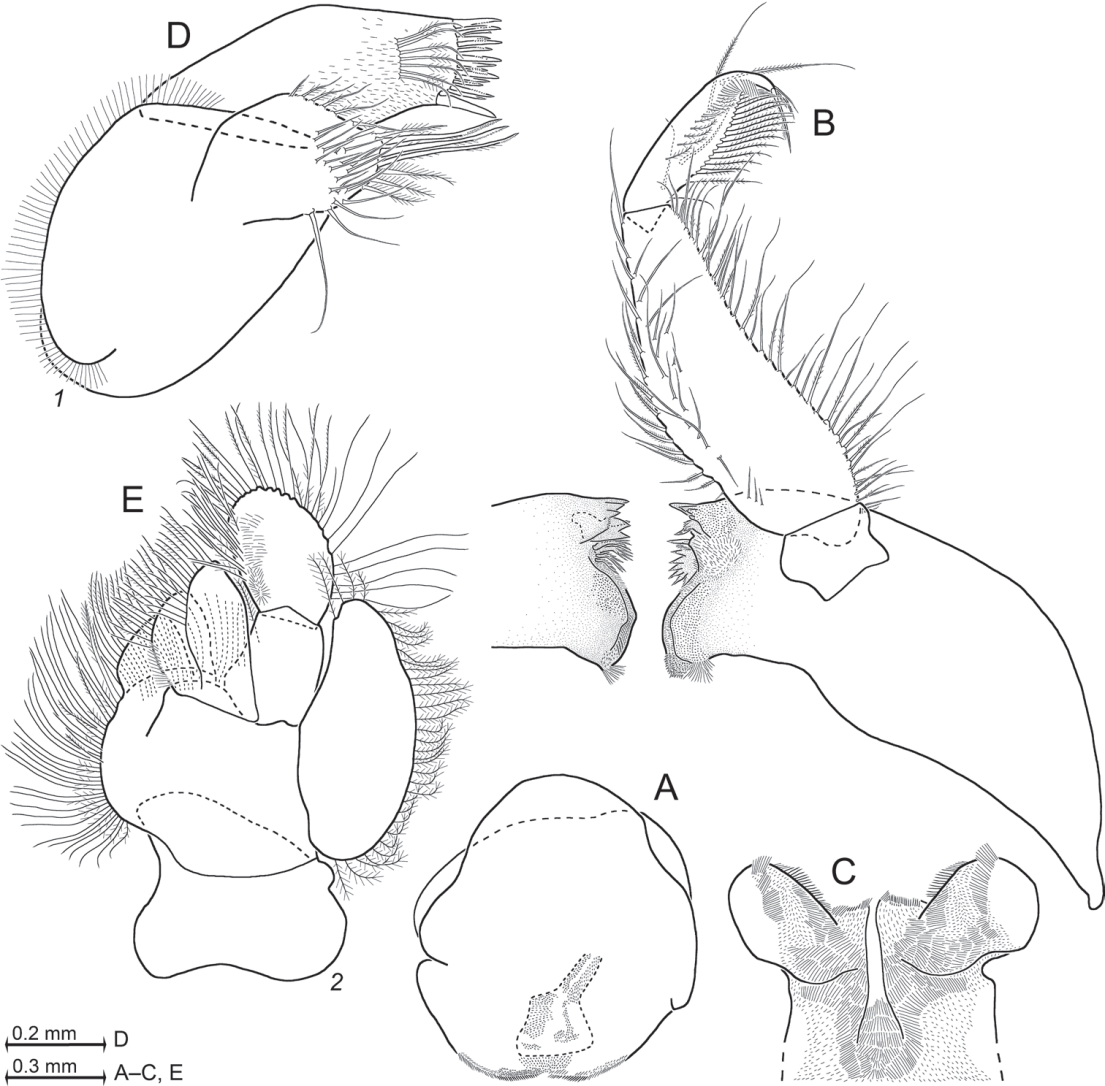
*Mysidetesilligi* from ice cave of Bernard Island, Antarctica. Adult females BL 18.4 mm (**A**, **C**, **E**), 18.1 mm (**B**, **D**) **A** labrum, ventral aspect **B** mandibles with right palp, rostral **C** labium **D** maxillula, caudal **E** maxilla, caudal.

***Mandibles*** (Fig. 11B). Segments 1–3 contribute 9–12%, 56–64% and 26–32%, respectively, to total length of three-segmented palp. Proximal segment of palp without setae. Median segment 2.8–3.5 times as long as maximum width, both margins setose all along. Terminal segment strongly setose along mesial margin; distal 3/4 in addition with series of shorter setae on rostral face. Left mandible essentially normal, right mandible with modified teeth on pars centralis. Pars incisiva of left mandible with 4–5 large teeth plus a few very small teeth, its digitus mobilis strong, with 3–4 teeth and its pars centralis with four separate, spiny teeth. Pars incisiva of right mandible with 4–5 large teeth, digitus mobilis small with one large and 5–6 very small teeth, pars centralis distally with two separate spiny teeth and proximally with 5–7 acute teeth projecting from a common basis. Pars molaris with well-developed grinding surface in both mandibles; part of grinding lamellae with minute teeth.

***Labrum and labium*** (Fig. 11A, C). Caudal face of labrum with field of small, stiff bristles to left and with rugged, spiny area, also with stiff bristles to right. Densely setose field on posterior half of oral (= dorsal) face. Labium normal, comprising two hairy lobes with short, dense set of stiff bristles on distal half of mesial face.

***Maxillula*** (Fig. 11D). Distal segment of maxillula terminally with 11–15 strong spines, most of which are serrated by small denticles in median portions. No such denticles on the largest spines in innermost (mesial) position, weak or no denticles on the spine in outermost (lateral) position. Distal segment subterminally with 7–9 barbed setae, furnished with comparatively long barbs along their median third and minute barbs in comb-like arrangement along distal third; about 8–11 pores beneath basis of outermost seta. Endite of maxillula terminally with three distally-spiny setae, flanked by 2–4 proximally thick barbed setae; mesial and lateral margins of endite with numerous less thick setae; innermost (mesial) seta longest, projecting mesially.

***Maxilla*** (Fig. 11E) normal, densely setose, with various types of setae, but no spines or teeth. Mesial margin of sympod with 1 (2) basally thick seta, barbed in central portions, microserrated by minute stiff bristles along distal third; slightly or not extending beyond dense brush of barbed setae. Terminal segment of endopod plus sympod and all three large endites of sympod, with densely setose distal margins. Basal segment of endopod with three basally thick, barbed setae (below drawing plane in Fig. 11E). Terminal segment 1.4–1.7 times longer than wide. The setae along its lateral margin slender, sparsely barbed near basis, not resembling spines. Leaf-like exopod extends shortly beyond the distal margin of basal segment of endopod. Exopod with 32–37 barbed setae all along lateral margin, the subapical setae on inner margin longest, the neighbouring apical seta second longest, both longer and thicker than the remaining ones (all located on outer margin).

**Figure 12.**
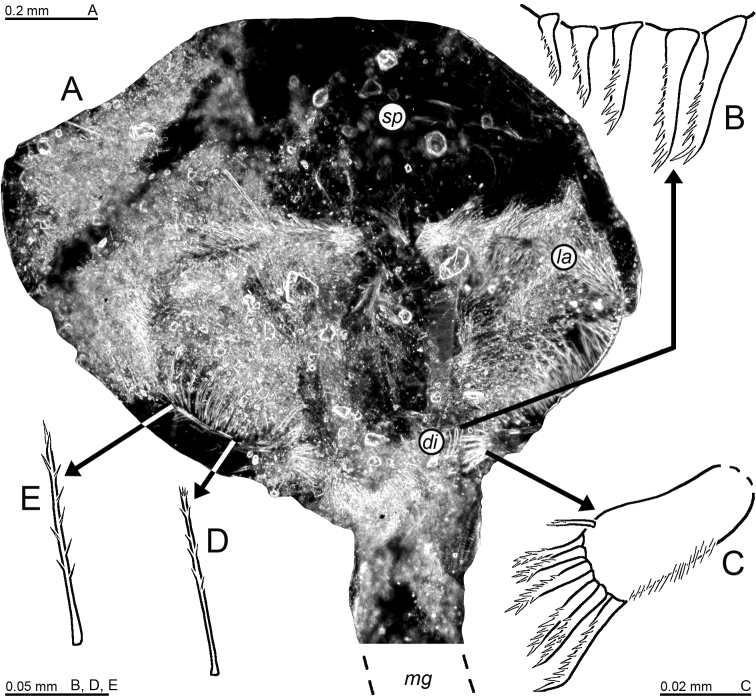
Cardiac portion of foregut in *Mysidetesilligi* from ice cave of Bernard Island, Antarctica. Adult female BL 18.1 mm **A** foregut in dorsal view, food removed from right half, lower-case labels indicate dorsolateral infoldings (*di*), lateralia (*la*), mid-gut (*mg*) and storage space (*sp*) **B** spine group from dorsolateral infoldings **C** spinose lobe of posterior part of lateralia **D, E** spines from median portions of lateralia.

***Foregut*** (Fig. 12). Lateralia mostly covered by smooth acute spines, apically pronged spines (Fig. 12E) and fewer apically forked spines (Fig. 12D), the two latter spine-types with minute spinules along their shaft. Posterior part of lateralia on each side of foregut with lobe bearing dense set of 5–7 unilaterally serrated spines (Fig. 12C). Dorsolateral infoldings on each side with 5–6 spines decreasing in length dorsally-medially, unilaterally serrated in median to subapical portions (Fig. 12B). Foregut not covered by pigment bodies.

***Thoracic sternites*.** Sternite 1 anteriorly produced into an anterior lobe contributing to the caudal closure of the mouth field as usual in Mysidae. Pair of comparatively large barbed setae on intersegmental joint between thoracic sternite 2 and sympod 2. No such setae on sternites 1 and 3–8.

**Figure 13.**
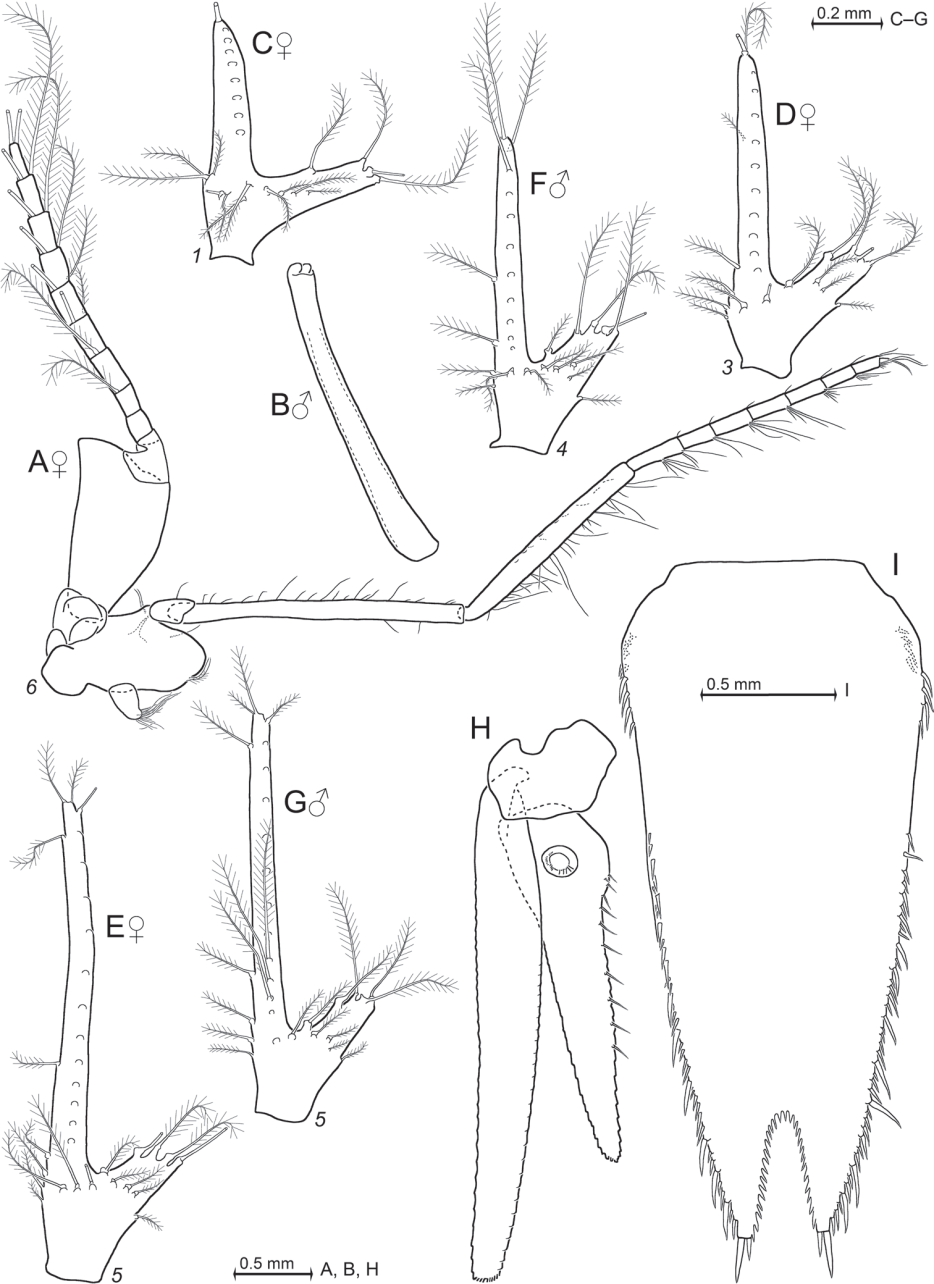
*Mysidetesilligi* from ice cave of Bernard Island, Antarctica. Adult females BL 18.1 mm (**A**, **C–E**, **H**), 18.4 mm (**I**); subadult males 13.7 mm (**B**), 13.1 mm (**F**, **G**). **A** thoracopod 6 including rudimentary oostegite **B** penis of subadult male **C–E** series of female pleopods 1, 3, 5 **F, G** pleopods 4, 5 in subadult male **H** uropods dorsal, setae omitted **I** telson. **C–G**, many setae broken.

***Thoracopods general*** (Figs 10E–K, 13A). Length increasing from exopod 1 to 3, remaining subequal from 3 to 7 and decreasing from 7 to 8. Length of flagella 1.8–2.3 times length of basal plates (Fig. 13A). Exopods with basal plates laterally expanded; length of plates 1.9–2.3 times maximum width. Disto-lateral edge of plates angular, tip rounded to varying degrees. Endopods 5–8 long and slender. Ischium becomes increasingly slender from endopods 1 to 5. Length of ischium increasing from endopods 1 to 5, remaining subequal amongst endopods 5–8. Ischium shorter than merus in endopods 1–4, but longer than merus in endopods 5–8. Dactyli of endopods 1–2 larger than those of endopods 3–4, these latter larger than those of 5–8. Claw 1 strong, weakly bent; claw 2 not developed; claws 3–4 needle-like and shorter than claw 1; claws 5–8 distinctly or indistinctly curved, shorter than claw 3. The first thoracopods with large, leaf-like, smooth epipod.

***Maxillipeds*.** Coxa of maxilliped 1 (thoracic endopod 1) with small endite bearing one barbed seta at its tip. This seta extends across mid-line, thus setae from left and right endite slightly overlapping. Basis with large, prominent endite densely setose on mesial margin. Ischium and merus each with one smaller, but distinct, medially setose endite. Basis of maxilliped 2 (endopod 2) with setose, medially projecting endite. Combined praeischium plus ischium 0.6–0.7 times merus length. Combined carpopropodus plus dactylus measure 1.2–1.3 times merus. Dactylus very large, with dense brush formed by large numbers of normal setae and 14–19 modified setae, the latter apically bent, bearing two symmetrical series of denticles (stiff barbs) on either side in sub-basal to median portions.

***Marsupium*.** Thoracopods 7 and 8 with large oostegites 1, 2, respectively. Each oostegite without setae on upper (dorsal) margin. Ventral margin and part of posterior margin, from sub-basal region up to rounded tip, with dense series of plumose setae, together with bilaterally opposite oostegite forming gate contributing to ventral and caudal closure of marsupium. Basal portions of marsupium inside with comparatively long setae, microserrated on their distal half. Oostegite 1 near basis with about 20 microserrated setae, oostegite 2 with about 8–10. No setae on outer face of marsupium. Thoracopod 6 with rudimentary oostegite (Fig. 13A) represented by small, rounded, smooth lobe bearing 10–13 smooth setae (n = 2) on terminal margin. This rudiment not contributing to wall of brood chamber.

***Pleon*** (Fig. 13C–E). Pleonites 1–5 are 0.6, 0.5–0.7, 0.5–0.6, 0.6–0.7 and 0.6–0.7 times the length of pleonite 6, respectively; thus combined pleonites 4, 5 longer than pleonite 6. No pores found on tergites. Length and slenderness of exopodal portion increasing from first to fifth pleopods. By contrast, thickness of exopodal portion and length of endopodal portion decreasing in this direction (Fig. 13C–E). Scutellum paracaudale subtriangular, terminally well rounded.

***Tail fan*** (Fig. 13H, I). Exopod of uropods 1.3–1.5 times length of endopod and 1.4–2.0 times telson, endopod 1.0–1.3 times telson. Exopod extends 0.2–0.4 times its length beyond endopod and 0.3–0.8 times beyond telson, endopod 0.1–0.3 times its length beyond telson (partly due to telson inserting more rostrally). Exopod of uropods with slightly sigmoid, almost straight lateral margin and clearly convex mesial margin. Endopod with proximal four spines discontinuously increasing in length distally; distally followed by 6–9 longer and more slender spines, subequal amongst each other. Endopod basally with large statocyst containing one egg-shaped, irregularly-discoid statolith with partly moruloid surface, diameter 208–213 µm, height 90–98 µm (n = 6 statoliths from four specimens). Statoliths discoidal, composed of the mineral fluorite. Statolith formula (3–4) + (1–2) + (4–7) + (6–9) + (4–9) = 19–25. Telson (Fig. 13I) 1.2–1.4 times length of ultimate pleonite. Its lateral margins slightly sigmoid, almost straight.

##### Notes on non-adult males.

Immature males are recognised by knob-like appendix masculina with setae bases present, but not yet bearing setae (Fig. 25A). Subadult males by appendix up to half the length of terminal segment of antennular trunk, in part with short setae (Fig. 25B). Penes (Fig. 13B) slender, large, already reaching to thoracic sternites 4–5 in immature males (body size 12–13 mm, n = 2), to sternite 4 up to the maxillula in subadults (11–15 mm, n = 8). No spermatozoa seen inside penes. Pleopods of subadult males (Fig. 13F, G) with same structure and almost same size as in adult females (Fig. 13C–E). No adult males available.

##### Gut contents.

Five adult females (♀0–) and five subadult males inspected in this respect with 30–70% foregut volume filled with largely masticated organic material (debris) plus varying amounts of mineral particles; additional three females (♀0–) with empty foregut. Abundant detritus and mineral particles are visible in Fig. 12A (content artificially removed from the right half of this foregut).

##### Colour and microdistribution.

Live colour was documented in the laboratory (Fig. 7A, B) and in the field (Fig. 7C). Eyestalks, carapace, posterior half of pleomeres and telson densely covered by red pigment spots. Ovarian tubes and brood pouch content red; cornea orange to brown. The animals appear fully red upon ‘expanded’ chromatophores. Many specimens as in Fig. 7C show red cephalothorax and tail fan, but transparent pleomeres 1–5, suggesting a differential ‘expansion’ of chromatophores as also found in many other Mysidae species. The mysids swam several centimetres to several metres away from the substrate, in part within and close to swarms of early stages (Fig. 7C, D) of the nothotheniid fish *Pa.borchgrevinki* (identification R. Causse, MNHN Paris).

##### Distribution and type locality.

First described from samples below ice at the type locality by monotypy, this is Gauss Station, 66°02'S, 89°38'E, coast of East Antarctica. Data of Zimmer (1914) and Lüdecke (2013) combined and refined by present authors: **Gauss Station** is the ‘Winterstation’ of the ‘Deutsche Südpolar-Expedition 1901–1903’ about 85 km north of the continental coast, where the research vessel ‘Gauß’ was locked in ice and drifting with ice for almost one year. Locality with perennial ice cover, except for transient breaks, fissures and holes. The respective sample was taken in 1902 during the austral summer through an artificial hole in the ice, sampling depth from 200–0 m, bottom depth 385 m.

Our findings are the second published with the original name, obtained upon two diving excursions to an ice cave of **Bernard Island**, in 6–10 m depth at 66°39.64'S, 140°01.55'E; this is also at the coast of East Antarctica. It is unclear whether and from where this species previously might have been reported as *M.posthon*. The latter taxon was considered the senior synonym of the present species for almost a century, 1923–2021; the taxon *M.illigi* is now reinstalled.

#### ﻿ Mysidetes hanseni

Taxon classificationAnimaliaMysidaMysidae

Zimmer, 1914

7E52EA6D-C707-5FAF-8A32-E4CFDCD7000D

Figures 14 , 15 , 16 , 17 , 18 , 19 , 20 , 25D , Table 1 , Suppl. material 1

Mysidetes hanseni Zimmer, 1914: 403, 404, Figs 43–46 in Fig.-Tab. XXV (first description); Brandt et al. 1998: Tab. 1 (endemism); Price 2001: 111 (in list, distribution); San Vicente 2011a: 53, Tab. 4, Figs 38N, O (diagnosis, in key); Petryashov 2014: map 11 (biogeography); Mees and Meland 2021: AphiaID = 226498 (accepted).

##### Type series.

Jar (ZMB 18283) labelled “*Mysideteshanseni* Zimmer. Typus. Gauß-Station, leg. D.S.P. Exp.” contains two vials each with one specimen preserved in ethanol. All types sampled (S#9–10) at **Gauss Station**, 66°02'S, 89°38'E, coast of East Antarctica. For prerequisites of lectotype designation, see Discussion.

***Lectotype*** by present designation (Fig. 15). Adult male BL 18.6 mm (ZMB 18283a), vial inside labelled as “D. Südpol.-Exp. 21.12.02 vertikal 200 m. Mysideteshanseni Typ”; S#9.

***Paralectotype*.** Immature male BL 8.7 mm (ZMB 18283b), vial inside labelled “D. Südpol.-Exp. 22.12.02 vert. 250 m. Mysideteshanseni”; S#10.

An additional [transl.] “younger male specimen” reported by Zimmer (1914) is not in the ZMB collection.

##### Non-type material from ice caves.

Total of four samples (S#5–8) taken by P. Chevaldonné and S. Hourdez upon diving in austral summer 2017–2018 in ice caves at the coasts of Curie and Damiers Islands, near Dumont d’Urville Station, Adélie Land, Antarctica:

Six incubating females (♀♀B–) BL 19.3–22.8 mm, 1 ♀0– 19.3 mm, 2 ♂♂A 20.5–22.2 mm, 3 ♂♂S 13.3–17.8 mm, 12 ♂♂I 7.5–12.0 mm (in vials, NHMW 27302, SMF-57649, ZMB 34484, ZMH-K-60868) and 1 ♀B– 23.4 mm (on slides, NHMW 27303), **Curie Islands**, S#5; 5 ♀♀B– 22.0–22.5 mm, 1 ♀S– 20.5 mm, 1 ♂S 18.9 mm (in vials, SMF-57650, ZMH-K-60869) and 1 ♂A 24.7 mm (on slides, NHMW 27304), S#7; 13 ♀♀B– 13.5–24.2 mm, 2 ♀♀0– 16.6–17.8 mm, 5 ♂♂A 17.3–18.1 mm, 2 ♀♀S^+^ 18.8–21.3 mm, 1 ♀S– 19.3 mm, 4 ♂♂S 10.9–13.1 mm, 3 ♂♂I 11.5–12.1 mm, 2 juv. 7.7–7.9 mm (in vials, NHMW 27305, SMF-57651, ZMB 34485, ZMH-K-60870), S#6; 17 ♀♀B– 10.5–22.1 mm, 6 ♀♀0– 17.8–25.7 mm, 3 ♀♀S^+^ 17.2–21.1 mm, 6 ♂♂S 12.3–14.2 mm, 10 ♂♂I 9.2–13.4 mm, 1 ♀I 7.9 mm (in vials, NHMW 27306, SMF-57652, ZMB 34495, ZMH-K-60871), **Damiers Islands**, S#8.

##### Diagnosis.

Diagnosis covers adults of both sexes. Eyes (Figs 15C, 16A, B) well-developed, clearly longer than wide. Cornea roughly calotte-shaped with or without indentation of proximal margin, its length 0.5–0.7 times length of conical eyestalk, diameter 1.0–1.4 times length of terminal segment of antennular trunk. Cornea occupies distal third to half of eye surface. Eyestalk without papilla; length 0.9–1.2 times its maximum width at conjunction with cornea. Rostrum (Figs 15C, 16B, 17D) subtriangular, terminally well-rounded; lateral margins concave (Fig. 17D) to almost straight (Fig. 15C), margins slightly tilted up; 0.3–0.8 times length of terminal segment of antennular trunk (measured along dorsal median line). Antero-lateral edges of carapace well-rounded (Figs 15C, 17D).

***Antennae*** s.l. (Figs 15D, 16C, 17A–C). Appendix masculina (Figs 16C, 17A) strongly setose, measured without setae 0.5–0.8 times as long as terminal segment of antennular trunk, shortly extending beyond anterior margin of this segment. Antennal sympod (Figs 16A, 17C) with one large, acute tooth on disto-lateral edge and, more caudally, an additional shorter tooth. Dorsal face of sympod with lappet-like to triangular lobe, in every case apically rounded. Antennal scale (Fig. 17C) setose all around, apically rounded, two-segmented with apical segment only 2–4% total scale length; scale 4–5 times as long as its maximum width; scale projecting 0.3–0.5 times its length beyond antennular trunk (0.2–0.4 in subadults) and 0.4–0.6 times beyond antennal peduncle.

***Thorax*** (Figs 15, 16C, 17G–K, 19A, B). Right mandible with digitus mobilis and pars centralis modified as in Fig. 11B; remaining mouthparts normal; labrum not produced rostrally; maxilla without spines. Thorax without mid-sternal processes in both sexes. Flagella of thoracic exopods 1 and 8 with eight segments (Fig. 19A), flagella 2–7 with nine segments. Carpopropodites of thoracic endopods 1–8 with 2, 2, 7–8, 7–8, 9–11, 9–10, 9 and 8–9 segments, respectively. Claw of endopod 1 strong, subapically bilaterally serrated; claws 3–8 (Fig. 17H–K) weak, slender, smooth. Female thoracopods 7 and 8 with large oostegites, thoracopod 6 with rudimentary oostegite. Penes (Fig. 19B) tube-like, stiff, slender, smooth all along, without setae. Size variable in adult males: length 1.5–2.5 times length of ischium 8 and 2–3 times merus 8; penes anteriorly extending to thoracic sternites 2–5.

**Figure 14.**
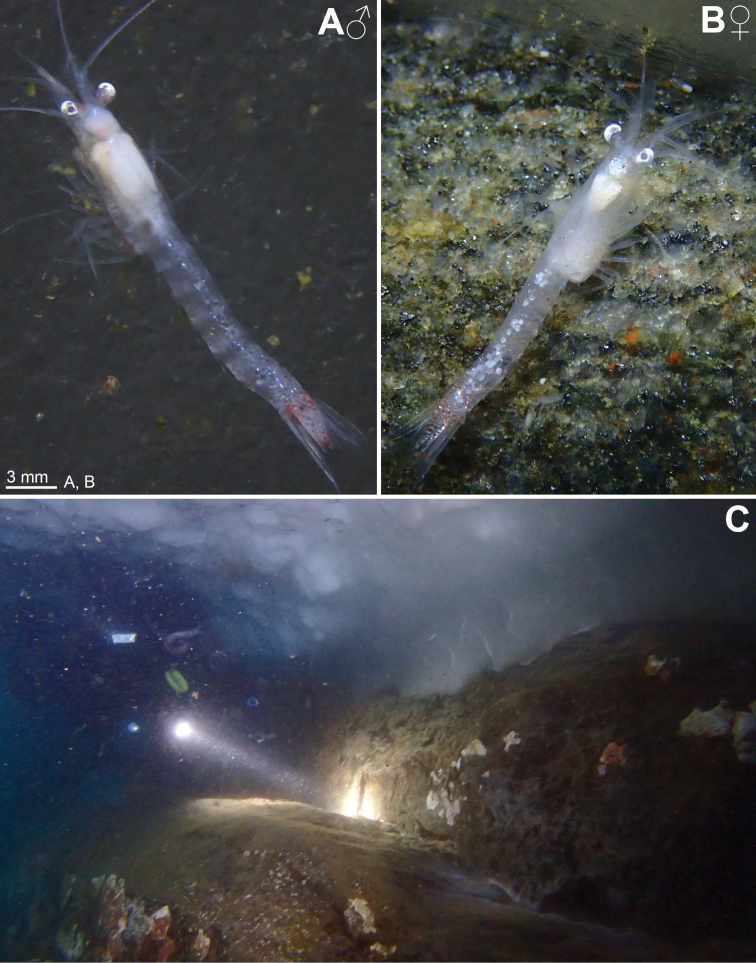
*Mysideteshanseni* in its natural habitat inside ice cave of Damiers Islands, Antarctica **A** adult male, dorsal **B** incubating female, dorsal **C** physical aspect of habitat.

***Pleopods*** (Fig. 19C–E) reduced to undivided, bifid, setose plates with comparatively long endopodal portion (pseudobranchial lobe) in both sexes. All pleopods without spines, no modified setae. Uropods (Figs 16D, 19F) entire, slender, setose all around, no spines; exopod extends by 18–29% its length beyond endopod.

**Figure 15.**
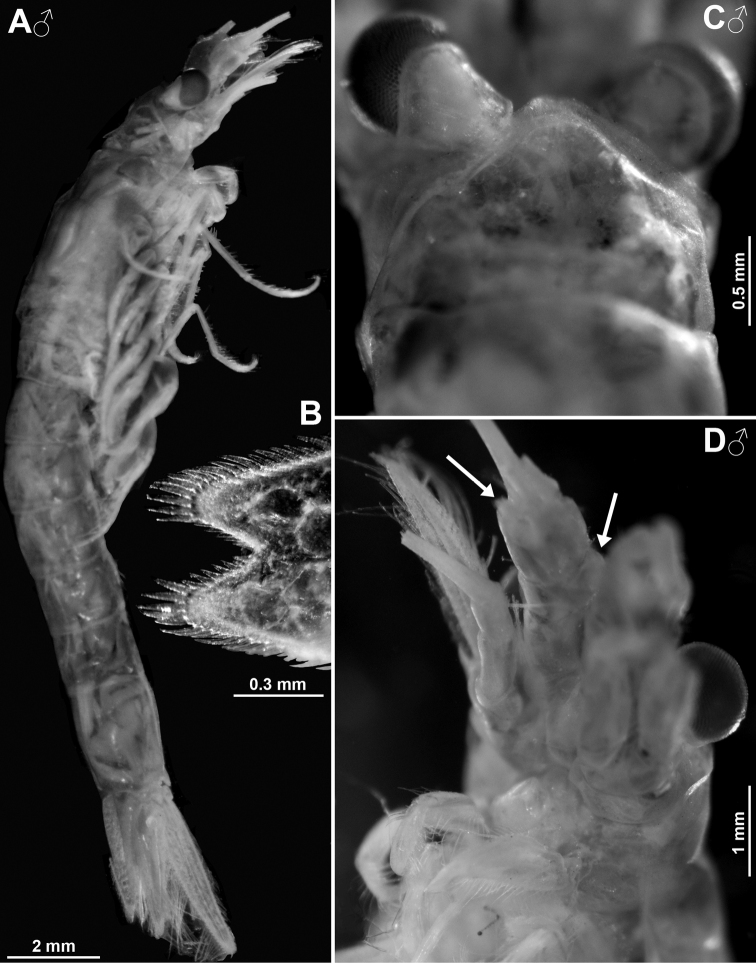
Lectotype of *Mysideteshanseni* Zimmer, 1914, adult male BL 18.6 mm **A** body, lateral **B** terminal fifth of telson **C** anterior body region, dorsal **D** anterior body region, obliquely ventral, left arrow points to remnant of broken appendix masculina, right arrow to mesial swelling of median segment of antennular trunk. **A**, **B**, objects artificially separated from background.

***Telson*** (Figs 15B, 19G) trapezoid, length twice maximum width near basis and 5–6 times width shortly above bifid terminus; 0.7–0.9 times exopod of uropod. Each lateral margin armed almost all along with 45–57 small spines. Sub-basal spine-free portion, if any, up to 1/10 telson length in adults (occasionally longer in non-adults). Spines arranged in consecutive sets on distal half; each set represents series of 2–6 spines increasing in length distally. Triangular apical cleft (Fig. 15B) penetrates 1/10 telson length, margins of cleft lined all along with 14–17 laminae. Telson cleft 1.0–1.8 times as deep as its width at apex. Disto-lateral lobes of telson rounded, each lobe terminally armed with 4–5 strong, subequal spines with 4–5% telson length.

##### Description of types.

The initial objective for inspection of the types was the unclear state of development of male characteristics. Zimmer (1914) indicated the largest specimen examined by him as [transl.] “adult or subadult”. We found a damaged appendix masculina (left arrow in Fig. 15D) with a few setae, apex broken, on the right antennula of the lectotype, suggesting that this appendix was longer *in vivo*, ergo the lectotype considered adult.

Both available type specimens not dissected. Body proportions (Fig. 15A) slender in both specimens as normal in males of *Mysidetes* species. Terminal segment of antennular trunk with 3–4 large plumose setae plus a number of smaller barbed setae on disto-mesial corner; additional large plumose seta inserted subterminally on mesial margin in both specimens. Rostrum of both specimens short, terminally broad, with slightly sigmoid, almost straight lateral margins (Fig. 15C).

***Lectotype*** (Fig. 15). Cornea roughly calotte-shaped, dorsally with proximal indentation, length 0.5–0.6 times eyestalk, diameter equals length of terminal segment of antennular trunk. Eyestalk without papilla. Median segment of antennular trunk with its mesial face inflated (right arrow in Fig. 15D), indicative of male adulthood. Antennal scale as in diagnosis, apical segment 4% total scale length; scale five times as long as its maximum width; scale projecting 0.3–0.4 times its length beyond antennular trunk and 0.6 times beyond antennal peduncle (Fig. 15D). Flagella of thoracic exopods 1–6 as in diagnosis, flagella 7–8 broken. Carpopropodites of thoracic endopods 4–8 with 8, 10, 9, 9, and 9 segments, respectively. Claw of endopods 4–8 weak, slender, weakly bent and smooth. Penes reach to sternite 4. Pleopods as normal in the genus; length increases from pleopod 2 to 5; pleopod 1 slightly longer than pleopod 2. Uropods as in diagnosis; exopod extends by 1/5 its length beyond endopod (Fig. 15A). Telson as in diagnosis, length five times width shortly above bifid terminus. Length 1.2 times sixth pleonite, 0.9 times endopod of uropod and 0.7–0.8 times exopod of uropod. Left (undamaged) lateral margin all along with total of about 54 spines. Most proximal portion of each lateral margin with seven crowded spines; sub-basal portion with six subequal spines positioned with lower density in continuous series; median to distal portions with about 41 spines densely arranged in consecutive sets of 2–6 spines increasing in length distally. Apical cleft penetrates by 9% telson length. Margins of cleft (Fig. 15B) all along with total of 15 laminae increasing in size distally; largest lamina with 2/5 cleft length. Disto-lateral lobes as in diagnosis; terminal spines longer than subterminal spines.

***Paralectotype*.** Median segment of antennular trunk not inflated as normal in immatures. Penes reaching to sternite 6. Telson conforming well to that of lectotype, taking differences due to body size into account: right lateral margin with total of 35 spines, ten of which in approximately linear arrangement along basal and sub-basal portions, remaining spines more densely set along median to apical portions, arranged in groups as in lectotype. Apical cleft 10% telson length; numbers and relative size of laminae as in lectotype.

##### Colour.

Lectotype with well-pigmented dark cornea (Fig. 15) and large dark-brown patches on the body, the latter often observed as artefacts in century-old preserved material. By contrast, the paralectotype is completely bleached, cornea included. This suggests that the two specimens experienced different treatments before being placed in ethanol.

For evaluation of differences between description by Zimmer (1914) and type specimens, see Discussion.

##### Description of ice cave specimens.

**Figure 16.**
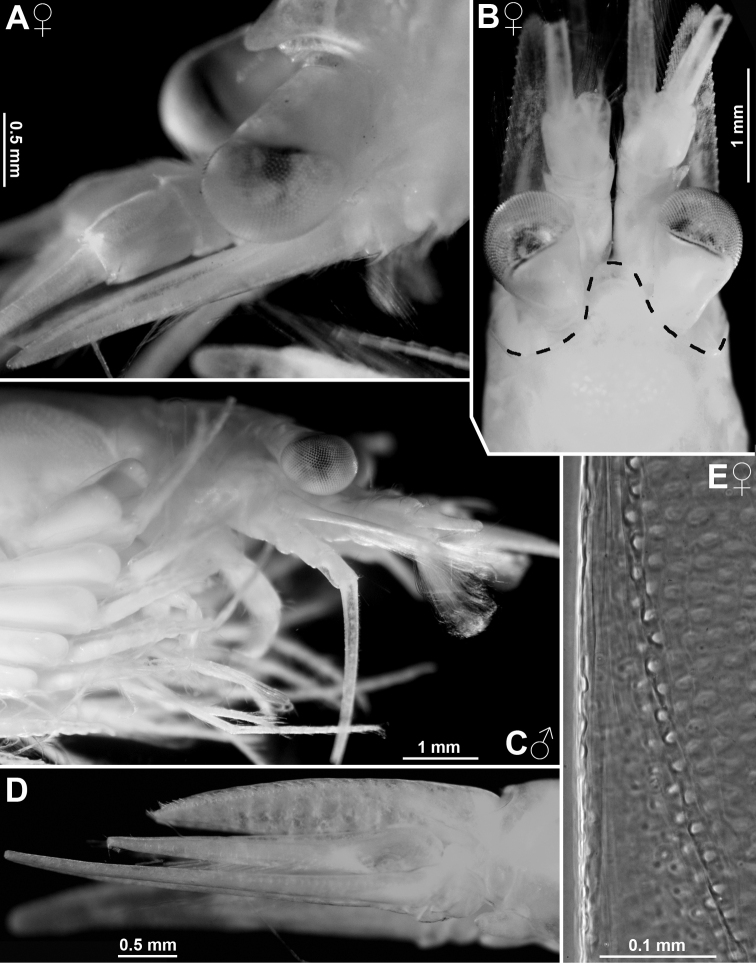
*Mysideteshanseni* from ice cave of Curie Islands, Antarctica. Adult females BL 23.4 mm (**A**, **B**), 21.4 mm (**E**); adult male 24.7 mm (**C**, **D**). **A** head of female, obliquely lateral **B** anterior body region of female, dorsal, dashed line enhances the anterior contour of carapace **C** anterior body region of male, lateral **D** tail fan, lateral **E** cuticle structures on outer surface of the large second oostegite.

Includes re-description of males and first description of females. All features of the above diagnosis. General appearance of females moderately slender (not considering the marsupium), males even more slender. Body length of adult females 10.5–25.7 mm (n = 52), males 17.3–24.7 mm (n = 8). Rostrum comprising 1–3% body length, cephalothorax 32–39%, pleon 47–53%, telson 14–15% and carapace 26–31%. Frons with at least four horizontal bulges (Fig. 16A; potential additional bulges not well verified).

**Figure 17.**
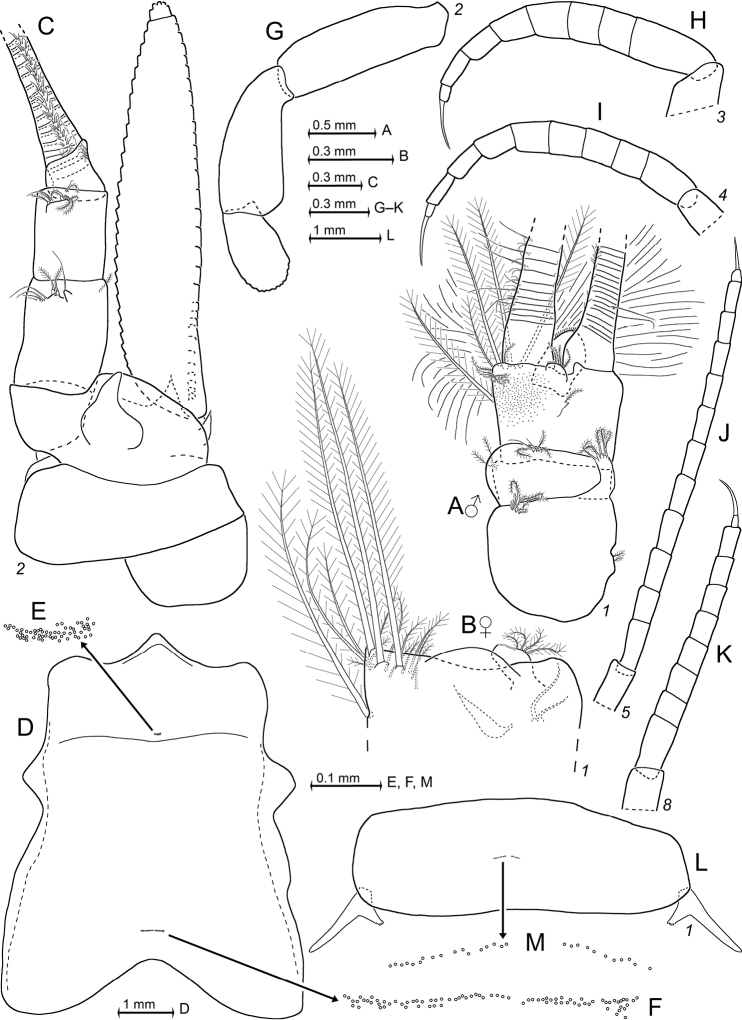
*Mysideteshanseni* from ice cave of Curie Islands, Antarctica. Adult male BL 24.7 mm (**A**, **C–F**, **I**, **J**), adult female 23.4 mm (**B**, **G**, **H**, **K–M**) **A** right male antennula, dorsal **B** distal margin of left female antennular trunk, ventral, flagella omitted **C** antenna with antennal gland, dorsal, setae omitted from antennal scale **D** carapace expanded on slide, details show cervical (**E**) and cardial (**F**) pore groups **G–K** tarsus in series of thoracic endopods 2–5, 8, setae omitted **L** tergite expanded on slide together with pleurites of pleomere 1, setae of pleopods omitted, detail (**M**) shows transverse pore groups. **E**, **F**, **M**, pore diameters not to scale.

***Carapace*** (Fig. 17D) with normal gross structure, without apparent sexual dimorphism. Rostrum covering basal portions of normally orientated eyestalks, reaching at most to middle of artificially straight forward-orientated eyestalks (without cornea). Antero-lateral edges of carapace well rounded, not visibly projecting *in situ*, whereas weakly projecting in artificially expanded carapace. Posterior margin of carapace evenly rounded, mid-caudally well emarginated, leaving ultimate 1–1.5 thoracomeres dorsally exposed. Cervical sulcus strong, cardial sulcus indistinct. Median field of 44–59 crowded pores (Fig. 17E) directly in front of cervical sulcus. Transverse series of 46–81 pores (Fig. 17F) crossing carapace between, if present, cardial sulcus and posterior margin. Except for the here-stated structures, outer surface of carapace smooth in both sexes.

***Eyes*** (Fig. 16A–C). Eyestalks and cornea dorsoventrally (very) weakly compressed (Fig. 16C). In dorsal view, cornea appearing calotte-shaped, in lateral view, oviform with upper margin (= face) slightly flattened.

***Antennulae*** (Fig. 17A, B). Trunk measures 7–9% body length in both sexes, extending 0.4–0.5 times its length beyond eyes, being 1.6–2.1 times longer than maximum width in adult males, 2.2–2.8 in adult females. Measured along dorsal midline, basal segment 42–47% trunk length, median 18–20% and terminal 33–38%. Lateral lobe from basal segment extending beyond median segment. Median segment with its mesial face inflated in adult males only. Terminal segment 0.6–0.9 times as long as wide. Part of terminal segment with cuticle sculptured by minute depressions in males only; due to their small size and density, these depressions drawn as reduced numbers of dots with exaggerated size in Fig. 17A. Details of depressions available in Fig. 16E for oostegite 2. Antennulae of females (Fig. 17B) dorsally with smooth cuticle, not sculptured by minute depressions. Terminal segment of antennular trunk in both sexes with mid-dorsal apophysis bearing four barbed setae on its lateral half and small cilia along its disto-mesial margin; no spiniform anterior projection. Lateral antennular flagellum about as wide as mesial one when measured near basis.

**Antennae** (Fig. 17C). Sympod dorsally with terminally rounded, tongue-like process; caudally with bulbous lobe containing end sac of antennal gland. Three-segmented antennal peduncle in both sexes with basal segment 22–25% peduncle length, second 36–43% and third 32–36%. Third segment 1.1–1.4 times as long as wide. Antennal scale with convex mesial margin; proximal half of lateral margin slightly sigmoid, distal half convex. Small apical segment with five plumose setae.

***Mandibles*.** Segments 1–3 contributing 11–14%, 53–60% and 29–33% length to three-segmented palp. Proximal segment without setae. Median segment 2.7–3.3 times as long as its maximum width, both margins setose all along. Terminal segment strongly setose along mesial margin; distal 2/3 in addition with series of short setae on caudal face near lateral margin. Pars incisiva with 4–5 teeth. Left mandible normal, its digitus mobilis strong, with 3–4 teeth and its pars centralis with 3–4 separate, spiny teeth. Right mandible modified as in *M.illigi* (Fig. 11B), its digitus mobilis small with one large and 3–4 very small teeth, pars centralis distally with one thick spiny tooth and proximally with 3–5 acute teeth projecting from a common basis. Pars molaris with well-developed grinding surface in both mandibles.

Labrum and labium as described above for *M.illigi*.

***Maxillula*.** Distal segment of maxillula terminally with 12–14 strong spines, in part serrated by small denticles in median portions. No such denticles on the largest spine in innermost (mesial) position. Distal segment subterminally with 8–9 barbed setae, of which 7–8 setae densely set in transverse, linear series; 0–2 pore near outermost seta; the remaining 1–2 setae positioned a short distance proximally, out of series. All these setae with barb patterns as in *M.illigi*. Endite of maxillula terminally with three distally spiny setae accompanied by four proximally thick barbed setae; mesial and lateral margins of endite with numerous less thick setae; innermost seta not longest and not projecting mesially as in *M.illigi*.

***Maxilla*** normal, densely setose, with various types of setae, but no spines or teeth. Terminal segment of endopod and sympod including its three large endites, with densely setose distal margins. The leaf-like exopod extends to distal margin of basal segment of endopod or shortly beyond. Exopod with 22–25 barbed setae all along lateral margin, the two apical setae longer and thicker than the remaining ones. Basal segment of endopod with three basally thick, barbed setae. Terminal segment 1.3–1.5 times longer than wide; setae along its lateral margin resembling acute spines, but characterised as modified setae rather than spines based on the densely barbed basal half. Mesial margin of sympod with long seta micro-serrated by minute stiff bristles along its distal third; this seta extending beyond dense brush of plumose setae.

**Figure 18.**
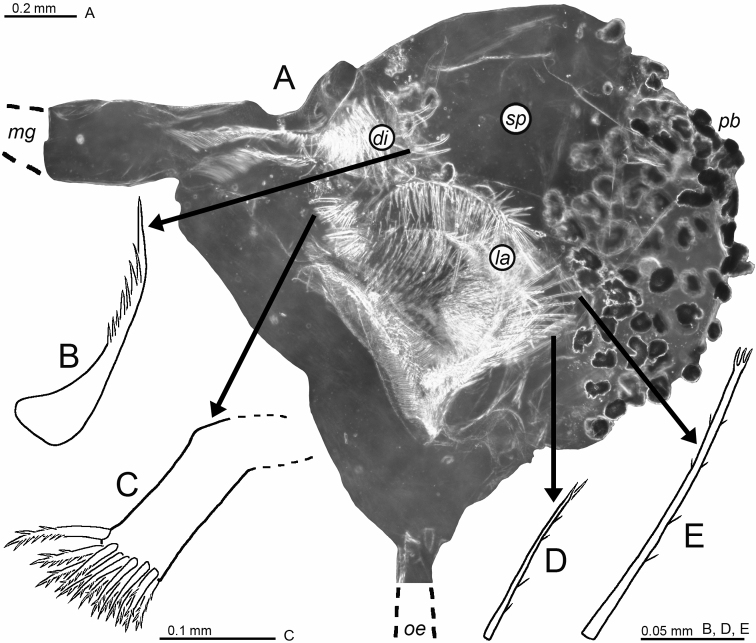
Cardiac portion of foregut in *Mysideteshanseni* from ice cave of Curie Islands, Antarctica; pyloric parts removed. Adult female BL 23.4 mm. **A** foregut in slightly oblique lateral view, lower-case labels indicate dorsolateral infoldings (*di*), lateralia (*la*), mid-gut (*mg*), esophagus (*oe*), pigment bodies (*pb*), and storage space (*sp*) **B** spine from dorsolateral infoldings **C** spinose lobe of posterior part of lateralia **D, E** spines from anterior parts of lateralia.

***Foregut*** (Fig. 18). Spines on most of the lateralia as in *M.illigi*, except for modified spines in Figs 18B and C. Posterior part of lateralia on each side of foregut with lobe bearing dense set of 10–14 bilaterally serrated spines (Fig. 18C). Dorsolateral infoldings on each side with two strong spines, unilaterally serrated in median to subapical portions (Fig. 18B). Dorsal and rostral portions of foregut furnished outside with large numbers of pigment bodies.

Thoracic sternites as described above for *M.illigi*.

**Figure 19.**
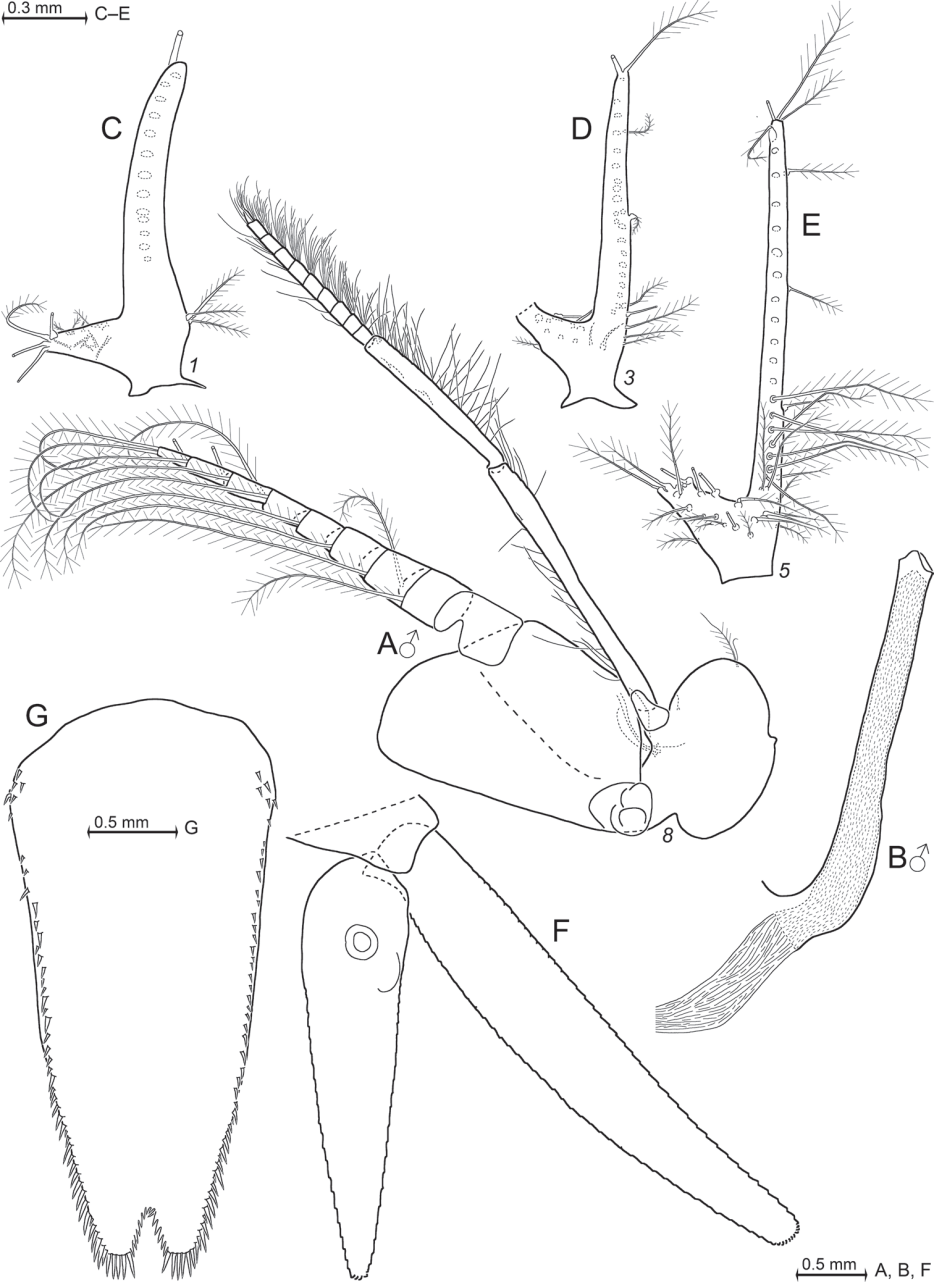
*Mysideteshanseni* from ice cave of Curie Islands, Antarctica. Adult male BL 24.7 mm. **A** thoracopod 8, rostral **B** penis **C–E** series of male pleopods 1, 3, 5, caudal (**C, D**) and rostral (**E**) face, many setae broken **F** uropods, dorsal, setae omitted **G** telson.

***Thoracopods general*** (Figs 17G–K, 19A). Exopods with variable length of flagella and basal plates, no clear size trend along series of exopods 2–7; exopod 1 shorter in both sexes; exopod 8 shorter in females, variable in males. Length of flagella 1.3–2.1 times length of basal plates. Basal plates laterally expanded, length 1.2–2.2 times width. Disto-lateral edge of plates rounded. Endopods becoming longer and more slender from endopod 1 to 5 and decreasing slightly from 5 to 8. Endopods 5–7 long and slender. Ischium becoming increasingly slender from endopods 1 to 5. Length of ischium increasing from endopods 1 to 5 and remaining subequal amongst endopods 5–8. Ischium shorter than merus in endopods 1–4, but longer than merus in endopods 5–8. Dactyli of endopods 1–3 larger than those of endopods 4–8. Claw 1 strong, weakly bent; claw 2 not developed; claws 3–4 slightly bent, equally long; claws 5–8 well or indistinctly curved, shorter than claws 3–4. First thoracopods with large, leaf-like, smooth epipod.

**Maxillipeds**. Combined praeischium plus ischium of maxilliped 2 are 0.8–0.9 times merus length. Dactylus with large numbers of normal setae and 14–17 setae modified as in *M.illigi*. Remaining features as described above for *M.illigi*.

***Marsupium*** (Fig. 16E). Essentially as described above for *M.illigi*, except for setae numbers and cuticle structure. Oostegite 1 near basis with about 30 micro-serrated setae, oostegite 2 with 9–12. Large oostegite 2 with cuticle sculptured by minute depressions over most of its outer surface. These structures resembling scales in episcopic view, but clearly identifiable as depressions in tangential view (both views in Fig. 16E), visible *in situ* already with 15× episcopic inspection. Oostegite 1 with a narrow ribbon of such structures along and close to upper margin, but most of its surface with smooth cuticle, not considering setae. Thoracopod 6 with rudimentary oostegite bearing 10–13 (n = 3) apically microserrated setae.

***Penes*** (Fig. 19B) anteriorly bent at basis. Shaft terminally slightly widened, blunt, ending in 2–3 indistinct lobes. Penes extend anteriorly to thoracic sternites 6–7 in immatures (n = 25), to sternites 3–7 in subadults (n = 14) and to sternites 2–5 (mainly sternite 4) in adults (n = 7).

***Pleon*** (Figs 17L, M, 19C–E). Pleonites 1–5 measure 0.6–0.7, 0.6–0.8, 0.6–0.7, 0.6–0.7 and 0.6–0.7 times the length of pleonite 6, respectively, i.e. combined pleonites 4 and 5 exceed pleonite 6. Tergites 1–7 with transverse linear series of various numbers of pores as in Figs 17L, M. Pleopod structure as described above for *M.illigi*. Pleopods of about same size in both sexes. Length decreasing from pleopod 1 to pleopod 2, remaining subequal amongst 2 and 3 and increasing from 3 to 5. Exopodal portion of pleopod 1 wider than in pleopods 2–5. Its length ranges between that of pleopods 3 and 4. Scutellum paracaudale forming a large acute triangle with slightly concave margins.

***Uropods*** (Figs 16D, 19F). Length of exopod 1.1–1.4 times endopod and 1.1–1.4 times telson, endopod 0.9–1.0 times telson. Exopod extending 0.2–0.3 times its length beyond endopod and 0.2–0.3 times its length beyond telson, endopod 0.1–0.2 times its length beyond telson (partly due to telson inserting more rostrally). Exopod with slightly sigmoid, almost straight lateral margin and clearly convex mesial margin. Endopod basally with large statocyst containing one statolith with diameter of 178–227 µm (n = 8 statoliths from four specimens). Statoliths discoidal, composed of the mineral fluorite. Statolith formula 3 + 1 + (4–7) + (6–7) + (5–9) = 19–23.

***Telson*** (Fig. 19G). Length 1.2–1.3 times length of ultimate pleonite. Basal portion of lateral margins with linear series (rather than aggregated) of 2–5 spines in immatures with 9 mm body length (n = 3) and in three subadults with 11 mm length (n = 3); spine-free sub-basal portion 5–13% of telson length in immatures, 5–17% in subadults and 0–10% in adults (n = 10). Most proximal portion of each lateral margin with 3–7 crowded spines in adults; sub-basal spine-free portion, if any, distally followed by 4–8 subequal spines positioned in a nearly continuous series; median to terminal portions with 31–46 spines densely arranged in consecutive sets of 2–6 spines increasing in length distally.

**Figure 20.**
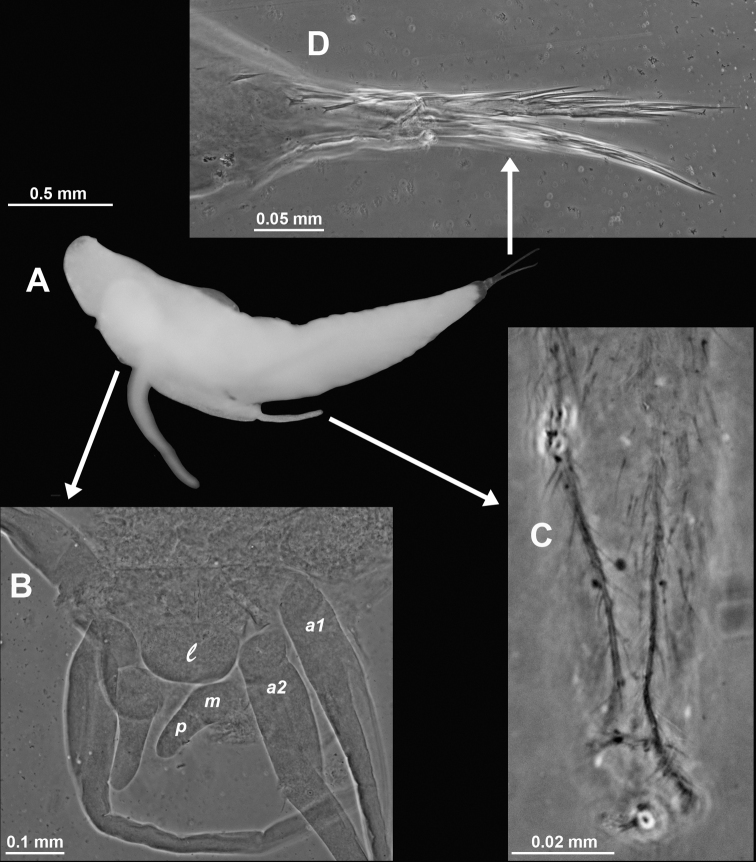
Nauplioid larva of *Mysideteshanseni* from ice cave of Damiers Islands, Antarctica **A** larva at late substage N2, lateral **B** antennae and mouth field, ventral, lower-case labels indicate antennula (*a1*), antenna (*a2*), labrum (*l*) and mandibles (*m*) with palp (*p*) **C** tip of antennula **D** tip of abdomen with caudal furca, lateral. **A**, larva artificially separated from background; **A–D** are from four different specimens.

**Larvae** (Fig. 20). Nauplioids at substages N2 and N3 more slender than in *Heteromysis* S.I. Smith, 1873 species, for example (Wittmann and Abed-Navandi 2021). Twenty-one mounted nauplioid larvae with smooth cuticle, except for antennula, antenna and distal portions of abdomen. Antennae 1 and 2 sparsely covered with minute hairs over distal 2/3 of their length. Density of hairs increases up to tip (Fig. 20C). The old cuticle has started to separate from the tip of the antennula in Fig. 20C, therefore appearing flabby there. Antennula and antenna not yet bifid (Fig. 20B) in all N2-larvae and in most N3-larvae examined. The most striking features of the nauplioids are a pair of long cerci (Fig. 20D), together forming a comparatively large caudal furca armed by numerous spine-like setae. Such spiny setae, together with tiny hairs (as on antennae), are also present on (sub)-apical portions of abdomen. Remaining features in Fig. 20 are typical for the state of development.

##### Distribution and type locality.

Type locality is at the East-Antarctic coast, 66°02'S, 89°38'E (details as given above for *M.illigi*). The types only there were taken in December 1902 with non-closing vertical hauls from 200–0 m (lectotype) and 250–0 m (paralectotype) below ice, bottom depth 385 m (Zimmer 1914). The present records from ice caves in 2–5 m depth at Curie Islands, 66°38.64'S, 140°02.43'E and in 2 m depth at Damiers Islands, 66°39.21'S, 139°57.61'E, are from the second and third localities ever published; see also Discussion.

##### Colour and microdistribution.

Live colour of this species was documented only in the field (Fig. 14A, B). Most specimens showed a whitish tinge of body, eyes, hepatic caeca and brood pouch content. Oil globules (fat bodies) also contributed to the whitish tinge. Globules were found everywhere in the body trunk, with greatest densities above the foregut and in the telson. Comparatively small numbers of red-orange pigment spots were present on eyestalks, carapace, pleomeres, and telson. These were slightly ‘expanded’ mostly on telson, partly also eyestalks. A few specimens (not documented in Fig. 14) showed an overall, weakly red to orange tinge. Corneas appeared white in the field, but were brown in ethanol-fixed materials; therefore, it is not excluded that reflection had contributed to the white tinge in field photos. The mysids were encountered close to and mostly in physical contact with the substrate ice or rock surfaces, with or without epigrowth (Fig. 14A, B).

##### Gut contents.

Upon external inspection of 49 foreguts, all appeared empty in twenty incubating females examined, all in nine spent females available and in eight out of twenty foreguts of immature males. Eight ‘empty’ foreguts dissected and mounted on slides showed that 0–5% volume contained food. Fig. 18 gives an example of a foregut considered ‘empty’ upon external inspection (40´), yet with a few diatoms identified at 200× magnification. The 12 ‘positive’ immature males had 10–40% foregut volume filled. Contents were unidentifiable, masticated organic material (debris), cyanobacteria, diatoms, a few copepod remains and a few mineral particles.

###### Molecular study of ice cave mysids

Figures 21, 22

**Figure 21.**
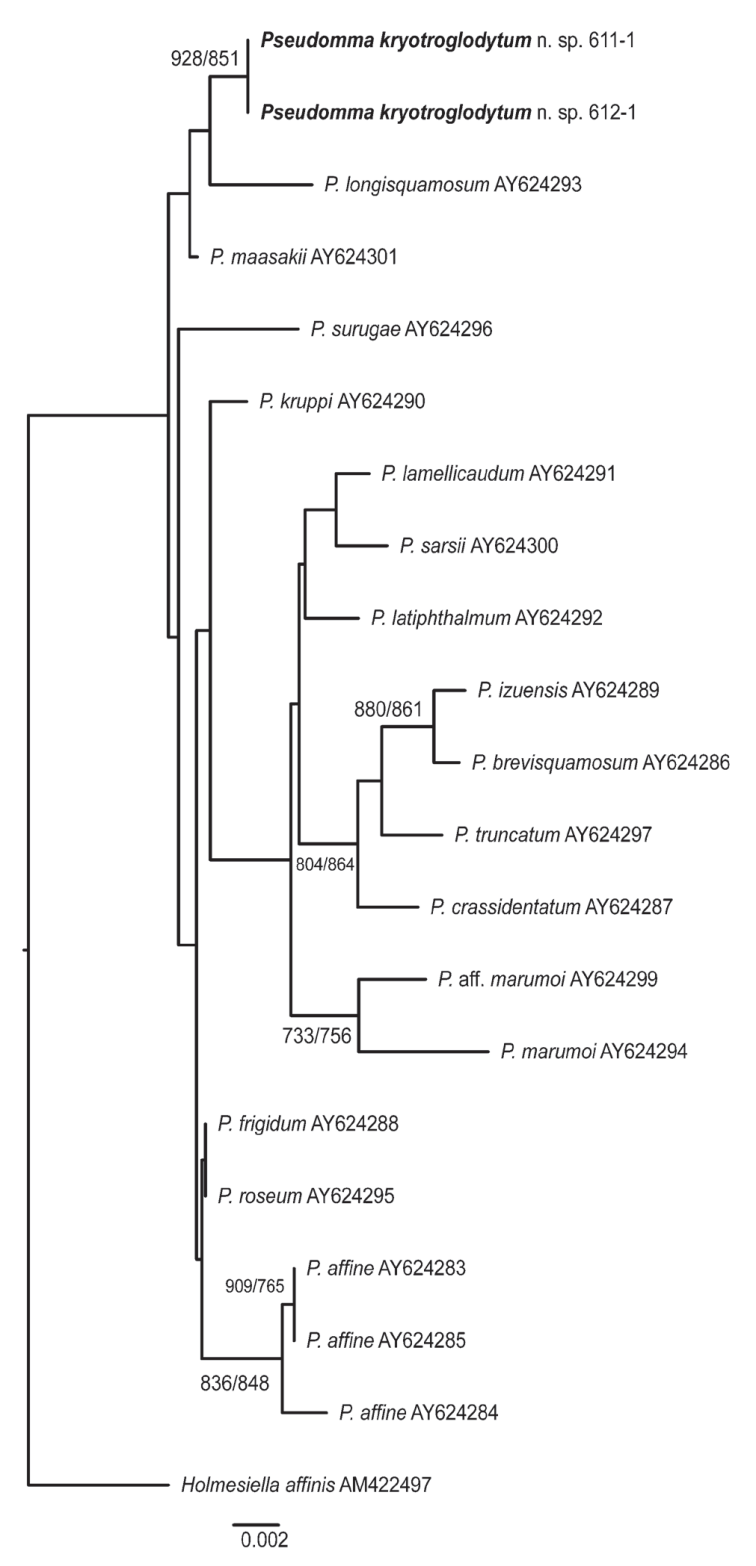
Phylogenetic placement of *Pseudommakryotroglodytum* sp. nov. from Antarctic ice caves at Bernard Island, amongst the *Pseudomma* taxa available in DNA databases (GenBank accession numbers shown), based on 18S rDNA. The root is the Erythropini*Holmesiellaaffinis*. Neighbour-Joining (NJ, shown here) and Maximum Likelihood (ML) reconstruction methods gave a similar topology. Bootstrap (1000 replicates) values, higher than 700, are shown at nodes (NJ/ML) in this order.

**Sequencing of *Pseudommakryotroglodytum* sp. nov.** The 18S DNA sequences obtained from the two here-described specimens of *P.kryotroglodytum* sp. nov. were identical and 805 bp long. COI sequences of unequal quality were obtained from both specimens and were 614 and 658 bp for individuals 611-1 (paratype) and 612-1 (holotype), respectively. Over the alignable part of these sequences, they differed by only one synonymous position. Only 18S sequences could be compared with GenBank sequences of other *Pseudomma* species. We aligned our sequences with 18 available GenBank sequences and obtained NJ and ML phylogenetic trees (Fig. 21) of similar topologies (only NJ shown), rooted with *Holmesiellaaffinis* Ii, 1937 and bootstrapped (1000 replicates for each method). Not even half of the *Pseudomma* species described to date are shown in this tree and only one Southern Ocean species (*P.sarsii*) is available for comparison, but *P.kryotroglodytum* sp. nov. is molecularly different from every other one in the tree. Bootstrap support is poor for most relevant nodes, but two species appear more closely related to *P.kryotroglodytum* sp. nov. in this dataset: *P.longisquamosum* Murano, 1974 and *P.maasakii* Meland & Brattegard, 2007.

**Figure 22.**
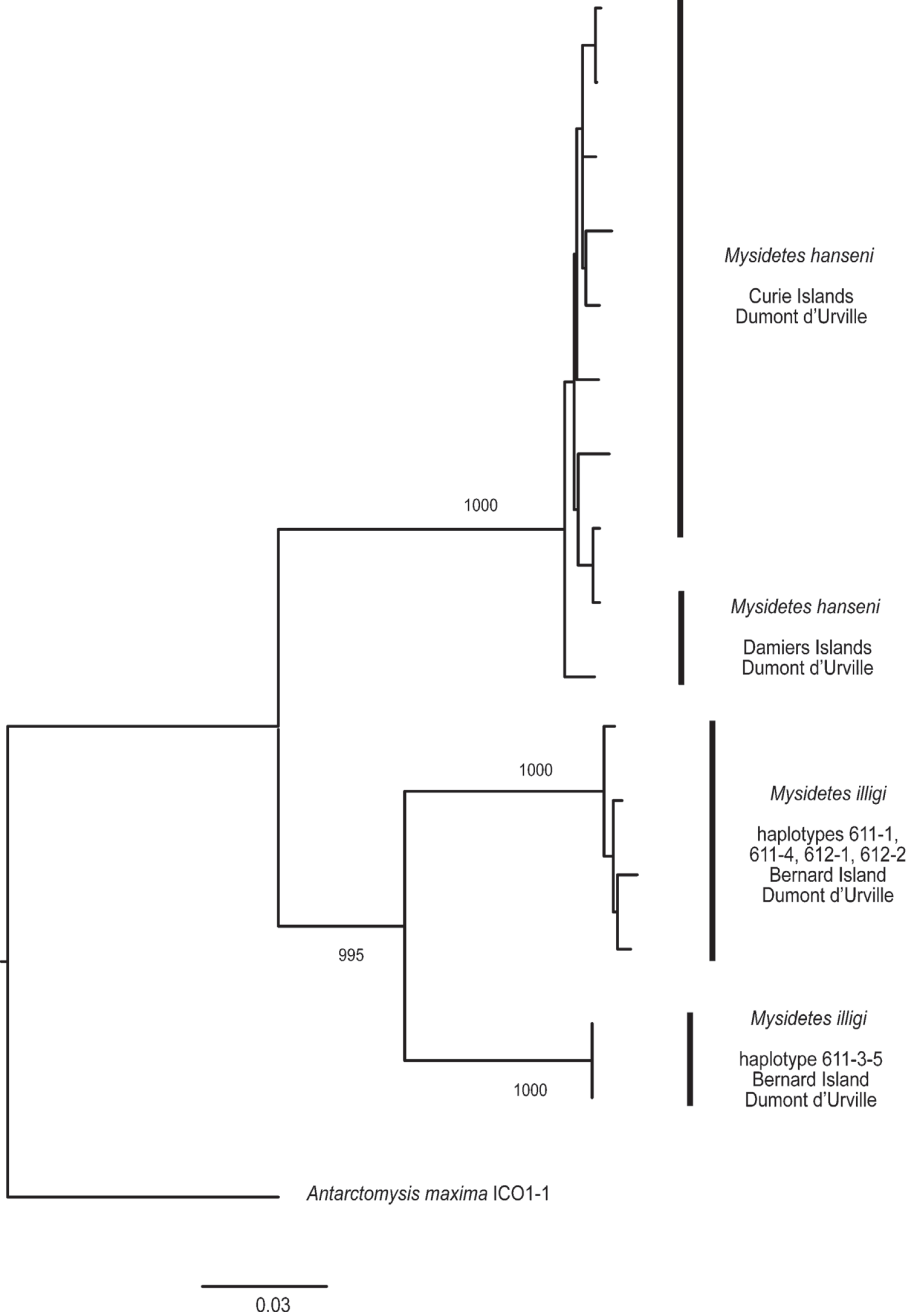
Distance tree (Neighbour-Joining) of mitochondrial COI sequences of *Mysideteshanseni* and *Mysidetesilligi* specimens collected in Antarctic ice caves at Curie and Damiers Islands, Dumont d’Urville Station, rooted with *Antarctomysismaxima* from the same area. Most relevant Bootstrap (1000 replicates) values are shown at nodes.

**Sequencing of *Mysidetesilligi* and *M.hanseni.*** A total of six individuals of *M.illigi* from Bernard Island and 10 *M.hanseni* from Damiers and Curie Islands were sequenced at both the COI and 18S loci. No comparison with GenBank was possible because this is the first time *Mysidetes* sequences are made available. The 18S sequences obtained were 815 and 813 bp long for *M.illigi* and *M.hanseni*, respectively. No differences were observed at this 18S fragment within species, whereas there was a 6 bp difference (but no indel) between the two species. COI sequences of variable quality were obtained (658 to 629 bp), of which 629 bp could be aligned. A simple distance tree (NJ) was produced to visualise the differences and similarities amongst sequences (rooted with *A.maxima*). As evident on Fig. 22, the *M.hanseni* sequences are quite diverse (each specimen displays a distinct haplotype), but the Damiers and Curie specimens are mixed, indicating that no apparent genetic structuring exists at this locus and this geographical scale (3.8 km). In contrast, the Bernard Island *M.illigi* sequences cluster in two divergent groups, which cannot be related either to morphological differences or to collection date and station. The divergence between the two groups is ca. 10% – a high value for intraspecific comparisons, but quite low if they were different species. As noted above, 18S is identical between the two *M.illigi* clusters, as is their translated COI amino acid sequence.

## ﻿Morphology

### First records of structures on eyeplates and antennulae

**Figure 23.**
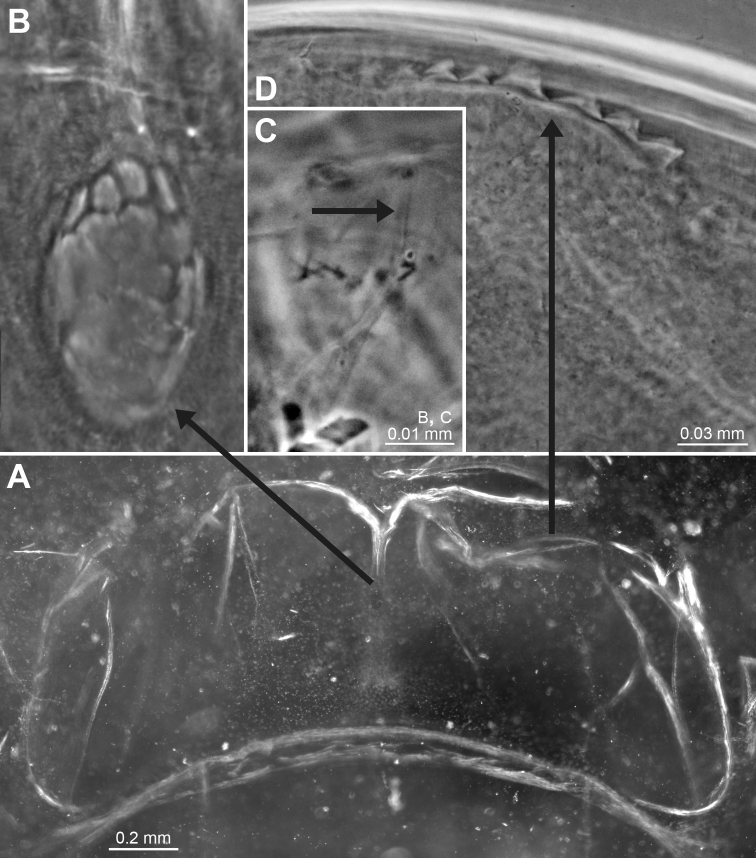
Structure of eyeplates in *Pseudommakryotroglodytum* sp. nov. and *Michthyopsparvus*. Holotype adult female (**D**) and paratype subadult female (**A**, **B**) of *P.kryotroglodytum* and non-type adult female of *M.parvus* (**C**). **A** eyeplate expanded on slide, dorsal, to the right somewhat distorted **B** detail of panel (**A**) showing cyst connected with bottom of median cleft **C** homologous cyst in another species and genus, arrow points to tubular connection with anterior margin of eyeplate **D** series of denticles along sub-lateral section of anterior margin of eyeplate.

**Figure 24.**
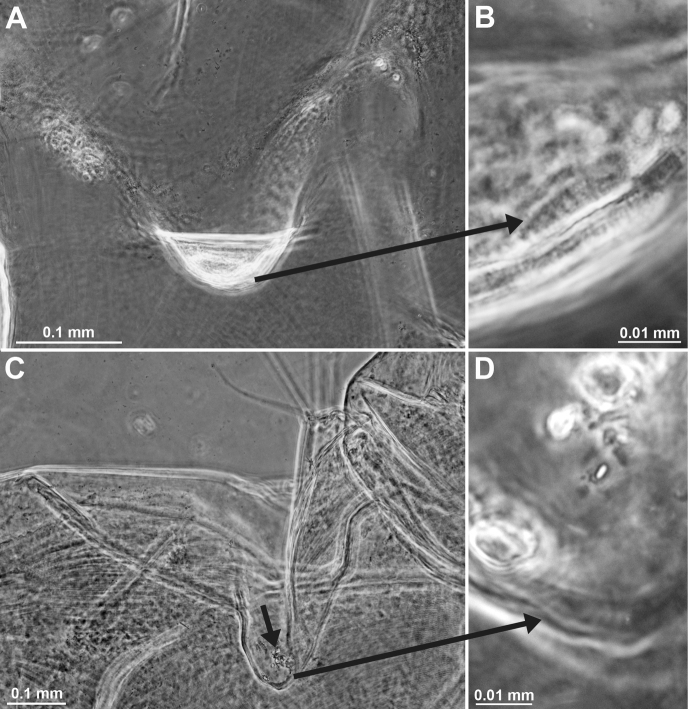
*Pseudommakryotroglodytum* sp. nov., paratype subadult female BL 21.5 mm (**A**, **B**) and *Dactylamblyops* sp., adult female 21.8 mm (**C**, **D**); **A** ventrally orientated depression mid-dorsally on basal segment of right antennula, dorsal aspect **B** detail of panel (**A**), arrow points to striated pad on bottom of depression, dorsal **C** depression as in panel (**A**) for left antennula in another genus, lateral aspect, the short arrow points to mineral particles **D** detail of panel (**C**), the long arrow points to striated pad, lateral. **A**, antennular depression somewhat caudally tilted by the pressure exerted by the cover glass.

**Figure 25.**
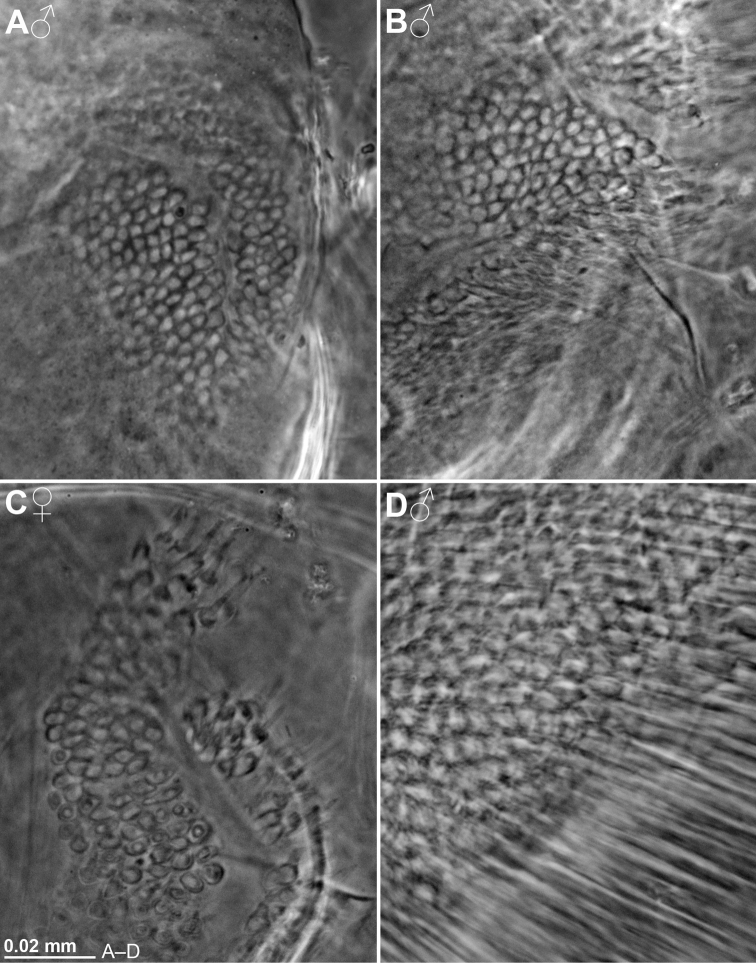
Surface details of appendix masculina and its female homologue in series of increasing body size for *Mysidetesilligi* (**A–C**) and *M.hanseni* (**D**) from Antarctic ice caves. **A** immature male BL 12.1 mm **B** subadult male 14.7 mm **C** adult female 18.4 mm **D** adult male 24.7 mm.

Figures 23–25, Table 1

**Eyeplate cyst.** Eyeplate of *Pseudommakryotroglodytum* sp. nov. contains a single cyst shortly behind the median cleft (Fig. 23A). Cyst egg-shaped, delimited by envelope of cells (Fig. 23B), length about 40 µm, width 25 µm; its rostral projection joins with bottom of eyeplate cleft. Eyeplate cysts (of variable size and shape) found in all seven species of *Pseudomma* inspected (tribus Pseudommini; Table 1).

Species of the tribus Calyptommini are characterised amongst other features by an eyeplate without cleft. Nonetheless, an eyeplate cyst (Fig. 23C) is present in *Michthyopsparvus* (Vanhöffen, 1897), the only species of this tribus examined in this respect (Table 1). Cyst in median position at 30–40 µm distance from anterior margin of eyeplate. Cyst 20 µm long; its converging apical part connected with anterior margin of plate by a narrow tube < 2 µm thick (Fig. 23C).

**Antennular depression.** Basal segment of antennular trunk in *P.kryotroglodytum* sp. nov. shows a mid-dorsal, pit-like, deep, dorsally open, ventrally orientated depression with striated pad on the bottom. Mounted with dorsal face upside, the depression appears pouch-like due to the perspective and partially due to inclination induced by forcing the bent antennula into a plane (Figs 4A, 24A, B). Inspection of unmounted material and of antennulae mounted in lateral position (as in Fig. 24C, D for *Dactylamblyops* sp. A) showed that the depression is actually orientated straight ventrally. Exogenous material present in depressions (Fig. 24B) of left and right antennulae.

Such depressions were found in a total of eleven species of the subfamily Erythropinae (Table 1), none in Heteromysinae (Table 1). Most examined specimens showed exogenous material in the depressions. The short arrow in Fig. 24C points to mineral particles in the left depression of *Dactylamblyops* sp. A.

**Female antennular lobe.** Subadult and adult females of *M.illigi* show a mid-ventral lobe (Figs 8D, 10B) on distal third of terminal segment of antennular trunk, where the appendix masculina is positioned in males. Lobes of adult females with fields of densely-set, only 10–20 µm long, conical setae emerging from a socket collar (Fig. 25C). The homologous setae of subadult males are more numerous and longer (Fig. 25B). Immature and subadult females with low lobe as in the holotype (arrows in Fig. 8D); dissected lobes without (n = 5) or with (n = 3) fields formed by setae bases, no setae shaft visible as also in immature males (Fig. 25A).

No comparable structures were found upon examination of adult females of three other congeneric species (Table 1). Females of these species have more setae (Fig. 17B) on the terminal segment of the antennular trunk compared with *M.illigi* (Fig. 10B), not counting setae of its mid-ventral lobe. No female antennular lobes were found in the remaining species of the subfamily Heteromysinae examined (Table 1). By contrast, female lobes were found in four non-ice cave species of the genus *Dactylamblyops* Holt & Tattersall, 1906 (subfamily Erythropinae) listed in Table 1: two species with comparatively large female lobes with long setae, though lobes and setae shorter and setae less numerous than in males; two other species with even smaller female lobes bearing minute setae. No species without female lobes were found in material of this genus.

## ﻿Breeding

### Breeding in *Mysideteshanseni*

Figures 26–29

**Figure 26.**
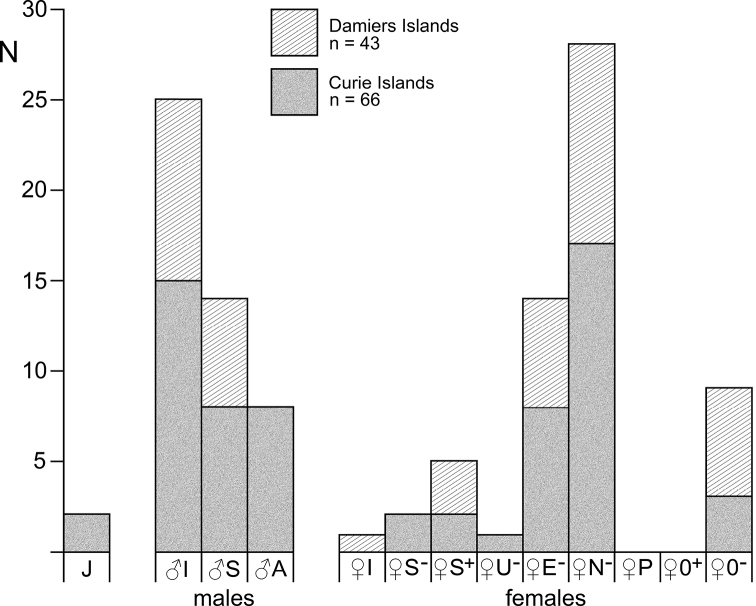
Frequency distribution of free-living stages in samples of *Mysideteshanseni* from ice caves of two Antarctic islands. Numbers of specimens are given for juveniles (J), immature (♂I), subadult (♂S) and adult (♂A) males, for immature (♀I), empty subadult (♀S–) and expectant subadult (♀S^+^) females and for adult females classified as incubating females bearing unfertilised eggs (♀U–), embryos (♀E–), nauplioid larvae (♀N–) and postnauplioid larvae (♀P), finally for non-incubating reproductive females (♀0^+^) and non-reproductive (spent) females (♀0–).

**Figure 27.**
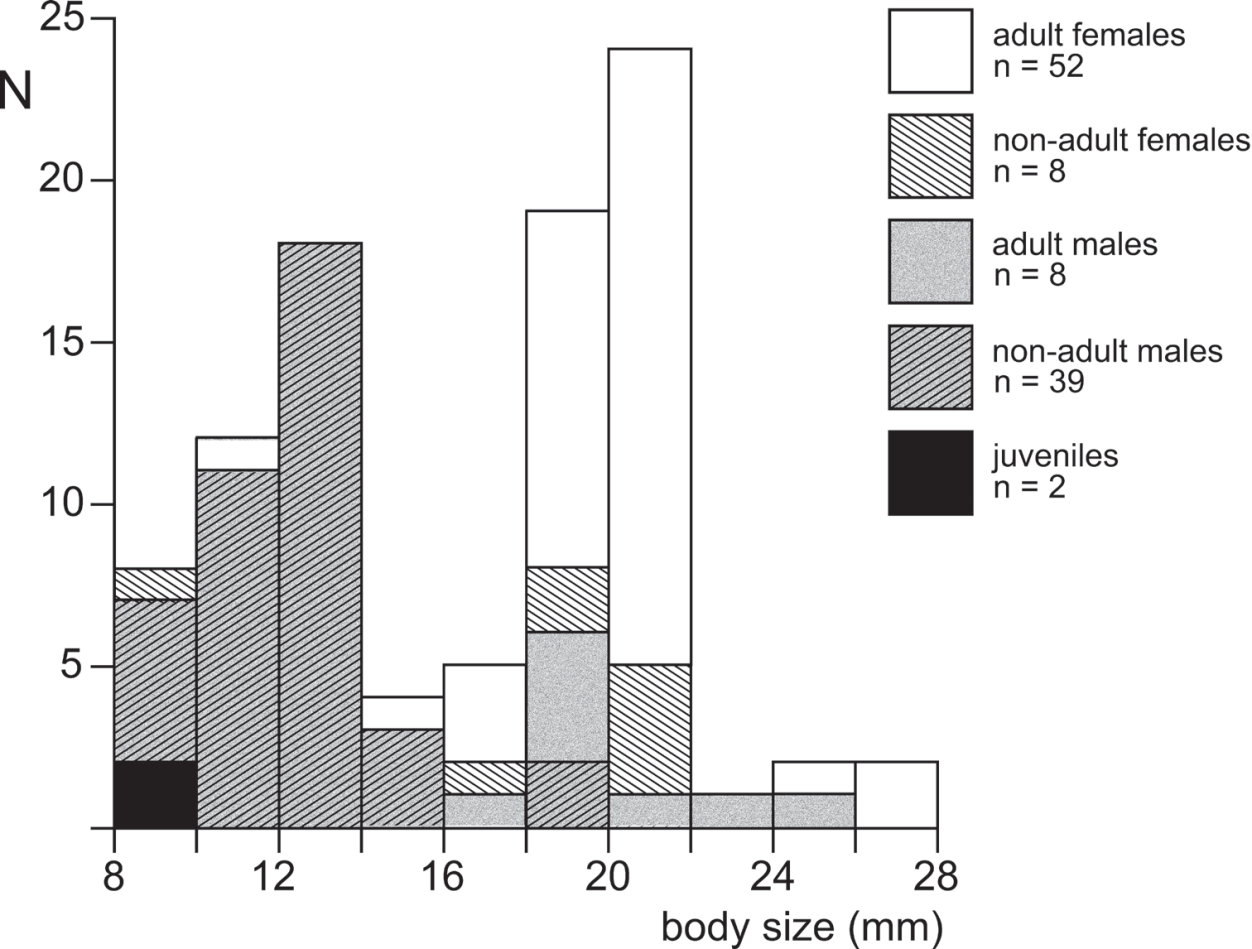
Size-frequency histogram of free-living stages in samples of *Mysideteshanseni* from ice caves of two Antarctic islands. ‘Non-adults’ comprise immature and subadult stages (definition in ‘Methods’).

**Frequency of free-living stages.** Pooled data for the Islands of Curie and Damiers comprises 109 specimens sampled in ice caves, namely 52 adult and seven subadult females, plus eight adult, 14 subadult and 25 immature males, only two juveniles and only one immature female. The frequency of the diverse stages (Fig. 26) does not significantly differ between Islands (*Χ*^2^-test, 4 DF, P = 0.52). The size-frequency distribution in Fig. 27 shows a cluster of small-sized specimens (7.7–14.2 mm, n = 42) comprising mainly immature and subadult males and a cluster of large-sized specimens (16.6–26.1 mm, n = 67) mainly adults of both sexes. Each cluster does not significantly deviate from normal distribution (Anderson-Darling-Test, P = 0.27 and 0.20, respectively). Potential outliers are not supported (Grubbs-Test, P = 0.99 and 0.43). Thus, the overall distribution is bimodal; a potential third mode formed by the four largest specimens in Fig. 27 is not significant. The ovarian tubes were filled with yolk in five out of seven subadult females available, with only empty tubes in all remaining females, regardless of stage. Nine adult females with empty brood pouch, one with unfertilised eggs, 14 with embryos (fertilised eggs) and 28 with nauplioid larvae, none with postnauplioid larvae. Body length of the nine spent (♀0–) females 16.6–26.1 mm, size of breeding females differentiated for marsupial stages below.

**Table 1.** Sensory structures in Mysidae from ice caves and other marine habitats.

Sample nos in Suppl. Table (S#)	Habitat at sampling station	Species	Material examined	Female antennular lobe	Antennul. depression	Eyepl. with corneal sulci	Eyeplate cyst
**Subfam. Erythropinae, tribus Pseudommini** Wittmann, Ariani & Lagardère, 2014							
**1, 2**	**sublittoral ice cave**	*Pseudommakryotroglodytum* Wittmann & Chevaldonné sp.nov..	1 ♀ ad., 1 ♀ subad.	none	present	present	present
**11**	bathybenthic	*Pseudommaaffine* G.O. Sars, 1870	2 ♀♀, 1 ♂	none	present	none	present
**12**	(from deep sea fish stomach)	*Pseudommaaffine* G.O. Sars, 1870	1 ♀, 1 ♂	none	present	none	present
**13**	bathybenthic	*Pseudommaantarcticum* Zimmer, 1914	1 ♀, 1 ♂	none	present	none	present
**14**	bathybenthic	*Pseudommacalmani* O.S. Tattersall, 1955	1 ♀	none	present	none	present
**15**	bathybenthic	*Pseudommalatiphthalmum* Murano, 1974	2 ♀♀	none	present	none	present
**16**	bathybenthic	*Pseudommaroseum* G.O. Sars, 1870	2 ♀♀	none	present	none	present
**17**	bathybenthic	*Pseudommasarsii* Willemoës-Suhm [in G.O. Sars, 1884]	1 ♀	none	present	none	present
**Subfam. Erythropinae, tribus Calyptommini** W.M. Tattersall, 1909							
**18**	(from deep sea fish stomach)	*Michthyopsparvus* (Vanhöffen, 1897)	2 ♀♀	none	none	none	present
**Subfam. Erythropinae, tribus Amblyopsini** Tchindonova, 1981							
**19**	bathybenthic	*Amblyopsabbreviatus* (G.O. Sars, 1869)	1 ♀	–	present	–	–
**Subfam. Erythropinae, tribus Erythropini** Hansen, 1910							
**20**	bathypelagic	*Dactylamblyopshodgsoni* Holt & Tattersall, 1906	1 ♀, 1 ♂	comp. large lobe with normal setae	present	–	–
**21**	bathypelagic	*Dactylamblyopsiii* Nouvel & Lagardère, 1976	1 ♀, 1 ♂	small lobe with minute setae	present	–	–
**22**	mesopelagic	*Dactylamblyopsmurrayi* W.M. Tattersall, 1939	1 ♀, 1 ♂	small lobe with minute setae	not detected	–	–
23	bathybenthic	*Dactylamblyops* sp. A	1 ♂	–	present	–	–
**24**	bathybenthic	*Dactylamblyops* sp. A	1 ♀	comp. large lobe with normal setae	present	–	–
**25**	(from deep sea fish stomach)	*Erythropsmicrops* (G.O. Sars, 1864)	1 ♀, 1 ♂	none	none	–	–
**26**	(from deep sea fish stomach)	*Meterythropspictus* Holt & Tattersall, 1905	1 ♀, 1 ♂	none	none	–	–
**Subfam. Heteromysinae, tribus Mysidetini** Holt & Tattersall, 1906							
**5, 8**	**sublittoral ice caves**	*Mysideteshanseni* Zimmer, 1914	3 ♀♀, 3 ♂♂	none	none	–	–
**4**	**sublittoral ice cave**	*Mysidetesilligi* Zimmer, 1914	5 ♀♀ ad., 4 ♀♀ non-ad., 9 ♂♂ non-ad.	with modified setae	none	–	–
**27**	sublittoral	*Mysideteskerguelensis* (Illig, 1906)	1 ♀	none	none	–	–
**28**	bathybenthic	*Mysidetesposthon* Holt & Tattersall, 1906	1 ♀	none	none	–	–
**29**	sublittoral	*Mysidetesposthon* Holt &Tattersall, 1906	1 ♀	none	none	–	–
**30**	bathybenthic	*Mysifaunerigens* Wittmann, 1996	1 ♀, 1 ♂	none	none	–	–
**Subfam. Heteromysinae, tribus Harmelinellini** Wittmann, Ariani & Lagardère, 2014							
**31**	sublittoral marine cave	*Harmelinellamariannae* Ledoyer, 1989	1 ♂	–	none	–	–
**32**	(aquarium tank)	*Harmelinellamariannae* Ledoyer, 1989	1 ♀, 1 ♂	none	none	–	–
**Subfam. Heteromysinae, tribus Heteromysini** Norman, 1892							
**33**	sublittoral marine cave	*Heteromysisekamako* Wittmann & Chevaldonné, 2017	2 ♀♀, 2 ♂♂	none	none	–	–
**34**	(unknown)	*Heteromysisproxima* W.M. Tattersall, 1922	1 ♀, 1 ♂	none	none	–	–
**35**	sublittoral cryptic habitats	*Heteromysissabelliphila* Wittmann & Wirtz, 2017	1 ♀, 2 ♂♂	none	none	–	–
**36**	sublittoral micro-caves	*Ischiomysispeterwirtzi* Wittmann, 2013	1 ♀, 2 ♂♂	none	none	–	–

**Figure 28.**
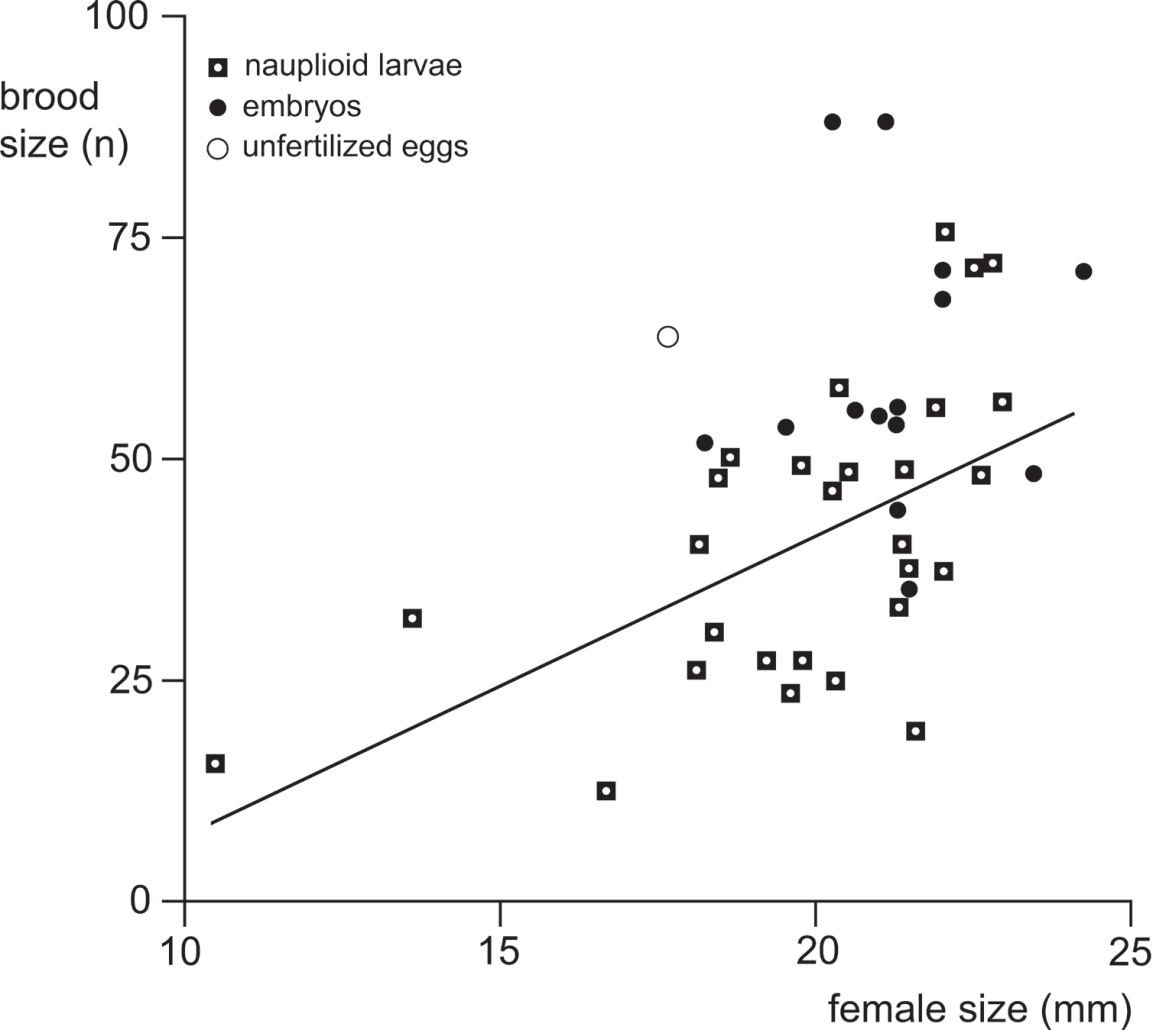
Clutch size in relation to body size of incubating females of *Mysideteshanseni* from Antarctic ice caves. Different symbols are given for unfertilised eggs (n = 1 brood), embryos (fertilised eggs; n = 14), and nauplioid larvae (n = 28). A significant linear regression was obtained and drawn only for nauplioid larvae.

**Clutch size versus parent length.** Fig. 28 shows the variations of clutch size with parent body length. Egged females with 17.7–24.2 mm, clutch size 35–88 eggs. Diameter at embryonic (egg) stage E1 is 0.40–0.66 mm (median of 0.59 mm; total of 792 eggs carried by 13 females); one female 21.3 mm with 44 substage E2-eggs with 0.58–0.67 mm diameter. Female 18.2 mm with 26 nauplioid larvae at substage N1 with 1.40–1.45 mm total length, four females 20.3–22.5 mm with 25–71 nauplioids N2 with 1.62–2.00 mm, twenty females 10.5–22.9 mm with 16–75 nauplioids N3 with 1.80–2.45 mm and three females 20.3–22.8 mm with 48–78 nauplioids N4 with 2.17–2.50 mm.

Contrary to expectations, the females with eggs are significantly larger (t-test, 31 DF, P < 0.05) than the females with nauplioid larvae: body length of E1-females is 21.44 ± 1.57 mm (± SD; n = 13), that of N3-females 19.52 ± 3.01 mm (n = 20). The individual data for clutch sizes of all marsupial stages sampled are given in Fig. 28. Clutch sizes also differ significantly between substages E1 and N3: 60.92 ± 15.47 E1-eggs versus 39.75 ± 14.47 N3-nauplioids (t-test, 31 DF, P < 0.01). To consider the uneven size intervals of parent body length, the clutch sizes are more adequately compared within the interval of parent body length (18.2–22.9 mm) shared by both types of incubating females. Clutch sizes differ significantly also in the reduced dataset: 61.18 ± 16.13 (n = 11) E1-eggs versus 41.59 ± 14.35 (n = 17) N3-nauplioids (t-test, 26 DF, P < 0.01); the respective variances are not significantly different (F-test, 16 v. 10 DF, P = 0.65). Numbers of nauplioids (N) versus parent size (L, in mm) give a significant linear regression: N = 3.358 × L – 25.520 (t-test, 26 DF, P < 0.01; r = 0.57). This is not significant for egg clutches (11 DF, P = 0.34; r = 0.16); therefore, no respective regression line is drawn in Fig. 28.

**Figure 29.**
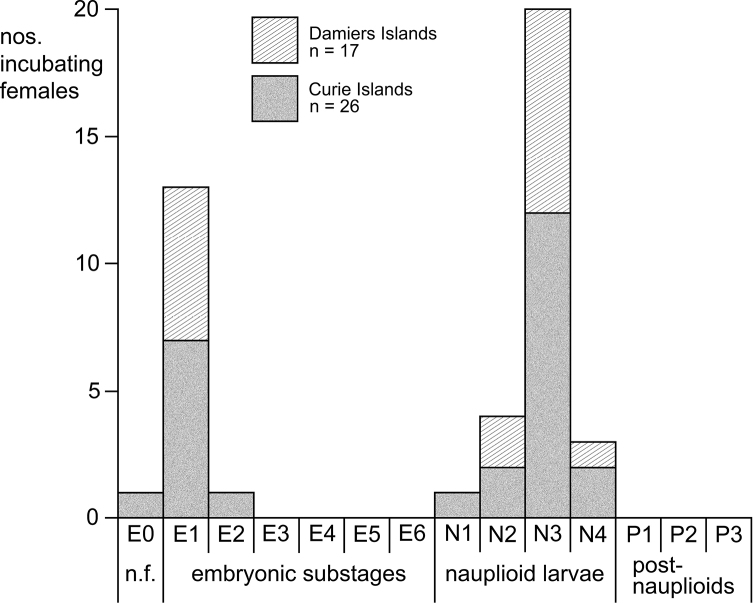
Frequency distribution of incubating females with respect to marsupial stages and respective substages in samples of *Mysideteshanseni* from ice caves of two Antarctic islands. Numbers of specimens are given for unfertilised eggs (E0), embryos (substages E1 to E6) and nauplioid larvae (N1 to N4); only zero counts for postnauplioid larvae (P1 to P3).

**Frequency of marsupial substages.** Fig. 29 shows the frequency distribution of females related to the stages and associated substages in the marsupium. The frequency of the substages does not significantly differ between the two islands inspected (*Χ*^2^-test, 1 DF, P = 0.90). Two cohorts are distinguished, namely early embryonic (egg) substages and moderately advanced nauplioid larvae, the cohorts separated by a wide gap due to the absence of advanced embryonic stages.

**Inhomogeneous clutches.** Not included above are nauplioid larvae that occurred in small numbers in marsupia, together with a main bulk of eggs (embryos) or of younger larvae. Three females with 52, 88 and 68 E1-eggs carried additional 2, 3 and 4 N3-larvae, respectively. Another female with 88 eggs carried two N2-larvae plus two N3-larvae. One N3-larva appeared amongst 13 N2-larvae in the brood pouch of yet another female. All remaining marsupia contained homogeneous broods.

## ﻿Discussion

### Validity of *Pseudommakryotroglodytum* sp. nov.

Only five *Pseudomma* species, so far described in this respect, share smooth lateral margins in combination with a transversely truncate (rather than convex) terminal margin of the telson with the new species:

*P.antarcticum* Zimmer, 1914, is widely distributed in 278–3425 m depth in the Southern Ocean, according to Meland and Brattegard (2007) and also found at 1800–2300 m depths in the Iceland Basin (N-Atlantic). It differs from the new species by shorter apical lobe of the antennal scale, endopod of uropods with a small spine below statocyst and by more (3–4 pairs) spines on terminal margin of the telson.

*P.bispinicaudum* Murano, 1974, from 100 m depth in the East China Sea, differs from the new species by endopod of uropods with a small spine below statocyst and a small tooth on each disto-lateral edge of the telson.

*P.intermedium* Murano, 1974, from 570–660 m depth in waters off Japan (NW-Pacific), differs from the new species by shorter apical lobe of the antennal scale, endopod of uropods with a small spine below statocyst and by more (3–4 pairs) spines on terminal margin of the telson.

*P.maasakii* Meland & Brattegard, 2007, from 1250–2300 m depth in the Iceland Basin (N-Atlantic), prior to first description reported by Murano and Mauchline (1999) as *Pseudomma* sp. from the stomachs of fish in the Rockall Trough (NE-Atlantic). It differs from the new species by shorter apical lobe of the antennal scale, endopod of uropods with a small spine below statocyst and by more strongly converging lateral margins of the telson.

*P.matsuei* Murano, 1966, from ?–1000 m depth in waters off Japan (NW-Pacific), differs from the new species by shorter disto-median fissure of the eyeplate, shorter apical lobe of the antennal scale, more strongly converging lateral margins of the telson and by disto-lateral spines shorter than submedio-apical spines of the telson.

Uropods and telson are unknown in *P.australe* (G.O. Sars, 1884) from 60–120 m depth in the Bass Strait, South Australia. It differs strongly from all remaining so far described species of *Pseudomma* by a very long apical lobe with 4/5 antennal scale length; thus, no detailed discussion needed here.

*P.longisquamosum* Murano, 1974, from 360–460 m depth off Japan (NW-Pacific) is also discussed here due to its genetic affinity (Fig. 21) with *P.kryotroglodytum* sp. nov. It differs by more slender antennal scale (4.5 times as long as wide) with shorter apical lobe only shortly over-reaching the tooth on the lateral margin, by more strongly converging lateral margins of the telson, by presence of spines (11–13 versus none) on lateral margin of the telson and by disto-lateral spines shorter than submedio-apical spines of the telson.

### Detection history of *Mysideteshanseni* and *M.illigi*

Both species were first described by Zimmer (1914) from samples taken through fissures and holes in sea ice in ≤ 250 m depth and ≤ 200 m depth, respectively, at the continental coast of East Antarctica. Many samples were taken by the ‘Erste deutsche Südpolarexpedition 1901–1903’ off the East Antarctic coast, 66°02'S, 89°38'E, where the research vessel ‘Gauß’ was locked in ice for almost one year (Lüdecke 2013). According to Zimmer (1914), the mysid yield was *Hansenomysisantarcticus* Holt & Tattersall, 1906 [ending of taxon name updated], *Pseudommabelgicae* Hansen [in Holt & Tattersall, 1906], *Amblyopstattersalli* Zimmer, 1914, *M.posthon* and *M.hanseni*. From this station, he also described two additional taxa, *M.similis* Zimmer, 1914 and *M.illigi*, both later synonymised by W.M. Tattersall (1923) as *M.posthon*. The synonymy of *M.illigi* is here not accepted as discussed below. A diver from that expedition reported that it was generally dark below ice, with light penetrating through snow at a few spots (Lüdecke 2013). From today’s point of view this habitat shows some similarity with marine ice caves regarding the ceiling, but clearly not regarding the sea floor in 385 m depth nor the water dynamics of the huge water mass compared to ice caves measured in metres to tens of metres.

### Validity of *Mysidetesilligi*

Zimmer (1914) based the description of *M.illigi* as a new taxon essentially on the large, triangular rostrum, the long endopod of uropods and on the spine-free sub-basal portion of the lateral margins of the telson. Without indication of details, Hansen (1921) proposed to check the validity of this taxon, based on more material in the future. W.M. Tattersall (1923) regarded the spine-free portion of the telson as evidence of immaturity. In fact, we found such spine-free portions in immatures and subadults of both *Mysidetes* species from ice caves, though (other than in *M.illigi*) none or very short portions in adults of *M.hanseni*. Furthermore, W.M. Tattersall (1923) reported a large, triangular rostrum and a long endopod of uropod in specimens which he classified as *M.posthon*. Finally, he concluded that *M.illigi* might be a juvenile stage of *M.posthon*. This synonymy was accepted by the scientific community up to the present investigation.

The present data show that adults of *M.illigi* share large spine-free sub-basal portions of the lateral margins of the telson with adults of only *M.antarctica* and *M.kerguelensis*. *Mysidetesantarctica* differs from *M.illigi* by a shorter rostrum and more slender antennal scale, *M.kerguelensis* by a shorter antennal scale and by a proximally unarmed telson. Adult *M.posthon* differ from *M.illigi* by the lateral margins of the telson having spines all along and by more (26–28) spines on the endopods of the uropods. As shown above, females of *M.hanseni*, *M.kerguelensis* and *M.posthon* do not have a ventral lobe on the terminal segment of the antennular trunk (female unknown in *M.antarctica*). This lobe is present in the holotype of *M.illigi*, but was overlooked upon first description by Zimmer (1914). It is here first evidenced and interpreted as a modified appendix masculina, a rare character for females of the family Mysidae, here evidenced also for four otherwise dissimilar species of *Dactylamblyops* (Table 1). Thus, there is no doubt about the validity of this taxon.

### Types of *Mysideteshanseni*

There is no mention of types or any equivalent expression in the original description of this taxon by Zimmer (1914). The present identification of types relies on the inventory of the ZMB. Zimmer indicated 20 mm body length for the largest amongst three specimens sampled by the ‘Deutsche Südpolar-Expedition 1901–1903’. Our measurements gave 18.6 mm for the adult male lectotype (ZMB 18283a) and 8.7 mm for the immature male paralectotype (ZMB 18283b), the third specimen listed by Zimmer (1914) as [transl.] “younger male specimen” is not in the inventory of the ZMB, possibly missing. The text by Zimmer (1914) insinuates that he described the largest specimen. The median segment of the antennular trunk with its mesial face is inflated (as an indication of adulthood) in the lectotype (right arrow in Fig. 15D), not inflated in the paralectotype or in Fig. 43 by Zimmer. The basal portion of each lateral margin of the telson had seven crowded spines (arranged as in Fig. 19G) in the lectotype versus basal spines in linear series in the paralectotype and in Fig. 45 by Zimmer. Nonetheless, the total numbers of spines is about the same in the lectotype and in Fig. 45. The rostrum of both type specimens is terminally broad, with slightly sigmoid, almost straight lateral margins (Fig. 15C); rostrum terminally less broad, both margins strongly concave as given in Fig. 43 and expressly stated in the text (p. 404) by Zimmer. The rostrum of the above-reported ice cave specimens varies from short, broad with almost straight lateral margins (as in Fig. 15C) to weakly produced with biconcave margins (Figs 16B, 17D).

In summary, Fig. 43 by Zimmer (1914) shows a non-adult male whose rostrum shape is the same as found in some parts of the ice cave specimens, but not in the type series kept by the ZMB. The distal 4/5 of the telson in Fig. 45 by Zimmer (1914) fits well with the lectotype, but not with the minor numbers of spines in the paralectotype; by contrast, the proximal fifth of the figured telson fits only with immatures including the paralectotype. In conclusion, the rostrum and telson in Figs 43, 45 and in part of the text by Zimmer (1914) are not consistent with the two available type specimens. We cannot exclude that the shape of the rostrum in Fig. 43 was depicted from the unavailable third “younger” specimen, listed by Zimmer. This appears unlikely for the telson in Fig. 45, which might represent an artificial combination of immature and mature characteristics.

The ZMB holds the main stock of the ‘Deutsche Südpolar-Expedition 1901–1903’. Based on labels of the two available specimens as “Typus”, we define the largest specimen (Fig. 15) as the lectotype, this therefore being the only name-bearing specimen according to the Code, Art. 74 (ICZN 1999).

### Sensory structures

As many as three previously unknown, probably sensory structures were detected by thorough examination of ice cave mysids. The initial expectance of some specificity for ice cave environments was rejected, based on evidence from the subsequent examination of related species from other environments as shown in the following:

#### Female antennular lobe

As first evidence in the family Mysidae, the terminal segment of the antennular trunk bears a low mid-ventral sensory lobe (Figs 8D, 10B, 25C) in females of *M.illigi*. The position of this organ is the same as for the appendix masculina otherwise present exclusively in males of most subfamilies of Mysidae. The lobe size in adult females of *M.illigi* is within the range found in subadult males. The setae bases in females fit within the series of diameters increasing with increasing body size in males (note the uniform scale of panels A–D in Fig. 25). However, females differ from males by fewer, shorter and conical setae (Fig. 25C). Johansson and Hallberg (1992) attributed a chemosensory function to the sensilla (setae) of the appendix masculina in males of two species of Mysinae. The emergence of setae from socket collars (Fig. 25C) in females of *M.illigi* point, in a preliminary interpretation, rather to mechanosensitivity; the shortness of the setae points to near-field reception (cf. DeForest 2014).

No comparable structures were found in adult females of *M.hanseni*, *M.posthon* and *M.kerguelensis* (Table 1). These species, however, show more plumose setae on the anterior margin of the antennular trunk compared to females of *M.illigi*. DeForest (2014) interpreted certain plumose setae on the crayfish antennula as hydrodynamic receptors. If this also holds true for species of *Mysidetes*, then some mechanosensitive function of plumose setae on the antennula might have shifted to the modified setae of the female antennular lobe in *M.illigi*.

Amongst the four species of *Dactylamblyops* examined (Table 1), females of two species showed comparatively large lobes with normal setae that were somewhat shorter than those of males; the remaining two species with small lobes bearing minute setae. Future research could help to establish the incidence of female homologues of the male lobe and their relationship to other sensilla of the antennula in species of Mysidae.

#### Antennular depression

As described above and listed in Table 1 for seven species of *Pseudomma* (tribus Pseudommini), three species of *Dactylamblyops* and one *Amblyops* G.O. Sars, 1872 (Erythropini), the basal segment of the antennular trunk bears a mid-dorsal, ventrally orientated depression with striated pad at the bottom (Figs 4A, 24). The depressions contained mostly exogenous material (Fig. 24C). If these animals were decapods, one would identify such depressions as statocysts. For mysids, however, a static function appears unlikely due to the simultaneous presence of a large statocyst in the tail fan. No such depressions were detected in the ten species of Heteromysinae examined (Table 1). Potential evidence in this respect in additional taxa of the family Mysidae requires further examination.

The central position of the depression dorsally on the basal segment of the antennula in 11 out of 15 Erythropinae species studied points to analogy rather than homology with the Tattersall organ. The latter organ is located dorsally on the antennula in more proximal position close to eye rudiments in *Hansenomysis* Stebbing, 1893 and *Bacescomysis* Murano & Krygier, 1985 (Petalophthalmidae). Additionally, the remote taxonomic status of the here-studied Mysidae versus Petalophthalmidae makes a potential homology unlikely. Siewing (1956) interpreted the Tattersall organ in *Hansenomysis* as a rudimentary statocyst, but no statoliths could be identified. O.S. Tattersall (1961) assumed a chemosensory function, based on the presence of small, rounded, stainable areas. Casanova & De Jong (2005) described this organ in *Bacescomysis* as a pit-like depression with circular aperture overlapped by a flap at the bottom and interpreted it with some reservation as a potential chemosensory organ.

#### Eyeplate cyst

The finding of eyeplate cysts in all examined species with eyeplates (Table 1), namely seven species of *Pseudomma* (tribus Pseudommini) and *Michthyopsparvus* (Calyptommini), points to an important role in species without visual elements. A cavity with an envelope of cells (rather than cuticle) and with a connection to the exterior is characteristic of the Organ of Bellonci which is found on eyestalks, typically at some distance from the cornea, in many species of Peracarida and other crustacean superorders (Hallberg and Chaigneau 2004). The eyeplate cysts differ from this organ by, amongst other features, a long distance from the surface bridged over by a fissure or tube. A potential homology requires confirmation by data on fine structure, which were not available with the here-used methods. Hallberg and Chaigneau (2004) generally assume a chemosensory or a photosensitive function of the Organ of Bellonci and do not exclude that the function may vary between the diverse crustacean groups. In the here-discussed mysids, the ‘long’ distance between eyeplate cysts and surface points to a chemosensory rather than photosensitive function. We do not exclude that the cysts together with fissures or tubes could form an integral chemosensory organ. In any case, additional data are needed to support such hypotheses.

### Biogeography and the ice cave habitat

From the scarce data available, all three mysid species encountered in ice caves are considered Antarctic polar endemics living beyond 66°S. No large-scale horizontal migration has been documented so far for any mysids. Accordingly, these animals probably have to cope with the long, dark, polar winter, when survival requires adaptation to life in darkness below ice cover. Such adaptations could also help to inhabit ice caves during the summer, as well as to survive under large accumulations of pack ice during break up periods. Marine ice caves are ephemeral structures requiring short-term to medium-term immigration by mysids. This is remotely reminiscent of three species of *Hemimysis* G.O. Sars, 1869, that show circadian migration in and out of marine caves in the Mediterranean to feed (Wittmann 1978a; Ledoyer 1989; Benzid et al. 2006; Rastorgueff et al. 2011).

*Pseudommakryotroglodytum* sp. nov. is so far known only from an ice cave in shallow (10 m) marine waters at Bernard Island, East Antarctica, 66°39.64'S, 140°01.55'E. This peculiarity makes it, to our knowledge, the shallowest *Pseudomma* ever found. Both females sampled showed moderately filled foreguts, possibly indicating that they found food, such as the debris on rock and ice surfaces (Fig. 2) within the ice cave. This would be a major difference from the *Hemimysis* example above because this troglophilic habit would allow this species to simultaneously benefit from a shelter from predators and a feeding ground. Alternatively, it is possible that, during the hours of decreased light, the mysids leave the ice caves to feed primarily outside, much like their Mediterranean counterparts. Wherever they feed, another habitat must exist for *P.kryotroglodytum* sp. nov., from where it can regularly recolonise shallow-water dark habitats such as ice caves.

*Mysidetesilligi* was previously recorded only from the type locality, namely the Gauss Station about 85 km north of the continental coast of East Antarctica, 66°02'S, 89°38'E, where it was sampled through holes in ice with a vertical non-closing haul 200–0 m, bottom depth 385 m. The present record from an ice cave in 6–10 m depth at Bernard Island shifts the eastern distributional limit to Adélie Land, East Antarctica, 66°39.64'S, 140°01.55'E. In this cave, the mysids showed a benthopelagic habit at several centimetres to several metres distance from the substrate, in part staying in swarms of young fish (Fig. 7C, D). The red body colour is also found in other *Mysidetes* species examined in this respect (e.g. *M.posthon* figured in the header of O.S. Tattersall 1965). The food quality in the foregut of the here-studied specimens and the debris visible in Fig. 2 makes it likely that the mysids find some food in ice caves. These data suggest that *M.illigi* could be classified as troglophilic as well. Nevertheless, a deeper habitat likewise must exist, as suggested by the type locality. Therefore, shallow-water dark habitats, such as ice caves, may seasonally attract immigrants from deep-water populations. It is possible that in January 2016, Bernard Island ice caves attracted *M.illigi* individuals originating from different deep-water populations, helping to explain the highly diverging haplotype groups simultaneously observed (Fig. 22).

*Mysideteshanseni* is often cited in literature, though back-tracing led in each case to the type samples, according to Zimmer (1914) taken through holes and fissures in sea ice with non-closing vertical hauls in 200–0 m and 250–0 m depth, respectively, in coastal waters of East Antarctica, 66°02'S, 89°38'E, bottom depth 385 m, 21–22 Dec 1902. The precise sampling depth of the types is unknown. The present records from ice caves in 2–5 m depth at Curie and Damiers Islands shift the eastern distributional limit to Adélie Land, East Antarctica, 66°38.64'S, 140°02.43'E. The ice cave specimens showed an epibenthic habit, mostly in physical contact with the substrate. With reservation due to the potential effect of light reflection, the predominantly whitish tinge of the body, eyes, hepatic caeca and brood pouch content is quite unusual in Mysidae; it may be striking in photic habitats and could thus point to a sciaphilic habit. Most specimens examined in this respect showed empty foreguts even though were not freshly moulted. The comparatively large numbers obtained in ice caves upon a total of four diving excursions to two islands makes a potential erratic occurrence unlikely. Ice caves may represent brooding shelters for this species as discussed below. If so, this species could be classified as life cycle-dependent troglophilic.

### Feeding habits

With the exception of their mandibles, the three mysid species encountered in ice caves share the gross structure of external mouthparts as typical in Mysidae. They also share the masticatory portion of the left mandible as is normal in Mysidae and a strong pars molaris in both mandibles, the latter pointing to the ability to grind hard particles, such as diatoms. With few exceptions, the Mysidae show a uniform construction of the foregut, the main differences being the diverse modifications of spines (Kobusch 1998). Such differences are useful in taxonomy and for estimating feeding habits (Wittmann 2018) as discussed in the following:

*Pseudommakryotroglodytum* sp. nov. is striking due to its very large mandibular palp (Fig. 4E). The palp bears a dense brush of setae, but no spines or teeth. Beyond a sensory function, it may be useful for sweeping great numbers of particles to the mouth area. Maxilliped 2 is also very large (Fig. 3D) and its dactylus extremely setose (Fig. 5F); this appendage also lacks spines or teeth and it may function as a brush as well. Thoracic endopods 3–8 are long and slender, ending in weak, small claws. This excludes the ability of this species to prey on medium-sized to large motile animals.

The masticatory portion of the right mandible is modified as typical of the genus *Pseudomma* by the spine row of the pars centralis present as a number of medium-sized, smooth, acute teeth and a few small ones, rather than ‘serrated’ spines. Such teeth appear capable of pricking and fixing particulate matter. Most spines of the foregut appear weak, but not so a block of numerous blunt teeth arising from a common basis (Fig. 6E). The block remotely resembles molar teeth of mammals; in analogy, coordinated action of left and right blocks could be capable of masticating resistant particles. Overall the equipment of this species points to detritivory and potentially also to herbivory, capable of collecting and breaking small, hard food particles. Moreover, the unusually large storage volume (Fig. 6A versus Figs 12A, 18A) of the foregut points to collection of food with low nutritional quality. The two examined foreguts contained masticated, unidentifiable organic materials and mineral particles, suggesting a prevalence of particles brushed from sediment and rock or ice surfaces.

The two *Mysidetes* species from ice caves share long, slender thoracic endopods 3–8 with short, weak claws; endopod 2 without a claw, endopod 1 with a normal-sized claw. The external mouthparts are normal, well setose, almost identical in both species. No spines on the mandibular palp, maxilla or maxillipeds. Predatory feeding on medium-sized to large motile animals is also excluded in these species. Both species also share modifications of the masticatory part of the right mandible, namely the pars centralis distally bearing one (*M.hanseni*) or two (*M.illigi*) thick spiny teeth and proximally with species-specific numbers of acute teeth projecting from a common basis. This differs between species of *Mysidetes* as shown by Băcescu (1967) who explicitly described and figured equal left and right mandibles (as in most Mysidae) for *M.peruana* Băcescu, 1967, from the Peru Trench at 520 m depth. The respective modified teeth of the right mandible in both ice cave *Mysidetes* might have a function similar to that of *P.kryotroglodytum* sp. nov.

The storage volume of the foregut is about the same in *M.illigi* (Fig. 12A) and *M.hanseni* (Fig. 18A). The structure of modified spines is also quite similar. As a slight difference, *M.hanseni* shows two strong spines (Fig. 18B) on dorsolateral infoldings versus two strong spines accompanied by smaller spines (Fig. 12B), dorsally-medially decreasing in size in *M.illigi*. The latter species shows fewer, but larger serrated spines on the spiny lobe (Fig. 12C) at the posterior part of the lateralia, suggesting a slightly superior ability to masticate hard particles. Gut contents analysed in *M.illigi* were largely masticated organic material (debris) plus small amounts of mineral particles; the same materials plus cyanobacteria, diatoms and copepod remains were found in *M.hanseni*. Both species appear to be detritivorous to micro-herbivorous by brushing food particles from available surfaces. The fraction of foregut volume filled was 30–70% in five subadult males of *M.illigi*, although more was expected, based on the abundance of detritus shown in Fig. 7C for the ice cave at Bernard Island in January 2016. Levels of only 0–40% in twenty immature males of *M.hanseni* suggest a scarce food supply for this species upon inspection of ice caves at Curie and Damiers Islands in January 2018. Nonetheless, the here-studied *M.hanseni* showed an extraordinary large content of fat bodies (Fig. 14B) compared with the two other species (Figs 2A, 7A, B) encountered in ice caves and also with average mysids elsewhere. This fat is clearly accumulated outside caves.

In summary, all three mysid species in ice caves are essentially detritivorous. Pyrzanowski et al. (2019) concluded from the study of an opportunistic fish species that detritivory could represent a feeding strategy for survival in harsh environments. Consuming large quantities of low-quality food could help to survive periods of scarcity of alternative food. Nonetheless, we did not observe large food quantities in the foreguts of the three mysid species, although we do not exclude that this might represent a transient situation. The unusually large storage volume of the foregut of *P.kryotroglodytum* sp. nov. may help balance the strongly fluctuating food availability.

### Breeding

#### Inhomogeneous broods

In *M.hanseni*, four out of 14 marsupia with E1-embryos contained additional 1–4 nauplioid larvae; another marsupium with N2-larvae had one N3-larva. The inhomogeneity of E1-broods is interpreted as the result from the adoption of larvae lost (liberated) by other mothers. Adoption was so far shown in field populations of 19 mysid species and confirmed in the laboratory for 16 species (Wittmann 1978b; Mauchline & Webster in Mauchline 1980; Sato and Murano 1994; Wortham-Neal and Price 2002; Johnston and Ritz 2005). Mysids cannot fix their young tightly to any degree due to the water space required to supply oxygen to the brood. This bears the risk of losing young upon violent movements of the parent, for example, as response to water turbulence or to approaching predators. In most species, less than 1% of incubating females in the field carry adopted young that are older than the main brood; the maximum value was 10% observed by Sato and Murano (1994) in *Nipponomysismisakiensis* (Ii, 1936) from the Pacific coast of Japan. Johnston and Ritz (2005) compared adoption in three species of Tasmania, whereby the species with the highest degree of adoption lives in a habitat close to the shore where physical conditions may increase the probability of losing young.

#### Shifted breeding

The frequency distributions of free-living (Fig. 26) and of marsupial (Fig. 29) stages of *M.hanseni* in summer samples show practically no differences between Curie and Damiers Islands, localities that are only 3.8 km apart. This makes potential erratic data appear unlikely. The main characteristics are the almost complete absence of juveniles and of immature females, the absence of postnauplioid larvae, the bimodal frequencies of marsupial stages (Fig. 29) and the bimodal size-frequency distribution in free-living stages (Fig. 27). The strong peak of E1-embryos in Fig. 29 clearly indicates that the eggs were deposited shortly before sampling in summer. The peak of N3-nauplioids shows that most sampled larvae were long after hatching from the egg membrane and still before the moult that leads to the postnauplioid stage.

If the N3-broods were deposited during the preceding summer, one would expect a total incubation period of about two years – based on extrapolating from the timespan between egg deposition and N3-stage taking about half the incubation period (Wittmann 1981). This would yield 2–3 times the maximum of nine months (Lasenby and Langford 1972) so far obtained for Arctic populations of Mysidae species. Such a long incubation is hardly compatible with the empty foreguts in all adult females of *M.hanseni* inspected by us.

Extrapolation from the summer samples suggests that the wide gap between the modes for E1-embryos and N3-nauplioids points to syntopic co-existence of early breeding and late breeding females. Contrary to the results on a number of other species from temperate (Mauchline 1973) and subtropical climates (Wittmann 1978a), body lengths and clutch sizes of mothers with younger (E1) brood were greater than those with older (N3) brood (Fig. 28). Potential intermoult shrinkage of the body is excluded as a relevant factor here because of the constant contribution (14–15%) of the comparatively rigid telson to total body length. Different body size and fecundity support arguments for a time shift between these breeding types. During such a shift, subadults disposed towards late breeding could profit by prolonged growth and yolk production until moult to the final stage and subsequent egg deposition in summer. If the smaller clutch sizes of N3- versus E1-broods at comparable body length of mothers (Fig. 28) were primarily due to premature loss of young, one would expect a higher variance in the numbers of older (N3) versus younger offspring (E1). This variance, however, is not significant in our material, suggesting that the different clutch sizes reflect a different body-size-specific fecundity of two breeding types.

Breeding shifts were already reported by Ward (1984, 1985) for sub-Antarctic populations of *A.maxim*a and *A.ohlinii* Hansen, 1908: the former species shows maximum numbers of incubating females and of juveniles in April at South Georgia, but in December at the South Orkney Islands. The congener *A.ohlinii* shows shorter time shifts for populations only a few km away in coastal waters of South Georgia: in Cumberland East Bay, maximum numbers of incubating females are found in December, whereas in Moraine Fjord, a tributary of this same Bay, in January. No other breeding schedules are known in such detail in populations of sub-Antarctic and Antarctic species.

#### Life cycle

A biennial life cycle with co-existence of two cohorts at any particular time was reported by Richoux et al. (2004) in a population of the boreal to arctic *Mysismixta* Liljeborg, 1853, from about 240 m depth in a fjord-like bay of Newfoundland. This Arctic locality shares with Antarctic ice caves that the seawater temperature is below 0 °C year round. Our ice cave data for *M.hanseni* from mid-southern summer share an absence of juveniles, a presence of adults of both sexes and a bimodal size-frequency distribution of the free-living stages (Fig. 27; potential third mode not significant as shown above) with data from late northern summer in Newfoundland (sample from 29 Sept 1999 in Richoux et al. 2004). Our findings of (almost) empty foreguts and of empty ovarian tubes in all examined incubating and spent females of *M.hanseni* fit with the conclusions of Richoux et al. (2004) that *My.mixta* females are semelparous and die after releasing the young. The above-documented high content of fat bodies could explain the supposed ability of *M.hanseni* to survive several months without feeding.

Size-frequency distributions are available for the congener *M.posthon* from hyperbenthic samples at diverse stations off the Antarctic Peninsula (Siegel and Mühlenhardt-Siegel 1988; San Vicente et al. 2006). For winter data, Siegel and Mühlenhardt-Siegel (1988) obtained three size-groups by modal analysis according to Macdonald and Pitcher (1979) and interpreted these groups as annual age classes of a 3+ years life cycle. Shortly after the publication by Siegel and Mühlenhardt-Siegel (1988), Parsons and Savard (1989) criticised the method of Macdonald and Pitcher (1979) as depending on starting parameters. Summer data, obtained by San Vicente et al. (2006), show unimodal size-frequency distributions for juveniles and for immatures, whereby adults are rare in these samples.

#### Synopsis of breeding schedules.

In summary, a biennial life cycle and shifted breeding are main strategies affecting the frequency of stages of *M.hanseni* in our summer samples from Antarctic ice caves. A biennial life cycle alone cannot sufficiently explain the bimodal frequency of marsupial stages. A biennial life cycle superimposed by shifted breeding fits with most of our data. The almost complete absence of juveniles and immature females (Fig. 26) in our summer samples does not contradict the proposed scheme as discussed below.

Richoux et al. (2004) estimated an incubation period of five months for the above-discussed Arctic population of *My.mixta*. Wittmann (1984) used a combination of the allometric equation with a variant of the Arrhenius equation to describe variations of the incubation period with egg size and temperature in 23 species (38 populations) of Mysida and Lophogastrida from the Tropics to the Arctic. The resulting equation yields five months (with a 99% confidence interval of 2–11 months) for the estimate for species incubating eggs with a median diameter 0.59 mm at –1 °C (as in *M.hanseni*). Based on the relative durations of marsupial stages in Mediterranean mysids (Wittmann 1981), it is roughly interpolated that about half the incubation period passes between egg deposition and N3-substage.

The timing of marsupial stages suggests that early-breeding females of *M.hanseni* deposit eggs under less favourable trophic conditions in about November, the late-breeding females during the summer bloom in January–February. The smaller body length and lower fecundity in N3- versus E1-mothers could be explained in analogy to findings of Beeton and Gannon (1991) that the freshwater species *My.diluviana* Audzijonyte & Väinölä, 2005 (as *My.relicta*) bears smaller broods at smaller parental body size in an ultra-oligotrophic lake compared to a eutrophic lake. In an evolutionary and ecological interpretation, the price that early-breeding *M.hanseni* pay for an earlier release of young into a presumably less dense population is a lower fecundity.

In an evolutionary context, it is plausible that a biannual life cycle, in combination with shifted breeding, optimises the partition of seasonal food resources between the diverse sex and age stages with different energy demands for individual growth and yolk accumulation in ovarian tubes. Samples from the different seasons inside and outside caves could help to verify the proposed timing of complete breeding cycles and related differences in the state of development, age, body size, fat content and clutch size between cohorts and potential sub-cohorts.

In condensed summary, we found support for a scenario in which the young live outside caves until they are large and fat enough to reproduce and dwell in ice caves as shelter for brooding only once during their remaining lifetime. The evidence for this is the almost complete absence of juveniles and immature females in our ice cave samples versus a high incidence of brooding females with empty foreguts and empty ovarian tubes, but with high contents of oil globules, together with their energy-saving habit to stay on the substrate rather than swimming.

## Supplementary Material

XML Treatment for Pseudomma

XML Treatment for Pseudomma kryotroglodytum

XML Treatment for Mysidetes

XML Treatment for Mysidetes illigi

XML Treatment for Mysidetes hanseni

## Supplementary materials

Supplementary material 1 Detailed sampling data Data type sampling stations, details of sampling, species recorded Explanation note Detailed sampling data for total of 36 samples of Mysidae species from Antarctic ice caves and other marine habitats. https://binary.pensoft.net/file/629340This dataset is made available under the Open Database License (http://opendatacommons.org/licenses/odbl/1.0/). The Open Database License (ODbL) is a license agreement intended to allow users to freely share, modify, and use this Dataset while maintaining this same freedom for others, provided that the original source and author(s) are credited.Karl J. Wittmann, Pierre Chevaldonné

## References

[B1] Audzijonytė A Väinölä R (2005) Diversity and distributions of circumpolar fresh- and brackish-water *Mysis* (Crustacea: Mysida): descriptions of *M.relicta* Lovén, 1862, *M.salemaai* sp. nov., *M.segerstralei* sp. nov. and *M.diluviana* sp. nov., based on molecular and morphological characters. Hydrobiologia 544: 89–141. 10.1007/s10750-004-8337-7

[B2] Băcescu M (1967) Further mysids from the Pacific Ocean collected during the Xlth cruise of R/V Anton Bruun, 1965. Revue Roumaine de Biologie – Zoologie 12(3): 147–159.

[B3] Beeton AM Gannon JE (1991) Effect of environment on reproduction and growth of *Mysisrelicta*. American Fisheries Society Symposium 9: 144–148.

[B4] Benzid D De Jong L Lejeusne C Chevaldonné P Moreau X (2006) Serotonin expression in the optic lobes of cavernicolous crustaceans during the light-dark transition phase: Role of the lamina ganglionaris. Journal of Experimental Marine Biology and Ecology 335: 74–81. 10.1016/j.jembe.2006.02.020

[B5] Boas JEV (1883) Studien über die Verwandtschaftsbeziehungen der Malakostraken. Morphologisches Jahrbuch. Wilhelm Engelmann, Leipzig 8: 485–579. [pls XXI–XXIV]

[B6] Boisduval J-A (1875) 1. Sphingides, Sésiides, Castnides. In: Boisduval J-A Guénée A (Eds) Histoire naturelle des insectes. Species général des Lépidoptères Hétérocères.Librairie Encyclopédique de Roret, Paris 1: 1–568.

[B7] Boulenger GA (1902) V. Pisces. In: Report on the collections of natural history made in the Antarctic regions during the voyage of the “Southern Cross”. British Museum (Natural History), London, 174–189. [pls XI–XVIII]

[B8] Bowman TE Orsi JJ (1992) *Deltamysisholmquistae*, a new genus and species of Mysidacea from the Sacramento-San Joaquin estuary of California (Mysidae: Mysinae: Heteromysini). Proceedings of the Biological Society of Washington 105: 733–742.

[B9] Brandt A Mühlenhardt-Siegel U Schmidt A (1999) Density, diversity, and community patterns of selected peracarid taxa (Malacostraca) in the Beagle Channel, South America. In: Schram FR Vaupel Klein JC von (Eds) Crustaceans and the biodiversity crisis. Proceedings of the Fourth International Crustacean Congress, July 20–24, 1998.Koninklijke Brill NV, Leiden, 1: 541–558.

[B10] Brandt A Mühlenhardt-Siegel U Siegel V (1998) An account of the Mysidacea (Crustacea, Malacostraca) of the Southern Ocean. Antarctic Science 10: 3–11. 10.1017/S0954102098000029

[B11] Casanova J-P De Jong L (2005) A new North-East Atlantic species of *Bacescomysis* (Mysidacea, Crustacea) with original moth-like antennules. Journal of Natural History 39: 1839–1849. 10.1080/00222930400023784

[B12] Chevaldonné P Rastorgueff P-A Arslan D Lejeusne C (2015) Molecular and distribution data on the poorly-known, elusive, cave mysid *Harmelinellamariannae* (Crustacea: Mysida). Marine Ecology 36: 305–317. 10.1111/maec.12139

[B13] Chevaldonné P Sket B Marschal C Lejeusne C Calado R (2008) Improvements to the “Sket bottle”: a simple manual device for sampling small crustaceans from marine caves and other cryptic habitats. Journal of Crustacean Biology 28: 185–188. 10.1651/07-2923R.1

[B14] Czerniavsky V (1887) Monographia Mysidarum inprimis Imperii Rossici (marin., lacustr. et fluviatilium). Fasc. 3. Trudy Sankt-Peterburgskago obshchestva estestvoispytatelei 18: i–viii, 1–102. [pls V–XXXII]

[B15] DeForest Jr M (2014) Chapter 3. Sensory Systems of Crustaceans. In: Derby C Thiel M (Eds) Nervous Systems and Control of Behavior. The Natural History of the Crustacea.Oxford University Press, USA 3: 49–84.

[B16] Griffiths HJ (2010) Antarctic marine biodiversity – What do we know about the distribution of life in the Southern Ocean? PLoS ONE 5(8): e11683. 10.1371/journal.pone.0011683 PMC2914006 20689841

[B17] Griffiths HJ Anker P Linse K Maxwell J Post AL Stevens C Tulaczyk S Smith JA (2021) Breaking all the rules: The first recorded hard substrate sessile benthic community far beneath an Antarctic ice shelf. Frontiers in Marine Science 8: e642040. 10.3389/fmars.2021.642040

[B18] Guindon S Dufayard JF Lefort V Anisimova M Hordijk W Gascuel O (2010) New algorithms and methods to estimate Maximum-Likelihood phylogenies: Assessing the performance of PhyML 3.0. Systematic Biology 59: 307–321. 20525638 10.1093/sysbio/syq010

[B19] Hallberg E Chaigneau J (2004) The non-visual sense organs. In: Forest J Vaupel Klein JC von (Eds) , The Crustacea.Revised and updated from the Traité de Zoologie. Brill, Leiden 1 (7), 301–380.

[B20] Hansen HJ (1908) Schizopoda and Cumacea. In: Resultats du voyage du S.Y. Belgica en 1897–1898–1899. Rapports scientifiques. Zoologie. Expédition Antarctique Belge. J.E. Buschmann, Anvers 3–17. [pls I–III]

[B21] Hansen HJ (1910) The Schizopoda of the Siboga Expedition. Siboga Expeditie, Monographie. E.J. Brill, Leyden 37: 1–77. [pls I–XII] 10.5962/bhl.title.10421

[B22] Hansen HJ (1913) Report on the CrustaceaSchizopoda collected by the Swedish Antarctic Expedition 1901–1903, under the charge of Baron Dr. Otto Nordenskjöld. G.E.C. Gad, Copenhagen, 56 pp. [pls I–VI]

[B23] Hansen HJ (1921) On some malacostracous Crustacea (Mysidacea, Euphausiacea, and Stomatopoda) collected by the Swedish Antarctic expeditions. Arkiv för Zoologi 13(20): 1–7.

[B24] Harmelin JG Vacelet J Vasseur P (1985) Les grottes sous-marines obscures: un milieu extrême et un remarquable biotope refuge. Téthys 11: 214–229.

[B25] Haworth AH (1825) XXIX. A new binary arrangement of the Macrurous Crustacea. The Philosophical Magazine and Journal, London 65(323): 183–184. 10.1080/14786442508628417

[B26] Holt EWL Tattersall WM (1905) Schizopodous Crustacea from the north-east Atlantic slope. In: Report on the Sea and Inland Fisheries of Ireland for 1902 and 1903. Scientific Investigations. Fisheries Branch. Department of Agriculture for Ireland. Dublin. Annual Report, 1902–1903, pt. II, app. IV: 99–152. [pls XV–XXV]

[B27] Holt EWL Tattersall WM (1906a) Schizopodous Crustacea from the Northeast Atlantic Slope. Supplement. Report on the sea and inland fisheries of Ireland. Part II. Scientific Investigations. Fisheries Research Board of Ireland, Dublin, Appendix V (1904): 1–49. [pls I–V]

[B28] Holt EWL Tattersall WM (1906b) Preliminary notice of the Schizopoda collected by H.M.S. Discovery in the Antarctic region. Annals and Magazine of Natural History, ser. 7, 17: 1–11. 10.1080/00222930608562484

[B29] ICZN [International Commission on Zoological Nomenclature] (1999) International Code of Zoological Nomenclature. International Trust for Zoological Nomenclature, London, 4th edn, 306 pp. 10.3897/zookeys.931.51583 PMC7205856 32405237

[B30] Ii N (1936) Studies on Japanese Mysidacea I. Descriptions of a new and some already known species belonging to the genera *Neomysis*, *Acanthomysis*, and *Proneomysis*. Japanese Journal of Zoology 6: 577–619.

[B31] Ii N (1937) Studies on Japanese Mysidacea III. Descriptions of four new species belonging to tribes, Leptomysini and Erythropini. Japanese Journal of Zoology 7: 191–209.

[B32] Illig G (1906) 2. Bericht über die neuen Schizopodengattungen und -arten der Deutschen Tiefsee-Expedition 1898–1899. I. Mysideen. Zoologischer Anzeiger 30: 194–211.

[B33] Illig G (1930) Die Schizopoden der Deutschen Tiefsee-Expedition. In: Chun C (Ed.) Wissenschaftliche Ergebnisse der Deutschen Tiefsee-Expedition auf dem Dampfer “Valdivia” 1898–1899.Gustav Fischer Verlag, Jena 22, 397–620.

[B34] Janssen A Chevaldonné P Martinez Arbizu P (2013) Meiobenthic copepod fauna of a marine cave (NW Mediterranean) closely resembles that of deep-sea communities. Marine Ecology Progress Series 479: 99–113. 10.3354/meps10207

[B35] Johansson KUI Hallberg E (1992) Male-specific structures in the olfactory system of mysids (Mysidacea; Crustacea). Cell & Tissue Research 268: 359–368. 10.1007/BF00318804

[B36] Johnston NM Ritz DA (2005) Kin recognition and adoption in mysids (Mysidacea: Crustacea). Journal of the Marine Biological Association of the United Kingdom 85: 1441–1447. 10.1017/S0025315405012622

[B37] Kemal M Kızıldağ S Koçak AÖ (2019) Further notes on the Heterocera (Lepidoptera) of the Philippines, with a description of a new species. Priamus 18(2): 31–128.

[B38] Klepal W Kastner RT (1980) Morphology and differentiation of non-sensory cuticular structures in Mysidacea, Cumacea and Tanaidacea (Crustacea, Peracarida). Zoologica Scripta 9: 271–281. 10.1111/j.1463-6409.1980.tb00667.x

[B39] Kobusch W (1998) The foregut of the Mysida (Crustacea, Peracarida) and its phylogenetic relevance. Philosophical Transactions of the Royal Society. B. Biological Sciences 353: 559–581. 10.1098/rstb.1998.0227

[B40] Larkin MA Blackshields G Brown NP Chenna R McGettigan PA McWilliam H Valentin F Wallace IM Wilm A Lopez R Thompson JD Gibson TJ Higgins DG (2007) Clustal W and Clustal X version 2.0. Bioinformatics 23: 2947–2948. 10.1093/bioinformatics/btm404 17846036

[B41] Lasenby DC Langford RR (1972) Growth, life history and respiration of *Mysisrelicta* in an Arctic and temperate lake. Journal of the Fisheries Research Board of Canada 29: 1701–1708. 10.1139/f72-270

[B42] Ledoyer M (1969) Sur divers crustacés antarctiques (leptostracés, cumacés, mysidacés et caridés) recueillis en Terre Adélie en 1961–1963 et 1964–1965. Crustaceana 17: 88–96. 10.1163/156854069X00088

[B43] Ledoyer M (1989) Les Mysidacés (Crustacea) des grottes sous-marines obscures de Méditerranée nord-occidentale et du proche Atlantique (Portugal et Madère). Marine Nature 2: 39–62.

[B44] Ledoyer M (1995) Mysidacés (Crustacea) de Kerguelen, Crozet et Bouvet (0céan Austral) récoltés par la Japonaise, le Marion-Dufresne (1972–82) et dans des contenus stomacaux d’oiseaux. Journal of Natural History 29: 601–618. 10.1080/00222939500770211

[B45] Lefort V Longueville JE Gascuel O (2017) SMS: Smart Model Selection in PhyML. Molecular Biology & Evolution 34: 2422–2424. 10.1093/molbev/msx149 28472384 PMC5850602

[B46] Lejeusne C Chevaldonné P (2005) Population structure and life history of *Hemimysismargalefi* (Crustacea: Mysidacea), a “thermophilic” cave-dwelling species benefiting from the warming of the NW Mediterranean. Marine Ecology Progress Series 287: 189–199. 10.3354/meps287189

[B47] Liljeborg W (1853) Hafs-Crustaceer vid Kullaberg. Öfversigt af Kongliga Vetenskaps-Akademiens Förhandlingar 9(1852): 1–13.

[B48] Lüdecke C (Ed.) (2013) Zum Kontinent des eisigen Südens. Die erste deutsche Südpolarexpedition 1901–1903 – Erich von Drygalski. Marix Verlag, Wiesbaden 5–414.

[B49] Mauchline J (1973) Intermoult growth of species of Mysidacea (Crustacea). Journal of the Marine Biological Association of the United Kingdom 53: 569–572. 10.1017/S002531540005877X

[B50] Mauchline J (1980) The biology of mysids and euphausiids. In: Blaxter JHS Russell FS Young M (Eds) Advances in Marine Biology.Academic Press, London 18: 1–677.

[B51] Mees J Meland K (2021) World List of Lophogastrida, Stygiomysida and Mysida. World Register of Marine Species. http://www.marinespecies.org/index.php [Accessed 8 Apr to 2 July 2021]

[B52] Meland K (2004) Species diversity and phylogeny of the deep-sea genus *Pseudomma* (Crustacea: Mysida). Zootaxa 649: 1–30. 10.11646/zootaxa.649.1.1

[B53] Meland K Brattegard T (1995) Redescription of the North Atlantic *Pseudomma* species (Crustacea, Mysidacea, with the addition of *Pseudommajasi* n.sp. Sarsia 80: 107–144. 10.1080/00364827.1995.10413585

[B54] Meland K Brattegard T (2007) New Mysida (Crustacea) in the genera *Amblyops* and *Pseudomma* from the Iceland Basin. Zootaxa 1628: 43–58. 10.11646/zootaxa.1628.1.3

[B55] Meland K Willassen E (2004) Molecular phylogeny and biogeography of the genus *Pseudomma* (Peracarida: Mysida). Journal of Crustacean Biology 24: 541–557. 10.1651/C-2481

[B56] Müller H-G (1993) World catalogue and bibliography of the recent Mysidacea. Wissenschaftlicher Verlag H.-G. Müller, Wetzlar, 491 pp. 10.1007/978-1-4471-3802-0_20

[B57] Murano M (1966) Two new species of *Pseudomma* (Mysidacea) from Sagami Bay, Central Japan. The Journal of the Oceanographical Society of Japan 22: 41–49. 10.5928/kaiyou1942.22.41

[B58] Murano M (1974) Mysidacea from the Central and Western Pacific I. The genus *Pseudomma* (tribe Erythropini). Publications of the Seto Marine Biological Laboratory 21: 287–334. 10.5134/175878

[B59] Murano M Krygier EE (1985) Bathypelagic mysids from the northeastern Pacific. Journal of Crustacean Biology 5: 686–706. 10.2307/1548246

[B60] Murano M Mauchline J (1999) Deep-sea mysids from the North Atlantic Ocean with description of four new species. Crustaceana 72: 273–295. 10.1163/156854099503366

[B61] Norman AM (1892) On British Mysidae, a family of CrustaceaSchizopoda. Annals and Magazine of Natural History, ser. 6, vol. 10(56): 143–166. 10.1080/00222939208677385

[B62] Nouvel H Lagardère J-P (1976) Les Mysidacés du talus continental du Golfe de Gascogne. I. Tribu des Erythropini (genre *Erythrops* excepté). Bulletin du Muséum national d’Histoire naturelle 3e sér. no. 414, Zoologie 291: 1243–1324.

[B63] O’Brien DP (1986) A new species of *Mysidetes* (Mysidacea, Leptomysini) from a marine cave in Waterfall Bay, Tasmania. Crustaceana 51: 254–258. 10.1163/156854086X00403

[B64] Peck LS (2018) Antarctic marine biodiversity: adaptations, environments and responses to change. Oceanography and Marine Biology. An Annual Review 56: 105–236. 10.1201/9780429454455-3

[B65] Pérez T Albenga L Starmer J Chevaldonné P (2016) Biodiversité des grottes sous-marines des îles Marquises : un patrimoine naturel caché et méconnu. In: Galzin R Duron S-D Meyer J-Y (Eds) Biodiversité terrestre et marine des îles Marquises, Polynésie Française.Société Française d’Ichtyologie, Paris, 287–310.

[B66] Petryashov VV (2006) Mysids (Crustacea, Mysidacea) of the Antarctic and Subantarctic from the collection of the Zoological Institute, Russian Academy of Sciences. Mysida: Erythropini and Amblyopsini tribes. Zoologicheskij Zhurnal 85: 1402–1421.

[B67] Petryashov VV (2007) Biogeographical division of Antarctic and Subantarctic by mysid (Crustacea: Mysidacea) fauna. Russian Journal of Marine Biology 33: 1–16. 10.1134/S1063074007010014

[B68] Petryashov VV (2014) 5.16. Lophogastrida and Mysida (Crustacea: Malacostraca: Peracarida) of the Southern Ocean. In: De Broyer C, Koubbi P, Griffiths HJ, Raymond B, d’Udekem d’Acoz C et al. (Eds) Biogeographic Atlas of the Southern Ocean (CoML/CAML Atlas). Census of Antarctic Marine Life. SCAR-Marine Biodiversity Information Network. Scientific Commitee on Antarctic Research, Cambridge, 149–154. http://share.biodiversity.aq/Atlas/PDFS/Atlas_Chap.5.16-Petryashov_2014-F.pdf [Accessed 30 Aug. 2018]

[B69] Pettibone MH (1997) Revision of the scaleworm genus *Eulagisca* McIntosh (Polychaeta: Polynoidae) with the erection of the subfamily Eulagiscinae and the new genus *Pareulagisca*. Proceedings of the Biological Society of Washington 110: 537–551.

[B70] Kemal M Kızıldağ S Koçak AÖ (2019) Further notes on the Heterocera (Lepidoptera) of the Philippines, with a description of a new species. Priamus 18(2): 31–128.

[B71] Parsons DG Savard L (1989) Some observations on modal analysis of shrimp length frequency distributions. In: Scientific Council - 1989. Working Group on Shrimp Ageing - October 1989, NAFO SCR Doc. 89/93. Northwest Atlantic Fisheries Organization, Serial No. N1693, 6 pp.

[B72] Price WW (2001) Mysinae. In: World list of Mysidacea. NeMys doc_id: 3677. http://www.marinespecies.org/aphia.php?p=sourcedetails&id=4191 [Accessed 23 July 2002]

[B73] Pyrzanowski K Zięba G Dukowska M Smith C Przybylski M (2019) The role of detritivory as a feeding tactic in a harsh environment – a case study of weatherfish (*Misgurnusfossilis*). Scientific Reports 9: e8467. 10.1038/s41598-019-44911-y PMC6559962 31186507

[B74] Rastorgueff P-A Harmelin-Vivien M Richard P Chevaldonné P (2011) Feeding strategies and resource partitioning mitigate the effects of oligotrophy for marine cave mysids. Marine Ecology Progress Series 440: 163–176. 10.3354/meps09347

[B75] Richoux NB Deibel D Thompson RJ (2004) Population biology of hyperbenthic crustaceans in a cold water environment (Conception Bay, Newfoundland). I. *Mysismixta* (Mysidacea). Marine Biology 144: 881–894. 10.1007/s00227-003-1249-7

[B76] San Vicente C (2011a) Species Diversity of Antarctic Mysids (Crustacea: Lophogastrida and Mysida). In: Mulder TJ (Ed.) Antarctica: Global, Environmental and Economic Issues.Nova Science Publishers, Chapter 1, 1–80.

[B77] San Vicente C (2011b) New Mysida (Crustacea) in the genus *Pseudomma* from the Bellingshausen Sea (Southern Ocean). Zootaxa 2833: 15–28. 10.11646/zootaxa.2833.1.2

[B78] San Vicente C (2017) Geographical and bathymetric distribution of mysids (Crustacea: Mysida) in the seas of the Iberian Peninsula. Zootaxa 4244: 151–194. 10.11646/zootaxa.4244.2 28610118

[B79] San Vicente C Ramos A Sorbe J-C (2006) Suprabenthic euphausiids and mysids from the South Shetland Islands and the Bransfield Strait, Southern Ocean (BENTART-95 cruise). Polar Biology 29: 211–222. 10.1007/s00300-005-0041-1

[B80] Sars GO (1864) V. Beretning om en i Sommeren 1863 foretagen zoologisk Reise i Christiania Stift. Nyt Magazin for Naturvidenskaberne 13: 225–260.

[B81] Sars GO (1869) VII. Undersøgelser over Christianiafjordens Dybvandsfauna. Nyt Magazin for Naturvidenskaberne 16: 305–362.

[B82] Sars GO (1870a) Nye Dybvandscrustaceer fra Lofoten. Forhandlinger i Videnskabs-Selskabet 1869: 147–174.

[B83] Sars GO (1870b) Carcinologiske Bidrag til Norges Fauna. I. Monographi over de ved Norges Kyster forekommende Mysider. Brøoger & Christie’s Bogtrykkery, Christiania, pt. 1: 1–64. [pls I–V] 10.5962/bhl.title.10408

[B84] Sars GO (1872) Carcinologiske Bidrag til Norges fauna. I. Monographi over de ved Norges Kyster forekommende Mysider. Brøoger & Christie’s Bogtrykkery, Christiania Pt. 2. 1–34. [pls VI–VIII]

[B85] Sars GO (1884) Preliminary notice on the Schizopoda of H. M. S. Challenger expedition. Forhandlinger i Videnskabs-Selskabet 7: 1–43.

[B86] Sato H Murano M (1994) Adoption of larvae escaped from the marsupium in four mysid species. Umi. La mer (Bulletin de la Société franco-japonaise d’océanographie) 32: 71–74.

[B87] Siegel V Mühlenhardt-Siegel U (1988) On the occurrence and biology of some Antarctic Mysidacea (Crustacea). Polar Biology 8: 181–190. 10.1007/BF00443451

[B88] Siewing R (1956) Untersuchungen zur Morphologie der Malacostraca (Crustacea). Zoologische Jahrbücher. Abteilung für Anatomie und Ontogenie der Tiere 75: 39–176.

[B89] Smith SI (1873) Crustacea. In: Verill AE (Ed.) Report upon the invertebrate animals of Vineyard Sound and the adjacent waters, with an account of the physical characters of the region.Report of U.S. Commissioner of Fish and Fisheries. Government Printing Office, Washington 1871–2 (part 1, no. 18), 545–580. [pls I–IX]

[B90] Stebbing TRR (1893) A history of the Crustacea. Recent Malacostraca. The International Scientific Series. D. Appleton & Co., New York, 71: [i–xvii,] 466 pp. [pls I–XIX] 10.5962/bhl.title.53964

[B91] Stephensen K (1910) Mysidácea (Mysider). In: Danmarks Fauna. Storkrebs. I. Skjoldkrebs. G.E.C.Gads Forlag, København 9: 122–149. 10.5962/bhl.title.14893

[B92] Tattersall OS (1955) Mysidacea. Discovery Reports. Cambridge University Press 28: 1–190. 10.5962/bhl.part.16838

[B93] Tattersall OS (1961) Report on some Mysidacea from the deeper waters of the Ross Sea. Proceedings of the Zoological Society of London 137: 553–571.

[B94] Tattersall OS (1965) The fauna of the Ross Sea, Pt. 4. Mysidacea. In: New Zealand Oceanographic Institute Memoir No. 27.New Zealand Department of Scientific and Industrial Research Bulletin 167: 9–25. [1 pl.]

[B95] Tattersall WM (1909) The Schizopoda collected by the Maia and Puritan in the Mediterranean. Mittheilungen aus der Zoologischen Station zu Neapel 19: 117–143. [Taf. 7]

[B96] Tattersall WM (1922) Indian Mysidacea. Records of the Indian Museum 24: 445–504.

[B97] Tattersall WM (1923) Crustacea. Part. VII. - Mysidacea. In: British Antarctic (Terra Nova) Expedition, 1910. Natural History Report.British Museum (Natural History), Zoology 3: 273–304.

[B98] Tattersall WM (1939) The Euphausiacea and Mysidacea of the John Murray Expedition to the Indian Ocean. John Murray Expedition 1933–1934. Scientific Reports. British Museum (Natural History), London 5: 203–246.

[B99] Tattersall WM (1951) A review of the Mysidacea of the United States National Museum. Bulletin of the United States National Museum 201: 1–292. 10.5962/bhl.part.16843

[B100] Tattersall WM Tattersall OS (1951) The British Mysidacea. Ray Society, London, monograph no. 136: 1–460. 10.5479/si.03629236.201.1

[B101] Tchindonova YuG (1981) New data on the systematic position of some deep-sea mysids (Mysidacea, Crustacea) and their distribution in the World Ocean. In: August 1979). Section Marine Biology. Biology of the Pacific Ocean Depths. Acad. Sci.USSR, Vladivostok 1: 24–33.

[B102] Vanhöffen E (1897) 1. Teil. Die Fauna und Flora Grönlands. In: Drygalski E von (Ed.) Grönland Expedition der Gesellschaft für Erdkunde zu Berlin, 1891–1893. W.H.Kühl, Berlin 2: 1–383. [8 pls]

[B103] Ward P (1984) Aspects of the biology of *Antarctomysismaxima* (Crustacea: Mysidacea). Polar Biology 3: 85–92. 10.1007/BF00258152

[B104] Ward P (1985) On the biology of *Antarctomysisohlini* (Crustacea: Mysidacea) at South Georgia. British Antarctic Survey Bulletin 67: 13–23.

[B105] Wilson GDF (1989) A systematic revision of the deep-sea subfamily Lipomerinae of the isopod crustacean family Munnopsidae. Bulletin of the Scripps Institution of Oceanography 27: 1–138.

[B106] Wittmann KJ (1978a) Biotop- und Standortbindung mediterraner Mysidacea. Dissertation zum Grad Dr. phil. an der Universität Wien, 211 pp.

[B107] Wittmann KJ (1978b) Adoption, replacement and identification of young in marine Mysidacea (Crustacea). Journal of Experimental Marine Biology and Ecology 32: 259–274. 10.1016/0022-0981(78)90120-X

[B108] Wittmann KJ (1981) Comparative biology and morphology of marsupial development in *Leptomysis* and other Mediterranean Mysidacea (Crustacea). Journal of Experimental Marine Biology and Ecology 52: 243–270. 10.1016/0022-0981(81)90040-X

[B109] Wittmann KJ (1984) Ecophysiology of marsupial development and reproduction in Mysidacea (Crustacea). Oceanography and Marine Biology. An Annual Review 22: 393–428.

[B110] Wittmann KJ (1996) Morphological and reproductive adaptations in Antarctic meso- to bathypelagic Mysidacea (Crustacea), with description of *Mysifaunerigens* n. g. sp. nov. In: Uiblein F Ott J Stachowitsch M (Eds) Deep-sea and extreme shallow-water habitats: affinities and adaptations. Biosystematics and Ecology Series.ÖAW, Wien 11: 221–231.

[B111] Wittmann KJ (2000) *Heteromysisarianii* sp.n., a new benthic mysid (Crustacea, Mysidacea) from coralloid habitats in the Gulf of Naples (Mediterranean Sea). Annalen des Naturhistorischen Museums in Wien 102B: 279–290.

[B112] Wittmann KJ (2013) Mysids associated with sea anemones from the tropical Atlantic: descriptions of *Ischiomysis* new genus, and two new species in this taxon (Mysida: Mysidae: Heteromysinae). Crustaceana 86: 487–506. 10.1163/15685403-00003166

[B113] Wittmann KJ (2018) Six new freshwater species of *Parvimysis*, with notes on breeding biology, statolith composition, and a key to the Mysidae (Mysida) of Amazonia. Crustaceana 91: 537–576. 10.1163/15685403-00003782

[B114] Wittmann KJ Abed-Navandi D (2021) Four new species of *Heteromysis* (Crustacea: Mysida) from public aquaria in Hawaii, Florida, and Western to Central Europe. European Journal of Taxonomy 735: 133–175. [video in suppl] 10.5852/ejt.2021.735.1247 34186968

[B115] Wittmann KJ Ariani AP (2019) Amazonia versus Pontocaspis: a key to understanding the mineral composition of mysid statoliths (Crustacea: Mysida). Biogeographia – The Journal of Integrative Biogeography 34: 1–15. [suppl. 1–34] 10.21426/B634142438

[B116] Wittmann KJ Ariani AP Lagardère J-P (2014) Chapter 54. Orders Lophogastrida Boas, 1883, Stygiomysida Tchindonova, 1981, and Mysida Boas, 1883 (also known collectively as Mysidacea). In: Vaupel Klein JC von Charmantier-Daures M Schram FR (Eds) Treatise on Zoology - Anatomy, Taxonomy, Biology.The Crustacea. Revised and updated, as well as extended from the Traité de Zoologie. Koninklijke Brill NV, Leiden 4B, 189–396. [colour plates 404–406] 10.1163/9789004264939_006

[B117] Wittmann KJ Chevaldonné P (2017) Description of Heteromysis (Olivemysis) ekamako sp. nov. (Mysida: Mysidae: Heteromysinae) from a marine cave at Nuku Hiva Island (Marquesas, French Polynesia, Pacific Ocean). Marine Biodiversity 47: 879–886. 10.1007/s12526-016-0522-1

[B118] Wittmann KJ Griffiths CL (2018) A new species of *Mysidopsis* G. O. Sars, 1864 from the Atlantic coast of South Africa, with supplementary descriptions of two additional species and notes on colour and feeding apparatus (Mysida: Mysidae). Journal of Crustacean Biology 38: 215–234. 10.1093/jcbiol/rux118

[B119] Wittmann KJ Schlacher TA Ariani AP (1993) Structure of recent and fossil mysid statoliths (Crustacea, Mysidacea). Journal of Morphology 215: 31–49. 10.1002/jmor.1052150103 29865427

[B120] Wittmann KJ Wirtz P (2017) *Heteromysissabelliphila* sp. nov. (Mysida: Mysidae: Heteromysinae) in facultative association with sabellids from the Cape Verde Islands (subtropical N.E. Atlantic). Crustaceana 90: 131–151. 10.1163/15685403-00003624

[B121] Wortham-Neal JL Price WW (2002) Marsupial developmental stages in *Americamysisbahia* (Mysida: Mysidae). Journal of Crustacean Biology 22: 98–112. 10.1163/20021975-99990213

[B122] Wright RA (1973) Occurrence and Distribution of the Mysidacea of the Gulf of St. Lawrence. Master Thesis at McGill University, Montreal, i–xiv, 1–163, appendix I–XIV.

[B123] Zimmer C (1914) Die Schizopoden der deutschen Südpolar-Expedition 1901–1903. In: Drygalski E von (Ed.) Deutsche Südpolar-Expedition 1901–1903, XV. Zoologie.Georg Reimer, Berlin 7: 377–445. [fig.-tabs XXIII–XXVI]

